# Supplementary material for: Consumption of Vitamin-A-Rich Foods and Vitamin A Supplementation for Children under Two Years Old in 51 Low- and Middle-Income Countries
Source: Nutrients. 2021 Dec 31;14(1):188. doi: 10.3390/nu14010188 (PMC8747127; doi:10.3390/nu14010188)
Supplement: Supplementary file 1 [file nutrients-14-00188-s001.zip › nutrients-1515985-supplementary.pdf]

Supplementary material

Consumption of Vitamin-A-Rich Foods and Vitamin A Supplementation for Children under Two Years Old in 51 Low- and Middle-Income Countries

Table S1. Survey year, population size, sample size, and World Bank income group 3

Table S2. Sample for feeding and vitamin A supplementation of children 6–23 months old 5

Table S3. Sample for vitamin A supplementation of children 6–59 months old........ 7

Table S4. Percentage of children 6–23 months old receiving minimum dietary diversity, vitamin-A-rich foods, vitamin A supplement, and either vitamin-A-rich foods or vitamin A supplement........ 8

Table S5. Percentage of children 6–23 months old receiving minimum dietary diversity by sex........ 9

Table S6. Percentage of children 6–23 months old receiving vitamin-A-rich foods by sex........ 10

Table S7. Percentage of children 6–23 months old receiving vitamin A supplementation by sex........ 11

Table S8. Percentage of children 6–23 months old receiving either vitamin-A-rich foods or vitamin A supplementation by sex 13

Table S9. Percentage of children 6–23 months old receiving minimum dietary diversity by living standards 14

Table S10. Percentage of children 6–23 months old receiving vitamin-A-rich foods by living standards 15

Table S11. Percentage of children 6–23 months old receiving vitamin A supplementation by living standards 16

Table S12. Percentage of children 6–23 months old receiving either vitamin-A-rich foods or vitamin A supplementation by living standards 18

Table S13. Percentage of children 6–23 months old receiving minimum dietary diversity by urban-rural residency 19

Table S14. Percentage of children 6–23 months old receiving vitamin-A-rich foods by urban-rural residency 20

Table S15. Percentage of children 6–23 months old receiving vitamin A supplementation by urban-rural residency 21

Table S16. Percentage of children 6–23 months old receiving either vitamin-A-rich foods or vitamin A supplementation by urban-rural residency 22

Table S17. Percentage of children under two (6–23 months) and over two (24–59 months) receiving vitamin A supplementation24

Table S18. Percentage of children under two (6–23 months) and over two (24–59 months) receiving vitamin A supplementation by sex 26

Table S19. Percentage of children under two (6–23 months) and over two (24–59 months) receiving vitamin A supplementation by living standards 27

Table S20. Percentage of children under two (6–23 months) and over two (24–59 months) receiving vitamin A supplementation by urban-rural residency........ 29

Figure S1. Percentage of children 6–23 months old receiving minimum dietary diversity: by sex (showing relative differences)32

Figure S2. Percentage of children 6–23 months old receiving vitamin-A-rich foods: by sex (showing relative differences)........ 33

Figure S3. Percentage of children 6–23 months old receiving vitamin A supplementation: by sex (showing relative differences)34

Figure S4. Percentage of children 6–23 months old receiving either vitamin-A-rich foods or vitamin A supplementation: by sex (showing relative differences) 35

Figure S5. Percentage of children 6–23 months old receiving minimum dietary diversity: by sex and country (showing relative differences) 36

Figure S6. Percentage of children 6–23 months old receiving vitamin-A-rich foods: by sex and country (showing relative differences) 37

Figure S7. Percentage of children 6–23 months old receiving vitamin A supplementation: by sex and country (showing relative differences) 38

Figure S8. Percentage of children 6–23 months old receiving either vitamin-A-rich foods or vitamin A supplementation: by sex and country (showing relative differences) 39

Figure S9. Percentage of children 6–23 months old receiving minimum dietary diversity: by living standards (showing relative differences) 40

Figure S10. Percentage of children 6–23 months old receiving vitamin-A-rich foods: by living standards (showing relative differences) 41

Figure S11. Percentage of children 6–23 months old receiving vitamin A supplementation: by living standards (showing relative differences) 42

Figure S12. Percentage of children 6–23 months old receiving either vitamin-A-rich foods or vitamin A supplementation: by living standards (showing relative differences) 43

Figure S13. Percentage of children 6–23 months old receiving minimum dietary diversity: by living standards and country (showing relative differences) 44

Figure S14. Percentage of children 6–23 months old receiving vitamin-A-rich foods: by living standards and country (showing relative differences) 45

Figure S15. Percentage of children 6–23 months old receiving vitamin A supplementation: by living standards and country (showing relative differences) 46

Figure S16. Percentage of children 6–23 months old receiving either vitamin-A-rich foods or vitamin A supplementation: by living standards and country (showing relative differences) 47

Figure S17. Percentage of children 6–23 months old receiving minimum dietary diversity: by urban-rural residency (showing relative differences) 48

Figure S18. Percentage of children 6–23 months old receiving vitamin-A-rich foods: by urban-rural residency (showing relative differences) 49

Figure S19. Percentage of children 6–23 months old receiving vitamin A supplementation: by urban-rural residency (showing relative differences) 50

Figure S20. Percentage of children 6–23 months old receiving either vitamin-A-rich foods or vitamin A supplementation: by urban-rural residency (showing relative differences) 51

Figure S21. Percentage of children 6–23 months old receiving minimum dietary diversity: by urban-rural residency and country (showing relative differences) 52

Figure S22. Percentage of children 6–23 months old receiving vitamin-A-rich foods: by urban-rural residency and country (showing relative differences) 53

Figure S23. Percentage of children 6–23 months old receiving vitamin A supplementation: by urban-rural residency and country (showing relative differences) 54

Figure S24. Percentage of children 6–23 months old receiving either vitamin-A-rich foods or vitamin A supplementation: by urban-rural residency and country (showing relative differences) 55

Figure S25. Percentage of children under two (6–23 months) and over two (24–59 months) receiving vitamin A supplementation: by sex 56

Figure S26. Percentage of children under two (6–23 months) and over two (24–59 months) receiving vitamin A supplementation: by living standards........ 57

Figure S27. Percentage of children under two (6–23 months) and over two (24–59 months) receiving vitamin A supplementation: by urban-rural residency 58

Figure S28. Percentage of children under two (6–23 months) and over two (24–59 months) receiving vitamin A supplementation: by sex and country 59

Figure S29. Percentage of children under two (6–23 months) and over two (24–59 months) receiving vitamin A supplementation: by living standards and country........ 60

Figure S30. Percentage of children under two (6–23 months) and over two (24–59 months) receiving vitamin A supplementation: by urban-rural residency and country 61

Table S1. Survey year, population size, sample size, and World Bank income group.

Country	Income group	Survey year	Population under-5 ('000)	Sample size
Pooled			347,845	753,643
Low-Income Countries			107,334	283,597
Lower-Middle-Income Countries			232,102	438,406
Upper-Middle-Income Countries			8,408	31,640
East Asia and Pacific			29,332	45,899
Cambodia	Low	2010–2011	1,670	7,820
Indonesia	Lower Middle	2012	22,330	17,367
Myanmar	Lower Middle	2015–2016	4,079	4,597
Papua New Guinea	Lower Middle	2016–2018	1,089	9,159
Timor-Leste	Lower Middle	2016	164	6,956
Eastern and Southern Africa			55,943	119,217
Burundi	Low	2010–2011	1,619	7,231
Comoros	Low	2012	114	3,022
Ethiopia	Low	2011	14,806	10,808
Kenya	Low	2014	6,515	20,093
Lesotho	Lower Middle	2014	243	2,915
Malawi	Low	2010	2,583	18,360
Mozambique	Low	2011	4,494	10,291
Namibia	Upper Middle	2013	294	4,818
Rwanda	Low	2010–2011	1,601	8,484
South Africa	Upper Middle	2016	5,871	3,413
Tanzania	Low	2015–2016	8,597	9,713
Uganda	Low	2011	6,576	7,355
Zambia	Lower Middle	2013–2014	2,630	12,714
Europe and Central Asia			1,755	9,085
Kyrgyz Republic	Low	2012	629	4,247
Tajikistan	Low	2012	1,126	4,838
Latin America and Caribbean			5,143	33,886
Dominican Republic	Upper Middle	2013	984	4,479
Guatemala	Lower Middle	2014–2015	1,992	12,071
Haiti	Low	2012	1,204	6,744
Honduras	Lower Middle	2011–2012	963	10,592
Middle East and North Africa			16,500	40,977
Egypt	Lower Middle	2014	11,717	15,466
Jordan	Upper Middle	2012	1,012	10,128
Yemen	Lower Middle	2013	3,771	15,383
South Asia			163,503	306,193
Afghanistan	Low	2015	5,366	31,063
Bangladesh	Low	2011	15,263	8,343
India	Lower Middle	2015–2016	114,758	247,743
Maldives	Upper Middle	2016–2017	33	3,055
Nepal	Low	2010–2011	3,074	5,054
Pakistan	Lower Middle	2012–2013	25,009	10,935
West and Central Africa			75,668	198,386
Benin	Low	2011–2012	1,572	12,679
Burkina Faso	Low	2010	2,902	13,716
Cameroon	Lower Middle	2011	3,529	10,734
Chad	Low	2014–2015	2,557	16,901

Country	Income group	Survey year	Population under-5 ('000)	Sample size
Pooled			347,845	753,643
Congo	Lower Middle	2011–2012	628	8,857
Congo Democratic Republic	Low	2013–2014	13,155	17,228
Cote d'Ivoire	Lower Middle	2011–2012	3,299	7,093
Gabon	Upper Middle	2012	214	5,747
Gambia	Low	2013	337	7,788
Ghana	Lower Middle	2014	3,765	5,595
Guinea	Low	2012	1,835	6,424
Liberia	Low	2013	576	7,058
Mali	Low	2012–2013	3,088	9,582
Niger	Low	2012	3,899	11,602
Nigeria	Lower Middle	2013	30,126	28,596
Senegal	Lower Middle	2010–2011	2,011	11,633
Sierra Leone	Low	2013	1,103	10,618
Togo	Low	2013–2014	1,072	6,535

Notes: Sampling weights were scaled to sum up to under-5 population size for the complete sample. Sample sizes shows all children under-5 alive at the time of survey.

Table S2. Sample for feeding and vitamin A supplementation of children 6–23 months old.

Country	Sample size	<6 or >23 months old, excluded	+Not last born, excluded	Total eligible	Missing information on diet, excluded	Missing information on vitamin-A-rich foods, excluded	Missing information on vitamin A supplementation, excluded	Valid observations for all feeding variables
Pooled	753,643	524,219	11,235	218,189	4,215	4,679	5,648	208,008 (95%)
Low-Income Countries	283,597	197,912	4,046	81,639	3,528	3,782	1,146	76,790 (94%)
Lower-Middle-Income Countries	438,406	304,347	6,309	127,750	608	809	2,558	124,445 (97%)
Upper-Middle-Income Countries	31,640	21,960	880	8,800	79	88	1,944	6,773 (77%)
East Asia and Pacific	45,899	31,764	721	13,414	218	250	273	12,915 (96%)
Cambodia	7,820	5,380	77	2,363	25	9	8	2,346 (99%)
Indonesia	17,367	11,955	223	5,189	86	113	111	4,966 (96%)
Myanmar	4,597	3,216	42	1,339	2	1	7	1,331 (99%)
Papua New Guinea	9,159	6,416	198	2,545	89	108	142	2,317 (91%)
Timor-Leste	6,956	4,797	181	1,978	16	19	5	1,955 (99%)
Eastern and Southern Africa	119,217	82,456	2,030	34,731	3,170	3,272	193	31,291 (90%)
Burundi	7,231	4,956	90	2,185	4	15	2	2,168 (99%)
Comoros	3,022	2,070	93	859	23	33	28	799 (93%)
Ethiopia	10,808	7,838	139	2,831	12	18	20	2,794 (99%)
Kenya	20,093	13,969	361	5,763	2,986	3,004	30	2,747 (48%)
Lesotho	2,915	1,912	57	946	13	15	12	920 (97%)
Malawi	18,360	12,487	215	5,658	38	46	6	5,607 (99%)
Mozambique	10,291	6,871	164	3,256	15	23	14	3,220 (99%)
Namibia	4,818	3,306	215	1,297	11	12	21	1,264 (97%)
Rwanda	8,484	6,063	100	2,321	8	14	2	2,305 (99%)
South Africa	3,413	2,431	111	871	7	10	29	833 (96%)
Tanzania	9,713	6,570	168	2,975	3	3	12	2,960 (99%)
Uganda	7,355	5,137	155	2,063	9	18	8	2,038 (99%)
Zambia	12,714	8,846	162	3,706	41	61	9	3,636 (98%)
Europe and Central Asia	9,085	6,107	197	2,781	15	40	186	2,557 (92%)
Kyrgyz Republic	4,247	2,841	90	1,316	5	19	79	1,219 (93%)
Tajikistan	4,838	3,266	107	1,465	10	21	107	1,338 (91%)
Latin America and Caribbean	33,886	23,334	554	9,998	51	79	39	9,880 (99%)
Dominican Republic	4,479	3,077	98	1,304	14	13	14	1,277 (98%)
Guatemala	12,071	8,433	138	3,500	3	15	1	3,484 (100%)
Haiti	6,744	4,608	175	1,961	9	15	7	1,939 (99%)
Honduras	10,592	7,216	143	3,233	25	36	17	3,180 (98%)
Middle East and North Africa	40,977	28,234	877	11,866	55	83	1,930	9,858 (83%)

Country	Sample size	<6 or >23 months old, excluded	+Not last born, excluded	Total eligible	Missing information on diet, excluded	Missing information on vitamin-A-rich foods, excluded	Missing information on vitamin A supplementation, excluded	Valid observations for all feeding variables
Egypt	15,466	10,359	296	4,811	3	11	41	4,759 (99%)
Jordan	10,128	7,096	258	2,774	2	3	1,824	950 (34%)
Yemen	15,383	10,779	323	4,281	50	69	65	4,149 (97%)
South Asia	306,193	215,158	3,884	87,151	226	310	1,324	85,561 (98%)
Afghanistan	31,063	22,587	430	8,046	115	179	365	7,532 (94%)
Bangladesh	8,343	5,934	49	2,360	8	10	27	2,323 (98%)
India	247,743	173,006	3,111	71,626	94	105	869	70,662 (99%)
Maldives	3,055	2,181	27	847	2	3	14	830 (98%)
Nepal	5,054	3,598	36	1,420	1	1	5	1,414 (100%)
Pakistan	10,935	7,852	231	2,852	6	12	44	2,800 (98%)
West and Central Africa	198,386	137,166	2,972	58,248	480	645	1,703	55,946 (96%)
Benin	12,679	8,782	191	3,706	61	69	130	3,518 (95%)
Burkina Faso	13,716	9,472	114	4,130	13	14	10	4,107 (99%)
Cameroon	10,734	7,223	255	3,256	30	59	1,111	2,103 (65%)
Chad	16,901	12,349	182	4,370	32	52	84	4,236 (97%)
Congo	8,857	5,989	165	2,703	24	25	11	2,667 (99%)
Congo Democratic Republic	17,228	11,998	283	4,947	32	51	35	4,862 (98%)
Cote d'Ivoire	7,093	4,855	110	2,128	14	21	24	2,083 (98%)
Gabon	5,747	3,869	171	1,707	43	47	42	1,619 (95%)
Gambia	7,788	5,273	103	2,412	10	25	18	2,369 (98%)
Ghana	5,595	3,876	80	1,639	1	1	7	1,631 (100%)
Guinea	6,424	4,416	66	1,942	9	15	7	1,920 (99%)
Liberia	7,058	4,767	146	2,145	6	11	10	2,125 (99%)
Mali	9,582	6,699	106	2,777	16	17	24	2,736 (99%)
Niger	11,602	8,225	137	3,240	19	25	46	3,172 (98%)
Nigeria	28,596	19,516	411	8,669	108	134	53	8,486 (98%)
Senegal	11,633	8,101	183	3,349	3	4	29	3,316 (99%)
Sierra Leone	10,618	7,353	196	3,069	40	49	55	2,970 (97%)
Togo	6,535	4,403	73	2,059	19	26	7	2,026 (98%)

Notes: Sample sizes shows all children under-5 alive at the time of survey. Valid observations show number of observations used for analysis.

Table S3. Sample for vitamin A supplementation of children 6–59 months old.

Country	Sample size	Excluded <6 months	Total eligible	Valid observations
Pooled	753,643	74,929	678,714	657,159 (97%)
Low-Income Countries	283,597	29,295	254,302	248,099 (98%)
Lower-Middle-Income Countries	438,406	42,566	395,840	386,559 (98%)
Upper-Middle-Income Countries	31,640	3,068	28,572	22,501 (79%)
East Asia and Pacific	45,899	4,447	41,452	40,000 (96%)
Cambodia	7,820	723	7,097	7,013 (99%)
Indonesia	17,367	1,710	15,657	15,215 (97%)
Myanmar	4,597	474	4,123	4,079 (99%)
Papua New Guinea	9,159	835	8,324	7,469 (90%)
Timor-Leste	6,956	705	6,251	6,224 (100%)
Eastern and Southern Africa	119,217	11,760	107,457	105,648 (98%)
Burundi	7,231	688	6,543	6,508 (99%)
Comoros	3,022	332	2,690	2,530 (94%)
Ethiopia	10,808	1,211	9,597	9,428 (98%)
Kenya	20,093	1,837	18,256	18,022 (99%)
Lesotho	2,915	339	2,576	2,505 (97%)
Malawi	18,360	1,702	16,658	16,572 (99%)
Mozambique	10,291	1,070	9,221	9,139 (99%)
Namibia	4,818	539	4,279	4,040 (94%)
Rwanda	8,484	724	7,760	7,722 (100%)
South Africa	3,413	337	3,076	2,888 (94%)
Tanzania	9,713	976	8,737	8,530 (98%)
Uganda	7,355	797	6,558	6,393 (97%)
Zambia	12,714	1,208	11,506	11,371 (99%)
Europe and Central Asia	9,085	879	8,206	7,642 (93%)
Kyrgyz Republic	4,247	452	3,795	3,552 (94%)
Tajikistan	4,838	427	4,411	4,090 (93%)
Latin America and Caribbean	33,886	3,422	30,464	30,027 (99%)
Dominican Republic	4,479	400	4,079	3,995 (98%)
Guatemala	12,071	1,184	10,887	10,866 (100%)
Haiti	6,744	737	6,007	5,828 (97%)
Honduras	10,592	1,101	9,491	9,338 (98%)
Middle East and North Africa	40,977	4,069	36,908	31,341 (85%)
Egypt	15,466	1,515	13,951	13,844 (99%)
Jordan	10,128	870	9,258	4,059 (44%)
Yemen	15,383	1,684	13,699	13,438 (98%)
South Asia	306,193	28,740	277,453	272,149 (98%)
Afghanistan	31,063	3,234	27,829	26,198 (94%)
Bangladesh	8,343	802	7,541	7,457 (99%)
India	247,743	22,842	224,901	221,553 (99%)
Maldives	3,055	276	2,779	2,732 (98%)
Nepal	5,054	499	4,555	4,524 (99%)
Pakistan	10,935	1,087	9,848	9,685 (98%)
West and Central Africa	198,386	21,612	176,774	170,352 (96%)
Benin	12,679	1,257	11,422	10,954 (96%)
Burkina Faso	13,716	1,472	12,244	12,176 (99%)
Cameroon	10,734	1,152	9,582	6,942 (72%)
Chad	16,901	1,843	15,058	14,615 (97%)
Congo	8,857	974	7,883	7,752 (98%)
Congo Democratic Republic	17,228	1,973	15,255	14,984 (98%)
Cote d'Ivoire	7,093	794	6,299	6,079 (97%)
Gabon	5,747	646	5,101	4,787 (94%)
Gambia	7,788	970	6,818	6,661 (98%)

Country	Sample size	Excluded <6 months	Total eligible	Valid observations
Ghana	5,595	613	4,982	4,892 (98%)
Guinea	6,424	732	5,692	5,610 (99%)
Liberia	7,058	729	6,329	6,138 (97%)
Mali	9,582	1,016	8,566	8,450 (99%)
Niger	11,602	1,321	10,281	10,017 (97%)
Nigeria	28,596	2,989	25,607	25,268 (99%)
Senegal	11,633	1,360	10,273	10,039 (98%)
Sierra Leone	10,618	1,154	9,464	9,133 (97%)
Togo	6,535	617	5,918	5,855 (99%)

Notes: Sample sizes shows all children under-5 alive at the time of survey. Valid observations show number of observations used for analysis.

Table S4. Percentage of children 6–23 months old receiving minimum dietary diversity, vitamin-A-rich foods, vitamin A supplement, and either vitamin-A-rich foods or vitamin A supplement.

	Minimum dietary diversity	Vitamin-A-rich foods	Vitamin A supplementation	Vitamin-A-rich foods or supplementation
Pooled	22.3 [21.9, 22.8]	54.9 [54.3, 55.4]	58.7 [58.1, 59.3]	78.3 [77.9, 78.7]
Low-Income Countries	18.2 [17.6, 18.9]	58.7 [57.6, 59.8]	59.6 [58.6, 60.5]	80.1 [79.3, 80.8]
Lower-Middle-Income Countries	23.6 [23.1, 24.2]	52.7 [52.0, 53.3]	58.0 [57.2, 58.7]	77.2 [76.6, 77.7]
Upper-Middle-Income Countries	38.9 [35.9, 41.8]	72.0 [69.0, 74.8]	70.2 [67.2, 73.0]	90.4 [88.4, 92.0]
East Asia and Pacific	46.6 [44.7, 48.4]	81.5 [80.1, 82.9]	65.0 [63.1, 66.7]	90.1 [89.0, 91.1]
Cambodia	29.9 [27.4, 32.6]	85.2 [83.0, 87.1]	66.0 [63.1, 68.7]	92.5 [91.0, 93.9]
Indonesia	53.3 [51.1, 55.5]	83.4 [81.6, 85.0]	68.8 [66.6, 70.8]	91.6 [90.4, 92.7]
Myanmar	21.3 [18.3, 24.7]	69.5 [65.8, 72.9]	49.6 [45.4, 53.8]	81.1 [77.7, 84.1]
Papua New Guinea	31.5 [28.3, 34.9]	86.8 [84.6, 88.8]	40.6 [37.1, 44.1]	90.8 [88.8, 92.4]
Timor-Leste	26.8 [23.7, 30.2]	67.8 [64.7, 70.6]	61.8 [58.4, 65.1]	85.3 [82.9, 87.4]
Eastern and Southern Africa	19.6 [18.7, 20.6]	59.6 [57.9, 61.3]	62.2 [60.8, 63.5]	81.1 [79.8, 82.3]
Burundi	16.7 [14.7, 18.9]	83.5 [81.7, 85.2]	80.9 [78.7, 83.0]	96.0 [95.0, 96.8]
Comoros	21.6 [17.7, 26.1]	71.1 [66.6, 75.1]	50.8 [46.0, 55.6]	83.6 [80.1, 86.6]
Ethiopia	4.4 [3.5, 5.5]	25.7 [23.0, 28.6]	51.5 [48.4, 54.6]	61.9 [58.7, 64.9]
Kenya	36.4 [33.5, 39.4]	72.2 [69.7, 74.5]	76.6 [74.2, 78.9]	91.0 [89.4, 92.4]
Lesotho	17.1 [14.2, 20.4]	61.1 [56.7, 65.3]	73.3 [69.6, 76.6]	87.4 [84.8, 89.6]
Malawi	27.2 [25.7, 28.9]	75.0 [73.4, 76.5]	84.3 [82.9, 85.6]	94.2 [93.3, 95.0]
Mozambique	28.2 [25.8, 30.7]	71.1 [68.9, 73.2]	70.3 [67.7, 72.8]	88.8 [87.2, 90.2]
Namibia	25.1 [21.7, 28.9]	71.3 [68.1, 74.3]	87.1 [84.7, 89.2]	94.7 [92.9, 96.0]
Rwanda	24.7 [22.6, 26.9]	72.8 [70.7, 74.7]	87.8 [86.1, 89.4]	94.2 [93.0, 95.2]
South Africa	38.4 [34.6, 42.4]	71.7 [67.6, 75.4]	78.3 [74.2, 81.8]	92.1 [89.4, 94.1]
Tanzania	21.1 [19.0, 23.4]	74.3 [71.6, 76.7]	43.2 [40.4, 45.9]	82.6 [80.7, 84.4]
Uganda	17.2 [15.1, 19.6]	62.5 [59.5, 65.5]	61.8 [58.5, 65.0]	83.5 [80.9, 85.8]
Zambia	18.3 [16.5, 20.2]	75.2 [73.4, 76.9]	68.7 [66.7, 70.7]	88.8 [87.5, 90.1]
Europe and Central Asia	35.8 [33.2, 38.4]	57.2 [54.5, 59.9]	54.4 [51.4, 57.5]	78.9 [76.4, 81.2]
Kyrgyz Republic	36.2 [32.1, 40.4]	65.5 [61.3, 69.4]	44.0 [39.9, 48.2]	81.1 [77.6, 84.2]
Tajikistan	35.5 [32.3, 38.9]	52.5 [49.1, 55.8]	60.4 [56.4, 64.3]	77.6 [74.2, 80.7]
Latin America and Caribbean	48.4 [46.8, 50.0]	76.0 [74.8, 77.2]	58.1 [56.4, 59.7]	88.1 [87.0, 89.0]
Dominican Republic	46.6 [42.4, 50.9]	73.8 [70.3, 77.0]	33.3 [29.9, 36.9]	82.1 [79.2, 84.8]
Guatemala	58.3 [56.1, 60.4]	80.3 [78.6, 81.9]	61.1 [58.6, 63.5]	90.4 [89.1, 91.5]
Haiti	23.2 [20.1, 26.6]	65.7 [62.7, 68.5]	54.9 [51.0, 58.7]	82.6 [79.6, 85.2]
Honduras	60.9 [58.7, 63.2]	82.1 [80.5, 83.7]	80.2 [78.2, 82.1]	95.8 [94.9, 96.6]
Middle East and North Africa	32.0 [30.6, 33.4]	58.3 [56.8, 59.8]	28.0 [26.5, 29.4]	69.3 [67.9, 70.7]
Egypt	34.7 [33.0, 36.5]	61.1 [59.3, 62.9]	20.6 [19.1, 22.1]	68.3 [66.5, 69.9]
Jordan	43.7 [38.2, 49.3]	71.4 [66.4, 76.0]	52.9 [47.4, 58.3]	89.2 [85.7, 92.0]
Yemen	21.2 [19.3, 23.3]	47.1 [44.6, 49.5]	51.7 [49.2, 54.3]	71.3 [69.2, 73.3]
South Asia	19.8 [19.3, 20.4]	46.1 [45.4, 46.8]	61.4 [60.6, 62.1]	76.3 [75.7, 76.9]
Afghanistan	16.7 [14.1, 19.7]	47.3 [43.2, 51.4]	49.6 [44.2, 55.0]	71.9 [68.6, 75.0]

	Minimum dietary diversity	Vitamin-A-rich foods	Vitamin A supplementation	Vitamin-A-rich foods or supplementation
Bangladesh	23.8 [21.7, 25.9]	64.1 [61.7, 66.5]	51.9 [48.9, 54.9]	79.2 [76.9, 81.3]
India	19.6 [19.1, 20.2]	44.0 [43.4, 44.6]	60.6 [60.0, 61.3]	74.5 [74.0, 75.0]
Maldives	69.3 [64.7, 73.5]	88.4 [84.9, 91.1]	55.0 [49.6, 60.2]	93.2 [90.1, 95.4]
Nepal	27.3 [23.2, 31.8]	46.7 [43.0, 50.5]	75.9 [72.7, 78.9]	83.2 [80.2, 85.8]
Pakistan	17.8 [15.6, 20.2]	44.6 [41.0, 48.2]	71.9 [68.4, 75.1]	83.5 [80.8, 85.8]
West and Central Africa	15.7 [14.9, 16.5]	57.5 [56.3, 58.6]	55.1 [53.6, 56.5]	77.3 [76.2, 78.4]
Benin	29.1 [27.1, 31.2]	53.3 [51.1, 55.6]	51.1 [48.7, 53.6]	73.1 [71.0, 75.0]
Burkina Faso	5.2 [4.3, 6.2]	34.4 [32.2, 36.8]	66.1 [63.4, 68.7]	75.5 [73.1, 77.7]
Cameroon	26.1 [23.2, 29.2]	76.3 [73.5, 79.0]	77.7 [74.7, 80.5]	95.3 [93.8, 96.4]
Chad	9.0 [7.6, 10.7]	45.5 [42.7, 48.4]	42.8 [40.1, 45.6]	65.9 [63.2, 68.4]
Congo	16.1 [13.7, 18.7]	76.6 [73.5, 79.4]	65.2 [61.0, 69.1]	91.0 [88.6, 92.9]
Congo Democratic Republic	17.3 [14.8, 20.2]	81.7 [79.9, 83.4]	68.5 [65.6, 71.3]	93.0 [91.7, 94.1]
Cote D'Ivoire	7.5 [6.1, 9.3]	59.8 [56.6, 63.0]	61.6 [57.7, 65.4]	81.6 [78.7, 84.1]
Gabon	17.4 [13.9, 21.6]	69.0 [65.4, 72.4]	56.8 [53.0, 60.5]	84.7 [81.7, 87.2]
Gambia	10.2 [8.1, 12.8]	47.5 [43.9, 51.2]	75.0 [71.6, 78.1]	85.6 [83.3, 87.7]
Ghana	24.6 [21.1, 28.6]	67.2 [63.5, 70.7]	70.4 [65.7, 74.8]	86.8 [83.6, 89.4]
Guinea	6.4 [4.9, 8.3]	27.2 [23.9, 30.7]	39.6 [35.5, 43.9]	52.8 [48.7, 56.9]
Liberia	8.2 [6.6, 10.3]	58.1 [55.5, 60.7]	62.7 [58.8, 66.4]	82.7 [79.9, 85.1]
Mali	18.5 [16.3, 20.9]	54.7 [51.6, 57.7]	58.2 [55.1, 61.4]	78.0 [75.6, 80.2]
Niger	8.4 [7.1, 9.8]	37.0 [34.3, 39.8]	61.4 [57.9, 64.7]	73.6 [70.7, 76.4]
Nigeria	15.6 [14.2, 17.1]	51.7 [49.7, 53.6]	40.9 [38.4, 43.5]	68.5 [66.4, 70.5]
Senegal	23.6 [20.9, 26.5]	62.8 [60.2, 65.3]	77.6 [75.1, 79.9]	90.1 [88.6, 91.4]
Sierra Leone	13.7 [11.6, 16.1]	46.1 [42.9, 49.4]	84.6 [82.1, 86.8]	89.6 [87.6, 91.3]
Togo	18.3 [16.1, 20.6]	68.6 [66.0, 71.0]	81.4 [78.7, 83.8]	92.6 [91.1, 93.8]

Notes: 95% confidence intervals are shown in brackets.

Table S5. Percentage of children 6–23 months old receiving minimum dietary diversity by sex.

	Females	Males	Males/Females
	%	%	Relative difference
Pooled	22.6 [22.0, 23.2]	22.1 [21.6, 22.6]	0.977 [0.949, 1.006]
Low-Income Countries	18.5 [17.7, 19.3]	18.0 [17.2, 18.8]	0.975 [0.924, 1.028]
Lower-Middle-Income Countries	23.9 [23.2, 24.7]	23.4 [22.7, 24.1]	0.978 [0.944, 1.013]
Upper-Middle-Income Countries	41.3 [36.8, 45.9]	36.6 [32.6, 40.9]	0.888 [0.753, 1.046]
East Asia and Pacific	47.0 [44.5, 49.5]	46.2 [43.8, 48.6]	0.983 [0.919, 1.051]
Cambodia	29.5 [26.1, 33.0]	30.3 [27.1, 33.8]	1.029 [0.887, 1.194]
Indonesia	54.1 [51.2, 57.0]	52.5 [49.6, 55.4]	0.970 [0.902, 1.043]
Myanmar	18.9 [15.3, 23.0]	23.3 [19.2, 28.0]	1.235 [0.963, 1.585]
Papua New Guinea	29.9 [25.8, 34.3]	32.9 [28.6, 37.6]	1.103 [0.917, 1.328]
Timor-Leste	28.2 [24.2, 32.7]	25.4 [21.8, 29.3]	0.899 [0.756, 1.068]
Eastern and Southern Africa	20.0 [18.8, 21.3]	19.2 [18.1, 20.5]	0.960 [0.889, 1.036]
Burundi	17.2 [14.7, 20.0]	16.2 [13.7, 19.1]	0.944 [0.774, 1.152]
Comoros	24.2 [18.5, 31.1]	19.4 [15.3, 24.2]	0.800 [0.592, 1.080]
Ethiopia	4.9 [3.5, 6.6]	3.9 [2.7, 5.6]	0.802 [0.496, 1.298]
Kenya	36.1 [32.5, 39.8]	36.8 [32.9, 40.8]	1.020 [0.891, 1.167]
Lesotho	18.2 [14.0, 23.3]	15.8 [12.2, 20.3]	0.869 [0.607, 1.245]
Malawi	27.6 [25.3, 29.9]	26.9 [25.0, 29.0]	0.977 [0.877, 1.088]
Mozambique	29.0 [25.8, 32.4]	27.3 [24.4, 30.4]	0.942 [0.817, 1.085]
Namibia	27.7 [23.3, 32.5]	22.4 [18.2, 27.3]	0.810 [0.647, 1.013]
Rwanda	25.2 [22.5, 28.1]	24.2 [21.6, 27.0]	0.959 [0.832, 1.107]
South Africa	41.0 [35.0, 47.2]	36.1 [30.8, 41.7]	0.881 [0.705, 1.100]
Tanzania	21.2 [18.4, 24.3]	21.1 [18.3, 24.2]	0.997 [0.831, 1.195]
Uganda	17.1 [14.4, 20.1]	17.3 [14.4, 20.7]	1.015 [0.811, 1.269]
Zambia	17.6 [15.4, 20.1]	19.0 [16.7, 21.5]	1.077 [0.919, 1.263]
Europe and Central Asia	37.3 [33.8, 41.0]	34.3 [31.1, 37.8]	0.920 [0.809, 1.046]

	Females	Males	Males/Females
	%	%	Relative difference
Kyrgyz Republic	36.6 [31.5, 41.9]	35.8 [30.4, 41.6]	0.980 [0.807, 1.190]
Tajikistan	37.8 [33.2, 42.6]	33.5 [29.4, 37.8]	0.886 [0.748, 1.049]
Latin America and Caribbean	48.4 [46.2, 50.5]	48.4 [46.4, 50.5]	1.001 [0.947, 1.059]
Dominican Republic	49.1 [43.1, 55.1]	44.2 [39.2, 49.4]	0.901 [0.772, 1.051]
Guatemala	57.7 [54.8, 60.5]	58.8 [55.8, 61.8]	1.019 [0.950, 1.093]
Haiti	23.2 [19.0, 28.1]	23.2 [19.4, 27.5]	1.001 [0.785, 1.276]
Honduras	60.4 [57.4, 63.3]	61.4 [58.4, 64.4]	1.017 [0.955, 1.084]
Middle East and North Africa	32.5 [30.6, 34.5]	31.5 [29.6, 33.4]	0.968 [0.895, 1.048]
Egypt	34.9 [32.6, 37.4]	34.6 [32.3, 36.9]	0.989 [0.903, 1.083]
Jordan	46.2 [37.5, 55.2]	41.3 [34.1, 48.8]	0.893 [0.680, 1.173]
Yemen	23.0 [20.4, 25.9]	19.5 [17.1, 22.1]	0.846 [0.724, 0.988]
South Asia	19.9 [19.2, 20.7]	19.7 [19.1, 20.5]	0.991 [0.943, 1.041]
Afghanistan	17.2 [14.1, 20.9]	16.2 [13.7, 19.1]	0.939 [0.806, 1.093]
Bangladesh	23.9 [21.1, 27.0]	23.6 [20.8, 26.7]	0.988 [0.831, 1.175]
India	19.9 [19.1, 20.7]	19.4 [18.7, 20.1]	0.975 [0.928, 1.025]
Maldives	68.2 [61.6, 74.1]	70.3 [63.7, 76.2]	1.031 [0.907, 1.173]
Nepal	26.7 [22.0, 32.0]	27.9 [23.1, 33.4]	1.045 [0.859, 1.271]
Pakistan	16.9 [14.0, 20.4]	18.6 [15.8, 21.7]	1.097 [0.872, 1.382]
West and Central Africa	16.3 [15.3, 17.3]	15.0 [14.1, 15.9]	0.919 [0.864, 0.977]
Benin	27.5 [25.0, 30.1]	30.6 [28.1, 33.2]	1.113 [0.999, 1.240]
Burkina Faso	5.5 [4.3, 7.0]	4.8 [3.8, 6.1]	0.875 [0.647, 1.183]
Cameroon	23.9 [20.2, 28.0]	28.4 [25.0, 32.0]	1.187 [0.998, 1.412]
Chad	8.8 [7.2, 10.8]	9.2 [7.5, 11.2]	1.040 [0.843, 1.283]
Congo	15.0 [12.1, 18.3]	17.2 [14.1, 20.8]	1.150 [0.895, 1.478]
Congo Democratic Republic	17.9 [15.0, 21.2]	16.8 [14.1, 19.9]	0.940 [0.800, 1.103]
Cote D'Ivoire	8.3 [6.5, 10.6]	6.7 [4.8, 9.3]	0.814 [0.561, 1.181]
Gabon	16.5 [12.5, 21.4]	18.3 [13.3, 24.6]	1.111 [0.765, 1.612]
Gambia	10.5 [7.6, 14.4]	9.9 [7.7, 12.6]	0.940 [0.671, 1.318]
Ghana	26.8 [21.8, 32.3]	22.6 [19.0, 26.8]	0.846 [0.683, 1.047]
Guinea	7.0 [5.0, 9.6]	5.9 [4.3, 8.1]	0.848 [0.583, 1.233]
Liberia	8.4 [5.8, 12.1]	8.1 [6.1, 10.6]	0.959 [0.597, 1.542]
Mali	17.9 [15.3, 20.9]	19.0 [16.3, 22.2]	1.064 [0.887, 1.275]
Niger	8.8 [7.3, 10.7]	7.9 [6.4, 9.7]	0.892 [0.710, 1.121]
Nigeria	16.9 [15.2, 18.7]	14.3 [12.8, 16.0]	0.848 [0.758, 0.947]
Senegal	23.6 [20.7, 26.7]	23.6 [20.0, 27.6]	1.002 [0.842, 1.191]
Sierra Leone	14.7 [12.0, 17.7]	12.6 [10.2, 15.5]	0.863 [0.683, 1.090]
Togo	17.5 [14.8, 20.7]	18.9 [16.2, 22.0]	1.079 [0.878, 1.326]

Notes: 95% confidence intervals are shown in brackets.

Table S6. Percentage of children 6–23 months old receiving vitamin-A-rich foods by sex.

	Females	Males	Males/Females
	%	%	Relative difference
Pooled	55.3 [54.6, 56.0]	54.5 [53.8, 55.1]	0.985 [0.972, 0.999]
Low-Income Countries	58.9 [57.7, 60.2]	58.5 [57.2, 59.7]	0.992 [0.971, 1.014]
Lower-Middle-Income Countries	53.0 [52.2, 53.9]	52.3 [51.6, 53.1]	0.987 [0.969, 1.005]
Upper-Middle-Income Countries	75.5 [71.6, 79.1]	68.7 [64.8, 72.3]	0.909 [0.850, 0.973]
East Asia and Pacific	82.0 [80.1, 83.7]	81.1 [79.1, 82.9]	0.989 [0.961, 1.019]
Cambodia	85.3 [82.1, 88.0]	85.1 [82.5, 87.4]	0.997 [0.958, 1.039]
Indonesia	84.0 [81.7, 85.9]	82.8 [80.4, 85.0]	0.986 [0.953, 1.021]
Myanmar	69.3 [64.1, 74.0]	69.7 [64.8, 74.1]	1.006 [0.918, 1.102]
Papua New Guinea	85.8 [82.6, 88.5]	87.7 [85.1, 89.9]	1.023 [0.985, 1.063]
Timor-Leste	66.0 [61.8, 70.0]	69.4 [65.8, 72.8]	1.052 [0.979, 1.130]
Eastern and Southern Africa	60.2 [58.1, 62.2]	59.0 [57.1, 61.0]	0.981 [0.948, 1.015]
Burundi	83.7 [81.1, 86.0]	83.4 [80.6, 85.8]	0.996 [0.955, 1.039]
Comoros	75.4 [69.4, 80.6]	67.3 [61.3, 72.8]	0.892 [0.802, 0.992]

	Females	Males	Males/Females
	%	%	Relative difference
Ethiopia	25.3 [22.0, 29.0]	26.0 [22.5, 29.9]	1.027 [0.861, 1.226]
Kenya	73.4 [70.3, 76.4]	71.0 [67.6, 74.1]	0.966 [0.913, 1.022]
Lesotho	61.3 [55.4, 67.0]	60.8 [55.3, 66.0]	0.991 [0.882, 1.114]
Malawi	75.6 [73.3, 77.7]	74.5 [72.2, 76.6]	0.985 [0.946, 1.026]
Mozambique	71.8 [68.9, 74.4]	70.5 [67.4, 73.3]	0.982 [0.931, 1.035]
Namibia	71.9 [67.7, 75.8]	70.6 [65.9, 75.0]	0.982 [0.903, 1.068]
Rwanda	74.1 [71.3, 76.7]	71.4 [68.5, 74.2]	0.964 [0.914, 1.016]
South Africa	76.7 [71.3, 81.4]	67.1 [62.0, 71.9]	0.875 [0.800, 0.957]
Tanzania	74.2 [71.0, 77.2]	74.3 [71.1, 77.3]	1.001 [0.954, 1.050]
Uganda	61.7 [57.6, 65.8]	63.4 [59.2, 67.3]	1.026 [0.939, 1.121]
Zambia	75.8 [73.4, 78.1]	74.6 [72.1, 77.0]	0.984 [0.942, 1.028]
Europe and Central Asia	58.9 [55.2, 62.5]	55.7 [52.4, 59.0]	0.947 [0.876, 1.023]
Kyrgyz Republic	67.6 [62.0, 72.7]	63.6 [58.4, 68.4]	0.940 [0.852, 1.038]
Tajikistan	54.0 [49.3, 58.6]	51.1 [46.8, 55.4]	0.947 [0.848, 1.058]
Latin America and Caribbean	76.2 [74.5, 77.9]	75.8 [74.2, 77.4]	0.995 [0.966, 1.024]
Dominican Republic	73.8 [68.8, 78.3]	73.8 [69.3, 77.9]	1.000 [0.920, 1.085]
Guatemala	81.6 [79.3, 83.7]	79.1 [76.7, 81.4]	0.970 [0.932, 1.009]
Haiti	64.4 [60.1, 68.4]	66.9 [62.9, 70.6]	1.039 [0.956, 1.129]
Honduras	82.7 [80.4, 84.8]	81.7 [79.3, 83.8]	0.987 [0.952, 1.024]
Middle East and North Africa	58.1 [56.1, 60.1]	58.4 [56.4, 60.4]	1.004 [0.960, 1.051]
Egypt	61.2 [58.8, 63.6]	61.1 [58.6, 63.5]	0.998 [0.947, 1.051]
Jordan	68.8 [61.3, 75.5]	73.9 [67.3, 79.6]	1.074 [0.944, 1.223]
Yemen	46.8 [43.4, 50.1]	47.4 [44.3, 50.4]	1.013 [0.928, 1.105]
South Asia	46.5 [45.5, 47.5]	45.7 [44.8, 46.6]	0.983 [0.958, 1.008]
Afghanistan	48.5 [43.9, 53.0]	46.1 [41.9, 50.4]	0.952 [0.888, 1.021]
Bangladesh	63.9 [60.6, 67.0]	64.4 [60.9, 67.7]	1.008 [0.939, 1.082]
India	44.7 [43.8, 45.5]	43.4 [42.6, 44.2]	0.971 [0.947, 0.996]
Maldives	89.7 [84.6, 93.2]	87.1 [81.9, 91.0]	0.971 [0.905, 1.042]
Nepal	46.6 [42.2, 51.1]	46.9 [41.8, 52.1]	1.007 [0.887, 1.142]
Pakistan	43.6 [39.2, 48.1]	45.5 [41.3, 49.8]	1.044 [0.932, 1.171]
West and Central Africa	57.7 [56.4, 59.0]	57.3 [55.9, 58.6]	0.993 [0.970, 1.016]
Benin	51.5 [48.5, 54.4]	55.1 [52.2, 57.9]	1.070 [0.999, 1.147]
Burkina Faso	35.1 [32.3, 38.1]	33.8 [31.1, 36.5]	0.961 [0.878, 1.052]
Cameroon	76.0 [72.1, 79.5]	76.7 [73.3, 79.8]	1.009 [0.956, 1.066]
Chad	45.8 [42.5, 49.1]	45.3 [42.0, 48.6]	0.990 [0.920, 1.065]
Congo	76.6 [72.3, 80.4]	76.6 [72.7, 80.1]	1.000 [0.936, 1.069]
Congo Democratic Republic	81.8 [79.4, 84.0]	81.6 [79.3, 83.8]	0.998 [0.965, 1.033]
Cote D'Ivoire	60.6 [56.4, 64.7]	59.0 [54.6, 63.2]	0.973 [0.888, 1.066]
Gabon	68.2 [63.5, 72.5]	69.9 [64.4, 74.9]	1.026 [0.929, 1.132]
Gambia	47.6 [43.2, 52.0]	47.5 [42.8, 52.2]	0.998 [0.890, 1.119]
Ghana	66.5 [61.8, 70.9]	67.8 [63.2, 72.1]	1.020 [0.940, 1.106]
Guinea	29.1 [24.8, 33.8]	25.5 [21.9, 29.4]	0.876 [0.735, 1.044]
Liberia	58.2 [54.2, 62.1]	58.0 [54.5, 61.5]	0.997 [0.911, 1.091]
Mali	54.1 [50.3, 57.9]	55.2 [51.4, 58.9]	1.020 [0.940, 1.107]
Niger	38.9 [35.6, 42.2]	35.2 [31.9, 38.6]	0.906 [0.820, 1.001]
Nigeria	51.7 [49.4, 54.0]	51.6 [49.3, 53.9]	0.999 [0.951, 1.049]
Senegal	62.3 [58.8, 65.6]	63.2 [59.9, 66.4]	1.015 [0.949, 1.087]
Sierra Leone	46.8 [43.0, 50.7]	45.4 [41.5, 49.3]	0.968 [0.882, 1.063]
Togo	68.1 [64.3, 71.7]	69.0 [65.8, 72.1]	1.013 [0.947, 1.085]

Notes: 95% confidence intervals are shown in brackets.

Table S7. Percentage of children 6–23 months old receiving vitamin A supplementation by sex.

	Females	Males	Males/Females
	%	%	Relative difference
Pooled	59.0 [58.3, 59.7]	58.4 [57.7, 59.1]	0.989 [0.977, 1.002]

	Females	Males	Males/Females
	%	%	Relative difference
Low-Income Countries	60.3 [59.2, 61.4]	58.9 [57.7, 60.0]	0.977 [0.956, 0.998]
Lower-Middle-Income Countries	58.1 [57.1, 59.0]	57.9 [57.1, 58.8]	0.998 [0.982, 1.014]
Upper-Middle-Income Countries	72.9 [69.0, 76.5]	67.6 [63.0, 71.9]	0.928 [0.851, 1.011]
East Asia and Pacific	66.5 [64.1, 68.9]	63.5 [61.1, 65.9]	0.955 [0.908, 1.004]
Cambodia	68.9 [65.3, 72.2]	63.3 [59.7, 66.8]	0.920 [0.862, 0.981]
Indonesia	70.5 [67.6, 73.3]	67.1 [64.1, 70.0]	0.952 [0.898, 1.009]
Myanmar	49.0 [43.8, 54.1]	50.1 [44.6, 55.5]	1.023 [0.895, 1.169]
Papua New Guinea	41.3 [37.1, 45.7]	39.9 [35.1, 44.8]	0.965 [0.832, 1.119]
Timor-Leste	61.6 [57.4, 65.5]	62.0 [57.6, 66.3]	1.008 [0.927, 1.095]
Eastern and Southern Africa	62.5 [60.9, 64.1]	61.8 [60.1, 63.5]	0.989 [0.958, 1.021]
Burundi	80.3 [77.4, 82.9]	81.6 [78.6, 84.4]	1.017 [0.972, 1.065]
Comoros	54.0 [47.2, 60.6]	48.1 [42.0, 54.2]	0.891 [0.754, 1.053]
Ethiopia	53.2 [49.3, 57.2]	49.8 [45.7, 53.9]	0.936 [0.847, 1.034]
Kenya	76.0 [72.7, 79.1]	77.2 [73.9, 80.1]	1.015 [0.961, 1.072]
Lesotho	73.0 [68.1, 77.4]	73.6 [68.6, 78.0]	1.007 [0.926, 1.095]
Malawi	85.3 [83.5, 87.0]	83.3 [81.2, 85.2]	0.976 [0.948, 1.006]
Mozambique	69.0 [65.8, 72.1]	71.7 [68.5, 74.7]	1.039 [0.986, 1.095]
Namibia	88.2 [84.9, 90.9]	85.9 [82.3, 88.9]	0.974 [0.927, 1.024]
Rwanda	87.6 [85.2, 89.7]	88.0 [85.8, 90.0]	1.005 [0.974, 1.037]
South Africa	82.3 [77.1, 86.6]	74.6 [68.1, 80.1]	0.906 [0.819, 1.003]
Tanzania	42.9 [39.4, 46.4]	43.5 [40.1, 46.9]	1.013 [0.919, 1.117]
Uganda	59.3 [55.2, 63.3]	64.3 [60.3, 68.1]	1.084 [1.005, 1.169]
Zambia	69.6 [66.8, 72.3]	67.9 [65.3, 70.4]	0.975 [0.927, 1.025]
Europe and Central Asia	54.5 [50.5, 58.5]	54.3 [50.5, 58.1]	0.996 [0.913, 1.087]
Kyrgyz Republic	40.7 [35.3, 46.4]	46.9 [41.7, 52.1]	1.150 [0.981, 1.349]
Tajikistan	62.2 [57.1, 67.1]	58.7 [53.6, 63.6]	0.944 [0.852, 1.045]
Latin America and Caribbean	56.8 [54.7, 59.0]	59.3 [57.1, 61.4]	1.043 [0.995, 1.094]
Dominican Republic	33.0 [27.9, 38.5]	33.5 [28.5, 39.0]	1.016 [0.800, 1.291]
Guatemala	60.9 [57.6, 64.0]	61.3 [58.1, 64.3]	1.007 [0.942, 1.077]
Haiti	52.0 [47.2, 56.7]	57.7 [52.9, 62.4]	1.111 [1.003, 1.232]
Honduras	79.3 [76.5, 81.9]	80.9 [78.4, 83.2]	1.020 [0.978, 1.064]
Middle East and North Africa	28.4 [26.4, 30.4]	27.6 [25.9, 29.4]	0.973 [0.894, 1.058]
Egypt	20.5 [18.4, 22.7]	20.7 [18.8, 22.7]	1.012 [0.885, 1.158]
Jordan	51.6 [45.0, 58.2]	54.0 [46.7, 61.2]	1.046 [0.887, 1.233]
Yemen	53.3 [50.0, 56.5]	50.2 [47.2, 53.3]	0.943 [0.878, 1.012]
South Asia	61.5 [60.4, 62.5]	61.3 [60.4, 62.2]	0.997 [0.979, 1.015]
Afghanistan	49.4 [43.8, 55.0]	49.7 [44.0, 55.4]	1.006 [0.938, 1.078]
Bangladesh	54.4 [50.8, 57.8]	49.5 [45.6, 53.5]	0.911 [0.835, 0.994]
India	60.4 [59.6, 61.3]	60.8 [60.0, 61.6]	1.006 [0.989, 1.024]
Maldives	53.4 [46.8, 59.9]	56.5 [48.8, 63.8]	1.057 [0.891, 1.254]
Nepal	72.6 [68.3, 76.6]	79.3 [75.3, 82.9]	1.093 [1.023, 1.167]
Pakistan	72.3 [67.8, 76.4]	71.5 [67.6, 75.1]	0.989 [0.928, 1.053]
West and Central Africa	55.5 [53.8, 57.1]	54.7 [53.1, 56.3]	0.986 [0.962, 1.011]
Benin	51.0 [47.9, 54.0]	51.3 [48.3, 54.3]	1.007 [0.939, 1.079]
Burkina Faso	66.2 [63.1, 69.2]	66.0 [62.8, 69.1]	0.997 [0.950, 1.046]
Cameroon	77.3 [73.7, 80.4]	78.2 [74.6, 81.5]	1.012 [0.966, 1.061]
Chad	43.1 [39.7, 46.6]	42.6 [39.3, 46.0]	0.988 [0.902, 1.082]
Congo	65.3 [59.9, 70.2]	65.1 [60.3, 69.7]	0.997 [0.914, 1.088]
Congo Democratic Republic	70.7 [67.5, 73.8]	66.3 [62.6, 69.8]	0.937 [0.888, 0.988]
Cote D'Ivoire	61.3 [56.6, 65.8]	61.9 [57.1, 66.5]	1.010 [0.928, 1.099]
Gabon	58.5 [52.8, 63.9]	55.1 [49.6, 60.5]	0.943 [0.818, 1.087]
Gambia	73.4 [68.7, 77.6]	76.5 [72.8, 79.9]	1.043 [0.977, 1.113]
Ghana	72.7 [66.9, 77.8]	68.3 [63.0, 73.2]	0.939 [0.870, 1.013]
Guinea	38.6 [33.4, 44.1]	40.5 [35.8, 45.4]	1.049 [0.908, 1.211]
Liberia	64.6 [59.8, 69.2]	60.9 [55.6, 66.0]	0.942 [0.850, 1.045]
Mali	59.5 [55.8, 63.2]	57.0 [52.9, 61.0]	0.958 [0.885, 1.036]

	Females	Males	Males/Females
	%	%	Relative difference
Niger	62.7 [59.2, 66.2]	60.0 [55.8, 64.1]	0.956 [0.901, 1.015]
Nigeria	40.3 [37.4, 43.2]	41.6 [38.8, 44.4]	1.032 [0.973, 1.095]
Senegal	77.8 [74.5, 80.8]	77.4 [74.3, 80.2]	0.994 [0.947, 1.043]
Sierra Leone	85.1 [82.0, 87.7]	84.0 [80.9, 86.7]	0.987 [0.949, 1.027]
Togo	81.7 [78.4, 84.5]	81.1 [77.6, 84.2]	0.993 [0.947, 1.042]

Notes: 95% confidence intervals are shown in brackets.

Table S8. Percentage of children 6–23 months old receiving either vitamin-A-rich foods or vitamin A supplementation by sex.

	Females	Males	Males/Females
	%	%	Relative difference
Pooled	78.5 [78.0, 79.0]	78.1 [77.6, 78.6]	0.995 [0.987, 1.002]
Low-Income Countries	80.4 [79.5, 81.3]	79.8 [78.7, 80.7]	0.992 [0.979, 1.005]
Lower-Middle-Income Countries	77.3 [76.6, 77.9]	77.1 [76.4, 77.7]	0.998 [0.988, 1.007]
Upper-Middle-Income Countries	92.0 [89.8, 93.8]	88.9 [85.6, 91.4]	0.966 [0.929, 1.004]
East Asia and Pacific	91.2 [89.9, 92.3]	89.1 [87.5, 90.6]	0.978 [0.957, 0.999]
Cambodia	93.5 [91.5, 95.1]	91.7 [89.5, 93.4]	0.980 [0.956, 1.005]
Indonesia	92.9 [91.3, 94.1]	90.4 [88.5, 92.1]	0.974 [0.950, 0.998]
Myanmar	81.3 [76.8, 85.1]	80.9 [76.3, 84.7]	0.994 [0.931, 1.062]
Papua New Guinea	89.4 [86.2, 91.9]	92.0 [89.9, 93.8]	1.030 [0.996, 1.065]
Timor-Leste	84.8 [81.6, 87.5]	85.7 [82.7, 88.3]	1.011 [0.970, 1.055]
Eastern and Southern Africa	81.4 [79.9, 82.8]	80.8 [79.1, 82.4]	0.992 [0.971, 1.014]
Burundi	96.0 [94.5, 97.1]	96.0 [94.4, 97.1]	1.000 [0.981, 1.018]
Comoros	86.5 [82.0, 90.0]	81.1 [75.8, 85.5]	0.938 [0.872, 1.009]
Ethiopia	63.1 [59.4, 66.8]	60.6 [56.4, 64.8]	0.960 [0.886, 1.041]
Kenya	90.4 [88.0, 92.3]	91.6 [89.5, 93.2]	1.013 [0.985, 1.042]
Lesotho	86.7 [82.8, 89.8]	88.2 [84.7, 90.9]	1.017 [0.968, 1.069]
Malawi	94.5 [93.3, 95.5]	93.9 [92.4, 95.1]	0.994 [0.976, 1.012]
Mozambique	88.4 [86.3, 90.1]	89.2 [87.0, 91.0]	1.009 [0.981, 1.039]
Namibia	93.9 [91.1, 95.8]	95.5 [93.5, 96.9]	1.018 [0.989, 1.047]
Rwanda	94.5 [92.9, 95.7]	93.9 [92.2, 95.3]	0.994 [0.974, 1.015]
South Africa	94.3 [91.2, 96.4]	90.0 [85.5, 93.2]	0.954 [0.908, 1.003]
Tanzania	82.5 [79.9, 84.8]	82.8 [80.2, 85.1]	1.004 [0.966, 1.044]
Uganda	82.2 [78.6, 85.3]	84.9 [81.5, 87.7]	1.033 [0.984, 1.084]
Zambia	89.2 [87.3, 90.9]	88.5 [86.6, 90.1]	0.991 [0.964, 1.019]
Europe and Central Asia	80.3 [77.1, 83.1]	77.6 [74.5, 80.5]	0.967 [0.924, 1.012]
Kyrgyz Republic	81.9 [77.3, 85.7]	80.5 [75.8, 84.4]	0.983 [0.920, 1.050]
Tajikistan	79.4 [75.1, 83.1]	75.9 [71.6, 79.8]	0.957 [0.899, 1.017]
Latin America and Caribbean	87.7 [86.3, 89.0]	88.4 [87.0, 89.6]	1.008 [0.988, 1.028]
Dominican Republic	82.9 [78.6, 86.5]	81.4 [77.4, 84.8]	0.981 [0.922, 1.045]
Guatemala	90.7 [89.0, 92.2]	90.1 [88.3, 91.6]	0.993 [0.970, 1.017]
Haiti	80.4 [76.5, 83.8]	84.6 [80.8, 87.9]	1.053 [0.996, 1.113]
Honduras	95.7 [94.3, 96.8]	95.9 [94.6, 96.8]	1.002 [0.985, 1.018]
Middle East and North Africa	69.4 [67.6, 71.3]	69.2 [67.3, 70.9]	0.996 [0.962, 1.031]
Egypt	68.4 [66.1, 70.7]	68.1 [65.8, 70.3]	0.995 [0.952, 1.041]
Jordan	88.0 [82.6, 91.8]	90.4 [85.9, 93.6]	1.028 [0.965, 1.095]
Yemen	71.4 [68.6, 74.0]	71.2 [68.6, 73.6]	0.997 [0.953, 1.044]
South Asia	76.4 [75.7, 77.2]	76.1 [75.4, 76.9]	0.996 [0.984, 1.008]
Afghanistan	72.9 [69.3, 76.2]	71.0 [67.4, 74.5]	0.975 [0.937, 1.015]
Bangladesh	79.9 [77.0, 82.5]	78.5 [75.4, 81.4]	0.983 [0.938, 1.030]
India	74.7 [74.0, 75.4]	74.4 [73.7, 75.1]	0.996 [0.984, 1.008]
Maldives	93.8 [89.1, 96.6]	92.6 [88.1, 95.5]	0.987 [0.936, 1.042]
Nepal	80.7 [76.6, 84.3]	85.7 [82.2, 88.6]	1.062 [1.008, 1.120]
Pakistan	83.2 [79.7, 86.3]	83.7 [80.2, 86.7]	1.006 [0.957, 1.057]
West and Central Africa	77.2 [75.9, 78.4]	77.4 [76.2, 78.6]	1.003 [0.988, 1.017]

	Females	Males	Males/Females
	%	%	Relative difference
Benin	72.4 [69.6, 75.0]	73.7 [71.2, 76.0]	1.018 [0.974, 1.064]
Burkina Faso	76.2 [73.3, 78.8]	74.9 [72.0, 77.5]	0.983 [0.946, 1.022]
Cameroon	95.4 [93.5, 96.7]	95.2 [92.7, 96.9]	0.998 [0.973, 1.024]
Chad	65.7 [62.3, 68.8]	66.1 [62.9, 69.0]	1.006 [0.953, 1.062]
Congo	92.8 [89.7, 95.0]	89.1 [85.2, 92.0]	0.960 [0.916, 1.006]
Congo Democratic Republic	93.2 [91.6, 94.5]	92.8 [91.2, 94.1]	0.996 [0.979, 1.013]
Cote D'Ivoire	81.9 [78.3, 85.0]	81.2 [77.6, 84.4]	0.992 [0.944, 1.042]
Gabon	85.0 [81.0, 88.3]	84.3 [79.7, 88.0]	0.991 [0.929, 1.058]
Gambia	84.7 [81.5, 87.4]	86.5 [83.6, 89.0]	1.022 [0.980, 1.065]
Ghana	87.7 [83.7, 90.9]	85.8 [82.0, 89.0]	0.978 [0.934, 1.025]
Guinea	52.2 [47.0, 57.3]	53.4 [48.4, 58.3]	1.024 [0.915, 1.145]
Liberia	82.6 [78.7, 85.9]	82.7 [78.9, 86.0]	1.002 [0.944, 1.064]
Mali	78.9 [75.9, 81.6]	77.1 [73.9, 80.0]	0.977 [0.932, 1.025]
Niger	75.6 [72.4, 78.5]	71.7 [67.9, 75.2]	0.948 [0.904, 0.994]
Nigeria	67.5 [65.1, 69.8]	69.4 [67.1, 71.7]	1.029 [0.996, 1.063]
Senegal	89.4 [87.3, 91.2]	90.7 [88.9, 92.3]	1.015 [0.988, 1.042]
Sierra Leone	89.9 [87.4, 91.9]	89.2 [86.7, 91.4]	0.993 [0.964, 1.023]
Togo	92.8 [90.5, 94.6]	92.3 [90.4, 93.9]	0.994 [0.966, 1.024]

Notes: 95% confidence intervals are shown in brackets.

Table S9. Percentage of children 6–23 months old receiving minimum dietary diversity by living standards.

	Wealthiest	Poorest	Poorest/Wealthiest
	%	%	Relative difference
Pooled	31.4 [30.4, 32.5]	15.5 [14.9, 16.1]	0.493 [0.468, 0.520]
Low-Income Countries	29.9 [28.3, 31.5]	11.7 [10.8, 12.6]	0.390 [0.355, 0.429]
Lower-Middle-Income Countries	31.6 [30.3, 33.1]	16.6 [15.8, 17.4]	0.523 [0.490, 0.559]
Upper-Middle-Income Countries	52.0 [41.3, 62.4]	34.4 [28.9, 40.3]	0.662 [0.508, 0.862]
East Asia and Pacific	63.0 [58.6, 67.1]	31.5 [28.9, 34.2]	0.500 [0.449, 0.557]
Cambodia	34.2 [28.1, 40.9]	25.1 [20.8, 30.0]	0.733 [0.563, 0.954]
Indonesia	70.4 [65.3, 75.1]	36.0 [32.7, 39.4]	0.511 [0.455, 0.574]
Myanmar	33.9 [25.2, 43.9]	16.6 [12.1, 22.4]	0.490 [0.325, 0.737]
Papua New Guinea	47.1 [38.0, 56.4]	28.1 [22.0, 35.2]	0.597 [0.440, 0.811]
Timor-Leste	44.5 [34.6, 54.8]	16.8 [12.8, 21.8]	0.378 [0.267, 0.536]
Eastern and Southern Africa	31.8 [29.3, 34.3]	14.0 [12.5, 15.6]	0.439 [0.384, 0.503]
Burundi	27.7 [23.2, 32.7]	11.6 [8.6, 15.6]	0.420 [0.295, 0.600]
Comoros	23.5 [14.3, 36.1]	16.9 [11.0, 25.3]	0.720 [0.388, 1.336]
Ethiopia	11.3 [8.0, 15.7]	1.3 [0.6, 3.1]	0.119 [0.049, 0.288]
Kenya	57.9 [50.6, 64.8]	18.3 [15.2, 21.9]	0.316 [0.254, 0.394]
Lesotho	37.2 [27.3, 48.4]	8.0 [4.6, 13.6]	0.216 [0.117, 0.396]
Malawi	40.0 [35.2, 44.9]	20.5 [17.9, 23.4]	0.513 [0.427, 0.615]
Mozambique	29.0 [23.7, 34.9]	30.9 [25.7, 36.6]	1.065 [0.821, 1.383]
Namibia	54.3 [45.5, 62.9]	9.2 [5.7, 14.5]	0.170 [0.104, 0.277]
Rwanda	46.5 [41.5, 51.7]	17.1 [13.9, 20.8]	0.367 [0.292, 0.460]
South Africa	50.3 [36.3, 64.4]	35.9 [28.2, 44.3]	0.712 [0.497, 1.020]
Tanzania	39.4 [34.0, 45.1]	12.3 [9.7, 15.4]	0.312 [0.238, 0.408]
Uganda	31.1 [25.8, 37.0]	8.9 [6.6, 11.9]	0.286 [0.202, 0.405]
Zambia	32.4 [26.9, 38.5]	13.8 [11.1, 17.0]	0.425 [0.322, 0.561]
Europe and Central Asia	37.2 [32.1, 42.5]	32.8 [27.4, 38.6]	0.882 [0.707, 1.100]
Kyrgyz Republic	32.2 [24.4, 41.2]	38.3 [29.8, 47.6]	1.189 [0.841, 1.682]
Tajikistan	40.0 [33.7, 46.7]	29.7 [23.0, 37.4]	0.742 [0.555, 0.993]
Latin America and Caribbean	60.2 [56.0, 64.3]	38.0 [35.4, 40.6]	0.631 [0.573, 0.695]
Dominican Republic	62.1 [49.4, 73.3]	36.7 [30.9, 42.9]	0.591 [0.463, 0.755]
Guatemala	71.8 [66.2, 76.8]	45.6 [41.5, 49.8]	0.635 [0.565, 0.715]
Haiti	27.8 [20.7, 36.2]	16.9 [13.0, 21.6]	0.608 [0.418, 0.885]
Honduras	69.1 [62.3, 75.1]	48.8 [45.0, 52.5]	0.706 [0.626, 0.797]

	Wealthiest	Poorest	Poorest/Wealthiest
	%	%	Relative difference
Middle East and North Africa	36.4 [33.4, 39.6]	30.3 [27.0, 33.8]	0.832 [0.722, 0.958]
Egypt	35.5 [31.9, 39.3]	37.3 [32.9, 41.9]	1.050 [0.894, 1.232]
Jordan	63.4 [43.0, 79.9]	33.6 [24.8, 43.7]	0.530 [0.353, 0.796]
Yemen	37.8 [32.9, 43.0]	12.1 [8.9, 16.3]	0.320 [0.230, 0.445]
South Asia	25.6 [24.1, 27.0]	14.2 [13.4, 15.1]	0.556 [0.512, 0.604]
Afghanistan	30.9 [23.5, 39.6]	12.0 [9.3, 15.2]	0.386 [0.273, 0.547]
Bangladesh	34.9 [29.8, 40.2]	12.2 [9.3, 15.8]	0.350 [0.260, 0.472]
India	22.8 [21.3, 24.4]	15.1 [14.3, 15.9]	0.660 [0.606, 0.719]
Maldives	67.2 [50.7, 80.3]	61.6 [53.7, 69.0]	0.918 [0.718, 1.174]
Nepal	46.8 [37.9, 55.8]	14.2 [10.6, 18.9]	0.304 [0.216, 0.428]
Pakistan	28.3 [23.2, 33.9]	10.9 [7.5, 15.6]	0.387 [0.257, 0.583]
West and Central Africa	26.5 [24.5, 28.6]	8.7 [7.7, 9.8]	0.328 [0.283, 0.379]
Benin	43.0 [38.4, 47.8]	19.7 [16.6, 23.2]	0.457 [0.374, 0.559]
Burkina Faso	12.7 [9.6, 16.5]	3.7 [2.4, 5.6]	0.289 [0.175, 0.476]
Cameroon	45.1 [38.8, 51.5]	8.2 [5.7, 11.8]	0.183 [0.124, 0.269]
Chad	20.1 [16.2, 24.6]	4.2 [2.7, 6.6]	0.211 [0.130, 0.345]
Congo	19.0 [13.0, 26.8]	11.2 [8.7, 14.4]	0.593 [0.384, 0.916]
Congo Democratic Republic	28.2 [23.0, 34.0]	10.7 [7.7, 14.8]	0.381 [0.261, 0.555]
Cote D'Ivoire	15.5 [10.8, 21.8]	2.4 [1.2, 4.6]	0.151 [0.071, 0.325]
Gabon	22.3 [13.5, 34.3]	11.7 [7.6, 17.5]	0.525 [0.282, 0.979]
Gambia	14.1 [9.2, 21.0]	7.2 [4.7, 10.8]	0.510 [0.286, 0.909]
Ghana	42.4 [33.1, 52.4]	15.8 [11.8, 20.9]	0.372 [0.259, 0.536]
Guinea	16.1 [10.8, 23.2]	2.9 [1.5, 5.5]	0.179 [0.084, 0.380]
Liberia	10.0 [5.5, 17.6]	7.1 [4.7, 10.6]	0.710 [0.351, 1.439]
Mali	30.4 [25.8, 35.4]	12.4 [8.6, 17.6]	0.408 [0.277, 0.602]
Niger	21.7 [18.3, 25.5]	3.6 [2.2, 5.9]	0.165 [0.098, 0.279]
Nigeria	24.6 [21.0, 28.6]	8.0 [6.3, 10.1]	0.324 [0.244, 0.431]
Senegal	44.7 [37.2, 52.4]	12.8 [10.3, 15.9]	0.287 [0.218, 0.378]
Sierra Leone	25.8 [18.9, 34.2]	8.7 [6.5, 11.7]	0.338 [0.223, 0.514]
Togo	29.9 [24.5, 35.9]	17.2 [13.7, 21.4]	0.576 [0.430, 0.771]

Notes: 95% confidence intervals are shown in brackets.

Table S10. Percentage of children 6–23 months old receiving vitamin-A-rich foods by living standards.

	Wealthiest	Poorest	Poorest/Wealthiest
	%	%	Relative difference
Pooled	61.5 [60.4, 62.6]	49.6 [48.6, 50.6]	0.807 [0.786, 0.829]
Low-Income Countries	65.4 [63.6, 67.1]	53.7 [51.8, 55.5]	0.820 [0.786, 0.856]
Lower-Middle-Income Countries	59.2 [57.9, 60.6]	47.4 [46.2, 48.5]	0.799 [0.773, 0.827]
Upper-Middle-Income Countries	81.0 [72.5, 87.3]	66.9 [61.2, 72.1]	0.826 [0.731, 0.933]
East Asia and Pacific	87.0 [83.6, 89.8]	77.5 [75.0, 79.8]	0.891 [0.850, 0.934]
Cambodia	80.0 [73.7, 85.2]	83.4 [79.2, 87.0]	1.042 [0.958, 1.134]
Indonesia	88.2 [83.9, 91.4]	80.4 [77.5, 83.1]	0.912 [0.864, 0.964]
Myanmar	82.2 [73.7, 88.3]	62.3 [55.6, 68.4]	0.758 [0.663, 0.866]
Papua New Guinea	91.9 [88.3, 94.4]	84.8 [79.7, 88.8]	0.923 [0.868, 0.981]
Timor-Leste	73.3 [66.2, 79.4]	64.9 [58.7, 70.6]	0.885 [0.779, 1.005]
Eastern and Southern Africa	67.3 [64.3, 70.1]	54.6 [51.7, 57.4]	0.811 [0.759, 0.867]
Burundi	81.7 [77.6, 85.2]	84.9 [80.8, 88.3]	1.040 [0.974, 1.110]
Comoros	74.2 [61.9, 83.6]	70.2 [60.8, 78.2]	0.947 [0.791, 1.133]
Ethiopia	39.8 [33.8, 46.2]	18.2 [14.4, 22.6]	0.456 [0.348, 0.597]
Kenya	86.4 [81.3, 90.3]	52.0 [47.3, 56.7]	0.601 [0.542, 0.667]
Lesotho	66.1 [52.8, 77.3]	55.1 [48.5, 61.5]	0.833 [0.669, 1.037]
Malawi	78.4 [73.3, 82.8]	69.8 [66.4, 72.9]	0.890 [0.825, 0.959]
Mozambique	69.1 [65.0, 72.9]	75.4 [70.7, 79.6]	1.092 [1.007, 1.185]
Namibia	84.7 [76.6, 90.3]	66.4 [58.9, 73.1]	0.784 [0.686, 0.895]
Rwanda	82.9 [78.5, 86.5]	67.0 [63.1, 70.8]	0.809 [0.751, 0.871]

	Wealthiest	Poorest	Poorest/Wealthiest
	%	%	Relative difference
South Africa	82.2 [70.0, 90.1]	66.0 [57.7, 73.4]	0.803 [0.679, 0.949]
Tanzania	82.0 [77.9, 85.4]	66.7 [60.9, 72.0]	0.814 [0.740, 0.894]
Uganda	63.1 [57.2, 68.6]	65.1 [59.4, 70.4]	1.032 [0.914, 1.164]
Zambia	78.3 [73.3, 82.6]	72.7 [69.0, 76.2]	0.928 [0.859, 1.003]
Europe and Central Asia	58.7 [52.8, 64.3]	51.4 [45.3, 57.4]	0.876 [0.752, 1.020]
Kyrgyz Republic	65.1 [54.7, 74.2]	64.7 [55.2, 73.1]	0.993 [0.812, 1.216]
Tajikistan	55.0 [48.2, 61.5]	44.1 [36.6, 51.9]	0.802 [0.651, 0.989]
Latin America and Caribbean	81.1 [78.0, 83.9]	70.7 [68.4, 72.9]	0.871 [0.830, 0.914]
Dominican Republic	77.8 [67.7, 85.5]	68.2 [63.0, 72.9]	0.876 [0.769, 0.998]
Guatemala	89.0 [85.2, 92.0]	72.1 [68.5, 75.4]	0.810 [0.762, 0.861]
Haiti	63.0 [55.1, 70.3]	64.8 [58.4, 70.7]	1.028 [0.883, 1.197]
Honduras	86.9 [81.4, 90.9]	78.1 [74.9, 81.1]	0.899 [0.841, 0.961]
Middle East and North Africa	64.3 [61.1, 67.4]	53.2 [49.9, 56.5]	0.828 [0.765, 0.896]
Egypt	63.3 [59.3, 67.1]	59.9 [55.9, 63.9]	0.947 [0.865, 1.037]
Jordan	77.1 [59.3, 88.6]	69.1 [61.5, 75.8]	0.897 [0.724, 1.111]
Yemen	67.1 [62.1, 71.8]	34.6 [29.2, 40.3]	0.515 [0.432, 0.614]
South Asia	51.3 [49.6, 53.0]	41.8 [40.4, 43.1]	0.814 [0.778, 0.852]
Afghanistan	53.2 [46.7, 59.5]	43.0 [38.3, 47.7]	0.808 [0.696, 0.939]
Bangladesh	70.2 [64.9, 75.0]	53.2 [48.2, 58.1]	0.758 [0.675, 0.850]
India	46.5 [44.8, 48.2]	41.6 [40.6, 42.7]	0.895 [0.856, 0.936]
Maldives	88.1 [73.8, 95.1]	86.2 [80.1, 90.6]	0.978 [0.864, 1.107]
Nepal	52.7 [43.8, 61.4]	47.3 [41.4, 53.2]	0.897 [0.732, 1.101]
Pakistan	60.6 [53.7, 67.0]	33.4 [26.8, 40.7]	0.551 [0.436, 0.696]
West and Central Africa	65.2 [63.2, 67.1]	50.8 [48.7, 52.8]	0.779 [0.740, 0.819]
Benin	63.4 [58.5, 67.9]	43.9 [39.9, 48.0]	0.693 [0.617, 0.779]
Burkina Faso	50.4 [45.5, 55.4]	31.2 [27.0, 35.8]	0.619 [0.522, 0.735]
Cameroon	83.2 [77.9, 87.5]	67.1 [61.4, 72.3]	0.806 [0.730, 0.890]
Chad	56.9 [51.9, 61.7]	42.7 [37.7, 47.8]	0.750 [0.649, 0.867]
Congo	72.2 [64.3, 79.0]	81.6 [78.9, 84.1]	1.130 [1.017, 1.255]
Congo Democratic Republic	80.5 [76.8, 83.7]	79.9 [75.9, 83.4]	0.993 [0.932, 1.058]
Cote D'Ivoire	61.9 [54.3, 69.0]	55.5 [49.8, 61.0]	0.896 [0.768, 1.046]
Gabon	64.6 [52.8, 74.8]	73.2 [68.8, 77.2]	1.133 [0.948, 1.355]
Gambia	48.8 [40.6, 57.1]	46.0 [39.8, 52.4]	0.943 [0.760, 1.169]
Ghana	68.6 [58.7, 77.0]	61.6 [57.3, 65.7]	0.897 [0.773, 1.042]
Guinea	45.2 [36.1, 54.6]	15.6 [12.0, 20.1]	0.346 [0.249, 0.480]
Liberia	50.8 [42.7, 58.9]	57.4 [52.3, 62.3]	1.130 [0.946, 1.351]
Mali	63.7 [57.7, 69.3]	49.8 [43.9, 55.7]	0.781 [0.673, 0.906]
Niger	54.6 [50.2, 58.9]	24.3 [19.5, 29.9]	0.446 [0.356, 0.559]
Nigeria	62.3 [58.5, 65.9]	41.7 [38.4, 45.1]	0.670 [0.606, 0.740]
Senegal	76.9 [70.2, 82.5]	49.6 [45.1, 54.1]	0.644 [0.572, 0.726]
Sierra Leone	53.1 [45.4, 60.7]	41.8 [36.7, 47.1]	0.787 [0.651, 0.951]
Togo	70.8 [64.4, 76.4]	68.5 [63.5, 73.2]	0.968 [0.868, 1.080]

Notes: 95% confidence intervals are shown in brackets.

Table S11. Percentage of children 6–23 months old receiving vitamin A supplementation by living standards.

	Wealthiest	Poorest	Poorest/Wealthiest
	%	%	Relative difference
Pooled	65.4 [64.3, 66.4]	51.3 [50.2, 52.3]	0.784 [0.764, 0.805]
Low-Income Countries	66.8 [64.9, 68.7]	54.4 [52.7, 56.0]	0.814 [0.781, 0.848]
Lower-Middle-Income Countries	64.5 [63.2, 65.8]	49.4 [48.1, 50.7]	0.766 [0.741, 0.791]
Upper-Middle-Income Countries	72.7 [64.0, 80.0]	67.7 [61.6, 73.3]	0.931 [0.809, 1.071]
East Asia and Pacific	68.8 [64.8, 72.5]	57.6 [54.6, 60.5]	0.837 [0.777, 0.903]
Cambodia	62.1 [56.1, 67.8]	61.5 [56.0, 66.7]	0.989 [0.873, 1.121]
Indonesia	72.9 [68.2, 77.2]	61.0 [57.3, 64.6]	0.836 [0.768, 0.911]
Myanmar	48.2 [38.1, 58.4]	47.9 [41.1, 54.7]	0.994 [0.771, 1.280]

	Wealthiest	Poorest	Poorest/Wealthiest
	%	%	Relative difference
Papua New Guinea	59.7 [49.9, 68.8]	24.9 [18.8, 32.3]	0.417 [0.305, 0.572]
Timor-Leste	70.2 [63.1, 76.4]	54.0 [47.2, 60.7]	0.770 [0.657, 0.902]
Eastern and Southern Africa	68.5 [65.7, 71.1]	56.6 [54.2, 59.0]	0.827 [0.781, 0.875]
Burundi	84.8 [80.4, 88.4]	79.3 [74.9, 83.1]	0.935 [0.873, 1.001]
Comoros	52.6 [41.1, 63.9]	43.4 [34.9, 52.4]	0.825 [0.615, 1.107]
Ethiopia	55.2 [48.5, 61.6]	46.2 [41.2, 51.3]	0.838 [0.717, 0.980]
Kenya	79.1 [72.3, 84.5]	65.3 [60.1, 70.2]	0.826 [0.741, 0.921]
Lesotho	87.9 [81.2, 92.4]	68.5 [60.6, 75.4]	0.779 [0.688, 0.881]
Malawi	83.7 [79.6, 87.0]	82.8 [79.7, 85.5]	0.989 [0.937, 1.045]
Mozambique	87.6 [84.2, 90.3]	60.1 [54.6, 65.3]	0.686 [0.624, 0.754]
Namibia	84.2 [75.8, 90.1]	86.3 [81.1, 90.2]	1.024 [0.929, 1.129]
Rwanda	89.5 [85.4, 92.5]	87.1 [83.8, 89.8]	0.973 [0.924, 1.025]
South Africa	80.7 [68.1, 89.1]	79.6 [71.1, 86.1]	0.987 [0.844, 1.155]
Tanzania	56.6 [51.1, 61.9]	31.5 [26.7, 36.7]	0.557 [0.463, 0.671]
Uganda	67.9 [62.4, 73.0]	63.3 [56.7, 69.4]	0.931 [0.820, 1.058]
Zambia	74.5 [69.5, 78.9]	62.9 [59.1, 66.6]	0.844 [0.775, 0.920]
Europe and Central Asia	54.3 [48.6, 59.9]	51.8 [45.5, 58.1]	0.955 [0.814, 1.120]
Kyrgyz Republic	52.7 [42.6, 62.6]	43.8 [35.5, 52.4]	0.831 [0.635, 1.088]
Tajikistan	55.2 [48.3, 61.9]	56.3 [47.5, 64.7]	1.019 [0.838, 1.239]
Latin America and Caribbean	61.1 [56.9, 65.2]	52.7 [49.8, 55.5]	0.862 [0.791, 0.940]
Dominican Republic	34.8 [22.2, 49.9]	28.9 [24.0, 34.4]	0.832 [0.533, 1.299]
Guatemala	59.6 [53.5, 65.3]	56.7 [51.9, 61.3]	0.951 [0.836, 1.083]
Haiti	63.9 [55.4, 71.6]	49.4 [43.1, 55.7]	0.773 [0.647, 0.925]
Honduras	82.9 [77.4, 87.2]	80.3 [77.0, 83.1]	0.969 [0.903, 1.039]
Middle East and North Africa	30.1 [26.9, 33.6]	26.6 [23.6, 29.8]	0.882 [0.751, 1.037]
Egypt	22.0 [18.8, 25.6]	18.1 [14.9, 21.7]	0.821 [0.644, 1.048]
Jordan	63.0 [44.9, 78.1]	45.6 [36.7, 54.8]	0.724 [0.519, 1.009]
Yemen	57.2 [50.9, 63.3]	46.6 [41.8, 51.4]	0.814 [0.702, 0.945]
South Asia	64.2 [62.5, 65.8]	54.5 [53.2, 55.7]	0.849 [0.820, 0.878]
Afghanistan	54.5 [46.5, 62.3]	48.8 [43.2, 54.5]	0.895 [0.757, 1.058]
Bangladesh	53.1 [46.7, 59.3]	51.5 [45.7, 57.3]	0.972 [0.823, 1.146]
India	65.0 [63.4, 66.5]	53.0 [51.9, 54.1]	0.816 [0.791, 0.843]
Maldives	48.8 [33.6, 64.2]	55.6 [47.6, 63.4]	1.140 [0.815, 1.594]
Nepal	73.4 [65.5, 80.1]	76.1 [70.3, 81.1]	1.037 [0.920, 1.169]
Pakistan	69.7 [63.1, 75.6]	62.8 [56.8, 68.4]	0.901 [0.793, 1.024]
West and Central Africa	72.5 [70.5, 74.5]	41.9 [39.4, 44.5]	0.578 [0.540, 0.618]
Benin	63.1 [57.8, 68.1]	37.8 [33.6, 42.1]	0.598 [0.521, 0.688]
Burkina Faso	69.8 [64.8, 74.3]	57.3 [52.3, 62.2]	0.822 [0.736, 0.918]
Cameroon	73.5 [66.6, 79.4]	84.1 [79.5, 87.8]	1.144 [1.036, 1.263]
Chad	48.6 [42.9, 54.3]	43.8 [38.5, 49.3]	0.902 [0.763, 1.066]
Congo	77.4 [68.1, 84.6]	59.4 [54.1, 64.4]	0.767 [0.669, 0.879]
Congo Democratic Republic	86.2 [82.6, 89.2]	58.0 [52.6, 63.2]	0.672 [0.609, 0.742]
Cote D'Ivoire	77.4 [69.4, 83.8]	47.9 [42.0, 53.8]	0.619 [0.531, 0.721]
Gabon	53.9 [42.1, 65.2]	62.6 [56.4, 68.5]	1.163 [0.917, 1.474]
Gambia	70.4 [61.0, 78.3]	76.9 [71.0, 81.8]	1.092 [0.949, 1.257]
Ghana	71.8 [64.6, 78.1]	63.9 [56.1, 71.1]	0.890 [0.766, 1.034]
Guinea	61.9 [52.8, 70.3]	31.0 [24.9, 38.0]	0.501 [0.389, 0.645]
Liberia	74.5 [62.0, 83.9]	54.3 [48.1, 60.3]	0.729 [0.607, 0.875]
Mali	68.5 [62.8, 73.8]	54.5 [48.0, 60.8]	0.796 [0.691, 0.916]
Niger	75.2 [70.8, 79.2]	51.8 [46.3, 57.4]	0.689 [0.611, 0.777]
Nigeria	68.4 [64.3, 72.2]	20.1 [16.8, 23.7]	0.293 [0.245, 0.351]
Senegal	82.7 [75.0, 88.4]	67.5 [62.7, 71.9]	0.815 [0.734, 0.905]
Sierra Leone	87.3 [80.8, 91.8]	86.5 [82.4, 89.8]	0.991 [0.919, 1.068]
Togo	83.1 [76.9, 88.0]	84.1 [79.8, 87.6]	1.011 [0.934, 1.096]

Notes: 95% confidence intervals are shown in brackets.

Table S12. Percentage of children 6–23 months old receiving either vitamin-A-rich foods or vitamin A supplementation by living standards.

	Wealthiest	Poorest	Poorest/Wealthiest
	%	%	Relative difference
Pooled	83.8 [83.0, 84.6]	72.2 [71.4, 73.0]	0.861 [0.849, 0.874]
Low-Income Countries	85.6 [84.0, 87.0]	75.6 [74.1, 77.1]	0.884 [0.862, 0.907]
Lower-Middle-Income Countries	82.8 [81.8, 83.7]	70.2 [69.2, 71.2]	0.849 [0.833, 0.864]
Upper-Middle-Income Countries	95.6 [92.1, 97.6]	88.1 [84.4, 91.0]	0.921 [0.880, 0.965]
East Asia and Pacific	92.7 [90.0, 94.7]	86.6 [84.4, 88.4]	0.933 [0.902, 0.966]
Cambodia	91.0 [86.5, 94.1]	91.6 [87.9, 94.2]	1.007 [0.955, 1.062]
Indonesia	93.3 [89.8, 95.6]	88.5 [85.9, 90.7]	0.949 [0.911, 0.988]
Myanmar	89.3 [82.4, 93.8]	76.4 [70.8, 81.2]	0.855 [0.783, 0.934]
Papua New Guinea	95.9 [93.2, 97.5]	88.7 [84.0, 92.1]	0.925 [0.880, 0.972]
Timor-Leste	89.5 [84.9, 92.8]	81.8 [77.5, 85.3]	0.914 [0.857, 0.974]
Eastern and Southern Africa	86.3 [83.6, 88.6]	76.6 [74.3, 78.8]	0.888 [0.854, 0.924]
Burundi	96.7 [94.5, 98.1]	96.2 [93.9, 97.6]	0.994 [0.969, 1.020]
Comoros	82.7 [73.3, 89.3]	77.0 [67.8, 84.1]	0.930 [0.818, 1.058]
Ethiopia	67.7 [60.3, 74.2]	54.6 [49.4, 59.7]	0.807 [0.706, 0.922]
Kenya	94.7 [90.5, 97.1]	80.4 [76.0, 84.1]	0.849 [0.799, 0.901]
Lesotho	96.3 [92.0, 98.3]	83.2 [77.4, 87.7]	0.864 [0.807, 0.924]
Malawi	94.0 [91.1, 96.0]	93.2 [91.2, 94.8]	0.992 [0.962, 1.024]
Mozambique	94.4 [92.2, 95.9]	86.8 [82.8, 90.0]	0.920 [0.880, 0.962]
Namibia	96.2 [86.5, 99.0]	93.1 [89.5, 95.6]	0.968 [0.912, 1.029]
Rwanda	95.6 [93.2, 97.2]	93.2 [90.5, 95.2]	0.974 [0.945, 1.005]
South Africa	98.0 [91.0, 99.6]	91.0 [85.4, 94.6]	0.929 [0.877, 0.984]
Tanzania	90.5 [87.4, 92.9]	75.9 [71.2, 80.0]	0.838 [0.786, 0.894]
Uganda	89.1 [84.6, 92.4]	83.1 [77.7, 87.5]	0.933 [0.869, 1.002]
Zambia	92.3 [88.9, 94.7]	85.7 [82.5, 88.4]	0.929 [0.887, 0.972]
Europe and Central Asia	78.1 [73.2, 82.4]	76.6 [69.6, 82.4]	0.980 [0.886, 1.085]
Kyrgyz Republic	85.4 [77.7, 90.7]	79.5 [70.3, 86.4]	0.931 [0.823, 1.053]
Tajikistan	73.9 [67.6, 79.3]	75.0 [65.0, 82.8]	1.015 [0.881, 1.168]
Latin America and Caribbean	90.7 [88.2, 92.7]	84.4 [82.4, 86.2]	0.931 [0.901, 0.962]
Dominican Republic	84.5 [75.5, 90.6]	77.4 [72.7, 81.4]	0.916 [0.827, 1.014]
Guatemala	92.8 [89.7, 95.0]	86.5 [83.9, 88.7]	0.932 [0.896, 0.970]
Haiti	84.8 [77.3, 90.2]	80.5 [74.1, 85.6]	0.949 [0.856, 1.052]
Honduras	97.6 [94.7, 98.9]	94.2 [92.3, 95.6]	0.966 [0.941, 0.991]
Middle East and North Africa	72.9 [69.6, 75.9]	64.5 [61.4, 67.4]	0.885 [0.830, 0.942]
Egypt	69.8 [65.8, 73.5]	64.8 [60.9, 68.6]	0.929 [0.856, 1.007]
Jordan	98.3 [94.2, 99.5]	86.3 [79.7, 91.1]	0.879 [0.821, 0.940]
Yemen	82.3 [78.1, 85.8]	61.5 [56.7, 66.0]	0.747 [0.683, 0.817]
South Asia	79.9 [78.6, 81.1]	70.3 [69.3, 71.3]	0.880 [0.862, 0.899]
Afghanistan	77.8 [71.8, 82.8]	69.2 [64.7, 73.4]	0.890 [0.814, 0.973]
Bangladesh	83.5 [78.5, 87.4]	74.1 [69.2, 78.5]	0.888 [0.818, 0.964]
India	77.8 [76.5, 79.1]	68.9 [67.9, 69.8]	0.885 [0.866, 0.905]
Maldives	91.1 [77.4, 96.8]	90.4 [85.2, 93.9]	0.992 [0.895, 1.099]
Nepal	81.4 [73.4, 87.4]	85.1 [80.7, 88.6]	1.045 [0.951, 1.149]
Pakistan	87.9 [83.3, 91.4]	74.1 [68.6, 78.9]	0.843 [0.777, 0.916]
West and Central Africa	88.3 [87.0, 89.6]	68.0 [65.8, 70.1]	0.770 [0.744, 0.798]
Benin	83.8 [79.5, 87.4]	62.9 [58.4, 67.1]	0.750 [0.690, 0.815]
Burkina Faso	82.4 [78.0, 86.1]	66.6 [61.8, 71.1]	0.808 [0.742, 0.880]
Cameroon	96.0 [92.4, 97.9]	95.6 [93.0, 97.3]	0.996 [0.962, 1.031]
Chad	75.1 [70.9, 78.8]	64.0 [58.4, 69.3]	0.853 [0.772, 0.942]
Congo	90.2 [84.1, 94.2]	92.2 [90.0, 93.9]	1.022 [0.964, 1.083]
Congo Democratic Republic	97.2 [95.1, 98.4]	89.8 [86.6, 92.2]	0.924 [0.892, 0.956]
Cote D'Ivoire	89.4 [82.3, 93.9]	74.8 [69.6, 79.4]	0.837 [0.765, 0.916]
Gabon	81.9 [70.3, 89.6]	86.7 [83.3, 89.5]	1.059 [0.939, 1.194]
Gambia	84.3 [75.4, 90.3]	86.9 [83.0, 90.0]	1.032 [0.937, 1.135]

	Wealthiest	Poorest	Poorest/Wealthiest
	%	%	Relative difference
Ghana	88.7 [82.9, 92.8]	84.4 [79.2, 88.4]	0.951 [0.880, 1.026]
Guinea	75.1 [66.8, 81.9]	40.1 [33.2, 47.4]	0.534 [0.437, 0.654]
Liberia	86.7 [76.7, 92.8]	79.2 [74.5, 83.3]	0.914 [0.824, 1.014]
Mali	86.0 [82.5, 88.9]	72.8 [67.2, 77.8]	0.847 [0.781, 0.917]
Niger	88.4 [85.5, 90.7]	61.8 [56.1, 67.2]	0.699 [0.636, 0.768]
Nigeria	86.3 [83.4, 88.7]	52.8 [49.4, 56.3]	0.613 [0.570, 0.659]
Senegal	96.0 [91.5, 98.1]	82.9 [79.8, 85.7]	0.864 [0.824, 0.906]
Sierra Leone	91.2 [86.0, 94.6]	91.1 [87.8, 93.6]	0.999 [0.945, 1.056]
Togo	92.8 [88.9, 95.4]	92.2 [89.1, 94.4]	0.993 [0.950, 1.038]

Notes: 95% confidence intervals are shown in brackets.

Table S13. Percentage of children 6–23 months old receiving minimum dietary diversity by urban-rural residency.

	Urban	Rural	Rural/Urban
	%	%	Relative difference
Pooled	30.4 [29.5, 31.3]	18.9 [18.4, 19.3]	0.622 [0.598, 0.646]
Low-Income Countries	27.5 [26.2, 28.9]	15.6 [14.9, 16.3]	0.567 [0.529, 0.606]
Lower-Middle-Income Countries	30.5 [29.5, 31.6]	20.3 [19.7, 20.9]	0.666 [0.636, 0.697]
Upper-Middle-Income Countries	43.1 [39.2, 47.1]	32.0 [28.1, 36.3]	0.744 [0.635, 0.871]
East Asia and Pacific	56.8 [53.8, 59.7]	39.0 [36.8, 41.3]	0.687 [0.637, 0.742]
Cambodia	28.4 [23.0, 34.5]	30.2 [27.4, 33.2]	1.064 [0.851, 1.331]
Indonesia	60.4 [57.2, 63.4]	46.5 [43.6, 49.4]	0.770 [0.711, 0.835]
Myanmar	29.0 [22.2, 36.9]	18.7 [15.5, 22.3]	0.645 [0.473, 0.879]
Papua New Guinea	45.5 [37.0, 54.4]	29.7 [26.3, 33.4]	0.653 [0.522, 0.818]
Timor-Leste	38.6 [30.7, 47.2]	22.7 [19.9, 25.8]	0.589 [0.459, 0.755]
Eastern and Southern Africa	32.2 [30.1, 34.4]	15.6 [14.7, 16.6]	0.485 [0.443, 0.531]
Burundi	30.0 [24.5, 36.2]	15.4 [13.3, 17.8]	0.513 [0.404, 0.652]
Comoros	28.6 [20.7, 38.1]	18.7 [14.7, 23.5]	0.653 [0.447, 0.956]
Ethiopia	9.6 [6.5, 14.1]	3.6 [2.7, 4.7]	0.369 [0.229, 0.596]
Kenya	50.7 [44.8, 56.5]	28.5 [25.9, 31.2]	0.562 [0.485, 0.651]
Lesotho	26.7 [20.1, 34.6]	13.7 [10.9, 17.1]	0.514 [0.363, 0.729]
Malawi	36.7 [32.0, 41.7]	25.6 [24.0, 27.2]	0.697 [0.603, 0.806]
Mozambique	27.1 [23.1, 31.6]	28.6 [25.7, 31.6]	1.052 [0.871, 1.270]
Namibia	39.6 [33.8, 45.8]	12.9 [10.2, 16.3]	0.327 [0.247, 0.432]
Rwanda	45.7 [38.9, 52.6]	22.1 [20.0, 24.3]	0.484 [0.406, 0.578]
South Africa	43.0 [37.9, 48.4]	31.2 [26.1, 36.8]	0.724 [0.588, 0.893]
Tanzania	32.1 [27.5, 37.2]	17.0 [14.9, 19.4]	0.531 [0.434, 0.649]
Uganda	29.4 [23.7, 35.8]	15.3 [13.1, 17.8]	0.521 [0.404, 0.672]
Zambia	24.2 [20.7, 28.1]	15.3 [13.5, 17.4]	0.634 [0.520, 0.773]
Europe and Central Asia	35.2 [31.0, 39.7]	35.9 [32.9, 39.1]	1.020 [0.877, 1.185]
Kyrgyz Republic	31.9 [25.6, 38.9]	37.8 [32.8, 43.0]	1.184 [0.925, 1.515]
Tajikistan	37.7 [32.2, 43.6]	35.0 [31.1, 39.0]	0.927 [0.768, 1.119]
Latin America and Caribbean	53.9 [51.4, 56.5]	44.5 [42.4, 46.6]	0.826 [0.772, 0.883]
Dominican Republic	49.3 [43.5, 55.2]	42.8 [36.9, 48.8]	0.868 [0.723, 1.042]
Guatemala	66.3 [62.9, 69.4]	53.9 [51.2, 56.5]	0.813 [0.759, 0.872]
Haiti	26.6 [22.2, 31.6]	21.5 [17.5, 26.1]	0.808 [0.620, 1.052]
Honduras	64.6 [61.3, 67.8]	57.8 [54.6, 60.9]	0.894 [0.830, 0.963]
Middle East and North Africa	34.8 [32.6, 37.1]	30.7 [28.9, 32.4]	0.880 [0.807, 0.960]
Egypt	33.7 [31.0, 36.5]	35.2 [33.1, 37.4]	1.045 [0.943, 1.157]
Jordan	46.7 [40.1, 53.3]	31.4 [26.0, 37.3]	0.672 [0.535, 0.845]
Yemen	36.5 [32.4, 40.8]	15.7 [13.8, 17.8]	0.431 [0.364, 0.510]
South Asia	24.6 [23.5, 25.8]	18.0 [17.4, 18.6]	0.731 [0.689, 0.775]
Afghanistan	27.6 [22.0, 34.1]	13.2 [10.9, 15.8]	0.478 [0.359, 0.637]
Bangladesh	32.4 [28.6, 36.5]	21.3 [19.0, 23.7]	0.655 [0.555, 0.773]
India	24.0 [22.7, 25.4]	17.9 [17.4, 18.5]	0.747 [0.701, 0.797]
Maldives	69.8 [58.5, 79.1]	69.0 [65.0, 72.8]	0.989 [0.848, 1.154]

	Urban	Rural	Rural/Urban
	%	%	Relative difference
Nepal	44.2 [37.0, 51.7]	25.5 [21.2, 30.4]	0.577 [0.452, 0.736]
Pakistan	22.5 [19.1, 26.3]	15.7 [13.2, 18.6]	0.700 [0.553, 0.885]
West and Central Africa	23.2 [21.7, 24.7]	11.9 [11.0, 12.8]	0.513 [0.464, 0.567]
Benin	33.8 [30.6, 37.1]	25.8 [23.4, 28.3]	0.763 [0.666, 0.874]
Burkina Faso	11.3 [8.6, 14.6]	3.8 [3.1, 4.8]	0.341 [0.241, 0.483]
Cameroon	35.4 [31.7, 39.3]	20.1 [16.5, 24.3]	0.568 [0.456, 0.708]
Chad	17.8 [14.1, 22.1]	7.1 [5.6, 8.9]	0.400 [0.291, 0.549]
Congo	18.0 [14.5, 22.0]	13.0 [10.4, 16.1]	0.723 [0.537, 0.974]
Congo Democratic Republic	25.3 [21.4, 29.7]	13.8 [10.8, 17.4]	0.543 [0.407, 0.725]
Cote D'Ivoire	13.0 [9.9, 16.9]	4.1 [3.1, 5.5]	0.318 [0.214, 0.472]
Gabon	17.9 [13.8, 22.9]	14.9 [10.5, 20.8]	0.832 [0.545, 1.271]
Gambia	14.0 [10.2, 18.9]	7.1 [5.3, 9.3]	0.504 [0.333, 0.763]
Ghana	29.2 [24.2, 34.8]	20.8 [15.8, 27.1]	0.713 [0.516, 0.986]
Guinea	13.4 [9.7, 18.2]	3.8 [2.6, 5.6]	0.284 [0.172, 0.469]
Liberia	9.0 [6.3, 12.5]	7.5 [5.8, 9.6]	0.833 [0.547, 1.268]
Mali	27.3 [23.4, 31.6]	16.3 [13.8, 19.1]	0.596 [0.477, 0.743]
Niger	26.3 [22.1, 30.9]	5.4 [4.4, 6.7]	0.206 [0.158, 0.269]
Nigeria	22.1 [19.5, 25.0]	11.9 [10.5, 13.4]	0.537 [0.451, 0.639]
Senegal	32.5 [27.4, 38.0]	17.8 [15.5, 20.4]	0.549 [0.445, 0.678]
Sierra Leone	20.6 [15.5, 26.9]	11.3 [9.4, 13.6]	0.549 [0.394, 0.764]
Togo	24.4 [20.4, 28.9]	14.8 [12.6, 17.4]	0.609 [0.481, 0.771]

Notes: 95% confidence intervals are shown in brackets.

Table S14. Percentage of children 6–23 months old receiving vitamin-A-rich foods by urban-rural residency.

	Urban	Rural	Rural/Urban
	%	%	Relative difference
Pooled	61.6 [60.7, 62.6]	52.0 [51.3, 52.6]	0.843 [0.827, 0.860]
Low-Income Countries	66.9 [65.2, 68.6]	56.4 [55.1, 57.6]	0.842 [0.815, 0.871]
Lower-Middle-Income Countries	59.2 [58.1, 60.3]	49.5 [48.8, 50.3]	0.836 [0.816, 0.856]
Upper-Middle-Income Countries	76.4 [72.3, 80.0]	64.9 [60.6, 68.9]	0.849 [0.783, 0.921]
East Asia and Pacific	84.9 [82.6, 87.0]	79.0 [77.2, 80.7]	0.930 [0.899, 0.963]
Cambodia	78.6 [72.3, 83.8]	86.5 [84.2, 88.4]	1.100 [1.019, 1.187]
Indonesia	85.7 [83.1, 87.9]	81.2 [78.7, 83.3]	0.947 [0.910, 0.986]
Myanmar	78.8 [70.4, 85.4]	66.3 [62.2, 70.2]	0.841 [0.752, 0.940]
Papua New Guinea	92.5 [87.2, 95.7]	86.1 [83.6, 88.3]	0.931 [0.885, 0.980]
Timor-Leste	72.1 [66.4, 77.2]	66.3 [62.7, 69.6]	0.919 [0.840, 1.006]
Eastern and Southern Africa	70.5 [67.9, 73.0]	56.1 [54.1, 58.1]	0.795 [0.756, 0.837]
Burundi	79.3 [75.0, 83.0]	83.9 [81.9, 85.7]	1.059 [1.003, 1.118]
Comoros	73.3 [65.3, 80.0]	70.1 [64.7, 75.1]	0.957 [0.846, 1.083]
Ethiopia	37.6 [30.5, 45.4]	23.8 [20.9, 26.9]	0.632 [0.500, 0.799]
Kenya	84.2 [80.6, 87.2]	65.5 [62.5, 68.3]	0.778 [0.733, 0.825]
Lesotho	70.0 [60.2, 78.2]	58.1 [53.4, 62.6]	0.830 [0.714, 0.965]
Malawi	78.7 [73.4, 83.1]	74.4 [72.7, 76.0]	0.945 [0.886, 1.009]
Mozambique	68.9 [65.5, 72.1]	72.0 [69.2, 74.5]	1.045 [0.984, 1.110]
Namibia	75.3 [70.3, 79.7]	67.9 [63.7, 71.8]	0.902 [0.828, 0.983]
Rwanda	82.3 [77.4, 86.4]	71.6 [69.4, 73.7]	0.870 [0.818, 0.924]
South Africa	77.2 [71.6, 82.0]	63.0 [57.4, 68.3]	0.817 [0.732, 0.911]
Tanzania	78.9 [74.4, 82.8]	72.5 [69.3, 75.6]	0.919 [0.858, 0.984]
Uganda	63.8 [57.8, 69.4]	62.3 [59.0, 65.6]	0.977 [0.880, 1.085]
Zambia	75.7 [72.6, 78.5]	75.0 [72.7, 77.1]	0.991 [0.944, 1.040]
Europe and Central Asia	57.6 [52.5, 62.5]	57.1 [53.9, 60.2]	0.992 [0.895, 1.099]
Kyrgyz Republic	64.4 [56.5, 71.6]	65.9 [60.9, 70.5]	1.023 [0.892, 1.173]
Tajikistan	52.5 [46.3, 58.7]	52.5 [48.6, 56.4]	0.999 [0.870, 1.148]
Latin America and Caribbean	78.8 [76.9, 80.5]	74.1 [72.5, 75.7]	0.941 [0.911, 0.971]
Dominican Republic	76.2 [71.7, 80.2]	70.4 [64.7, 75.5]	0.924 [0.840, 1.015]

	Urban	Rural	Rural/Urban
	%	%	Relative difference
Guatemala	85.2 [82.7, 87.4]	77.7 [75.5, 79.8]	0.912 [0.877, 0.948]
Haiti	65.4 [60.9, 69.7]	65.8 [61.8, 69.5]	1.005 [0.920, 1.098]
Honduras	83.8 [81.1, 86.2]	80.7 [78.5, 82.7]	0.963 [0.926, 1.002]
Middle East and North Africa	61.9 [59.4, 64.3]	56.6 [54.8, 58.5]	0.916 [0.869, 0.965]
Egypt	60.3 [57.2, 63.3]	61.5 [59.3, 63.7]	1.021 [0.959, 1.086]
Jordan	72.0 [66.0, 77.3]	69.0 [61.5, 75.6]	0.958 [0.842, 1.089]
Yemen	65.7 [61.9, 69.3]	40.4 [37.7, 43.1]	0.614 [0.563, 0.670]
South Asia	50.0 [48.6, 51.4]	44.6 [43.7, 45.4]	0.891 [0.862, 0.922]
Afghanistan	53.6 [47.8, 59.4]	45.2 [40.4, 50.1]	0.843 [0.724, 0.981]
Bangladesh	67.5 [62.9, 71.8]	63.1 [60.2, 65.9]	0.935 [0.864, 1.012]
India	47.5 [46.1, 49.0]	42.6 [42.0, 43.3]	0.897 [0.867, 0.928]
Maldives	88.3 [79.1, 93.8]	88.4 [85.3, 91.0]	1.001 [0.921, 1.089]
Nepal	57.7 [50.3, 64.8]	45.5 [41.5, 49.7]	0.789 [0.677, 0.919]
Pakistan	52.5 [47.5, 57.5]	41.1 [36.8, 45.5]	0.782 [0.678, 0.901]
West and Central Africa	64.5 [62.7, 66.2]	54.0 [52.5, 55.5]	0.838 [0.806, 0.870]
Benin	56.7 [53.1, 60.3]	50.9 [48.1, 53.8]	0.898 [0.826, 0.977]
Burkina Faso	47.4 [42.4, 52.5]	31.7 [29.2, 34.3]	0.668 [0.585, 0.762]
Cameroon	80.4 [76.9, 83.5]	73.7 [69.6, 77.5]	0.917 [0.858, 0.980]
Chad	55.3 [49.8, 60.6]	43.4 [40.2, 46.7]	0.786 [0.696, 0.889]
Congo	73.0 [68.2, 77.3]	82.3 [80.0, 84.4]	1.128 [1.054, 1.206]
Congo Democratic Republic	82.6 [79.5, 85.3]	81.3 [78.9, 83.5]	0.984 [0.942, 1.029]
Cote D'Ivoire	64.9 [59.4, 70.0]	56.6 [52.6, 60.6]	0.872 [0.784, 0.971]
Gabon	68.9 [64.6, 72.9]	69.9 [65.1, 74.2]	1.015 [0.929, 1.108]
Gambia	49.4 [43.8, 55.1]	45.9 [41.2, 50.7]	0.930 [0.798, 1.084]
Ghana	68.1 [63.0, 72.8]	66.4 [61.0, 71.5]	0.976 [0.877, 1.085]
Guinea	39.7 [33.0, 46.8]	22.5 [19.1, 26.3]	0.566 [0.448, 0.716]
Liberia	58.8 [54.9, 62.6]	57.4 [53.9, 60.8]	0.976 [0.893, 1.067]
Mali	63.1 [57.7, 68.2]	52.5 [49.0, 56.1]	0.833 [0.748, 0.927]
Niger	60.2 [55.4, 64.9]	33.2 [30.3, 36.2]	0.552 [0.490, 0.621]
Nigeria	58.9 [55.8, 61.9]	47.6 [45.3, 50.0]	0.808 [0.752, 0.868]
Senegal	68.8 [64.6, 72.8]	58.8 [55.5, 62.1]	0.855 [0.788, 0.927]
Sierra Leone	51.2 [45.0, 57.4]	44.4 [40.7, 48.1]	0.866 [0.749, 1.003]
Togo	68.0 [63.2, 72.5]	68.9 [65.9, 71.7]	1.013 [0.935, 1.097]

Notes: 95% confidence intervals are shown in brackets.

Table S15. Percentage of children 6–23 months old receiving vitamin A supplementation by urban-rural residency.

	Urban	Rural	Rural/Urban
	%	%	Relative difference
Pooled	62.9 [61.9, 64.0]	56.9 [56.2, 57.6]	0.904 [0.885, 0.923]
Low-Income Countries	65.2 [63.4, 66.9]	58.0 [56.9, 59.0]	0.889 [0.861, 0.919]
Lower-Middle-Income Countries	62.0 [60.6, 63.3]	56.1 [55.2, 56.9]	0.904 [0.880, 0.929]
Upper-Middle-Income Countries	67.7 [63.5, 71.5]	74.2 [70.2, 77.8]	1.097 [1.014, 1.186]
East Asia and Pacific	68.3 [65.2, 71.2]	62.5 [60.3, 64.7]	0.916 [0.866, 0.969]
Cambodia	63.3 [57.4, 68.8]	66.5 [63.3, 69.6]	1.051 [0.949, 1.163]
Indonesia	70.5 [67.2, 73.6]	67.1 [64.3, 69.8]	0.952 [0.895, 1.012]
Myanmar	47.8 [39.7, 56.0]	50.1 [45.2, 55.0]	1.049 [0.862, 1.275]
Papua New Guinea	55.8 [48.1, 63.3]	38.7 [35.0, 42.5]	0.693 [0.586, 0.819]
Timor-Leste	66.3 [59.8, 72.2]	60.3 [56.3, 64.1]	0.909 [0.812, 1.018]
Eastern and Southern Africa	68.7 [66.2, 71.1]	60.1 [58.5, 61.6]	0.876 [0.838, 0.915]
Burundi	87.8 [82.1, 91.9]	80.3 [77.9, 82.5]	0.914 [0.860, 0.971]
Comoros	50.6 [43.2, 58.0]	50.9 [44.8, 57.0]	1.006 [0.833, 1.215]
Ethiopia	52.0 [43.6, 60.2]	51.4 [48.1, 54.8]	0.990 [0.833, 1.177]
Kenya	81.1 [76.9, 84.6]	74.1 [71.1, 76.9]	0.914 [0.860, 0.972]
Lesotho	81.7 [74.9, 86.9]	70.4 [66.2, 74.3]	0.862 [0.786, 0.946]
Malawi	80.4 [75.2, 84.7]	85.0 [83.5, 86.3]	1.057 [0.994, 1.123]

	Urban	Rural	Rural/Urban
	%	%	Relative difference
Mozambique	82.1 [78.6, 85.1]	66.0 [62.9, 69.1]	0.805 [0.757, 0.855]
Namibia	86.3 [82.4, 89.4]	87.8 [84.5, 90.5]	1.018 [0.966, 1.072]
Rwanda	92.9 [88.3, 95.8]	87.2 [85.3, 88.9]	0.938 [0.898, 0.980]
South Africa	75.4 [69.6, 80.4]	82.7 [77.7, 86.8]	1.097 [1.003, 1.200]
Tanzania	53.1 [48.7, 57.5]	39.5 [36.3, 42.7]	0.742 [0.661, 0.834]
Uganda	68.5 [62.7, 73.8]	60.7 [57.0, 64.3]	0.886 [0.802, 0.979]
Zambia	72.5 [69.0, 75.6]	66.9 [64.3, 69.4]	0.923 [0.870, 0.979]
Europe and Central Asia	55.3 [50.4, 60.0]	54.2 [50.4, 57.8]	0.980 [0.877, 1.094]
Kyrgyz Republic	49.1 [41.9, 56.3]	42.1 [37.2, 47.2]	0.858 [0.711, 1.035]
Tajikistan	59.9 [53.6, 65.9]	60.5 [55.7, 65.1]	1.010 [0.889, 1.149]
Latin America and Caribbean	56.4 [53.8, 58.9]	59.3 [57.1, 61.4]	1.052 [0.993, 1.115]
Dominican Republic	32.8 [28.1, 37.9]	33.9 [29.4, 38.8]	1.035 [0.843, 1.270]
Guatemala	59.1 [55.2, 62.8]	62.2 [59.0, 65.2]	1.052 [0.971, 1.141]
Haiti	60.1 [54.1, 65.9]	52.3 [47.5, 57.1]	0.870 [0.761, 0.995]
Honduras	78.5 [75.2, 81.6]	81.6 [79.2, 83.8]	1.039 [0.989, 1.092]
Middle East and North Africa	28.2 [25.7, 30.9]	27.9 [26.2, 29.6]	0.988 [0.884, 1.105]
Egypt	19.8 [17.3, 22.5]	21.0 [19.2, 22.9]	1.060 [0.905, 1.241]
Jordan	53.7 [47.0, 60.2]	49.4 [42.7, 56.2]	0.921 [0.767, 1.106]
Yemen	56.9 [51.2, 62.3]	49.9 [47.1, 52.7]	0.878 [0.784, 0.982]
South Asia	63.3 [61.6, 64.9]	60.6 [59.8, 61.5]	0.958 [0.930, 0.987]
Afghanistan	50.9 [45.1, 56.7]	49.1 [42.2, 56.0]	0.965 [0.805, 1.157]
Bangladesh	49.9 [44.4, 55.4]	52.5 [49.0, 56.0]	1.053 [0.926, 1.198]
India	63.7 [62.3, 65.1]	59.4 [58.7, 60.1]	0.932 [0.910, 0.955]
Maldives	53.8 [41.0, 66.2]	55.6 [51.2, 59.9]	1.033 [0.811, 1.315]
Nepal	71.5 [64.6, 77.6]	76.4 [72.9, 79.6]	1.069 [0.967, 1.181]
Pakistan	69.4 [61.9, 75.9]	73.0 [69.0, 76.6]	1.052 [0.939, 1.178]
West and Central Africa	64.6 [62.1, 67.1]	50.3 [48.6, 52.1]	0.779 [0.739, 0.821]
Benin	57.0 [52.8, 61.1]	47.0 [44.0, 50.0]	0.824 [0.748, 0.908]
Burkina Faso	71.0 [66.3, 75.4]	65.0 [61.9, 68.1]	0.916 [0.845, 0.992]
Cameroon	73.8 [68.6, 78.5]	80.2 [76.8, 83.2]	1.086 [1.005, 1.174]
Chad	47.2 [41.1, 53.4]	41.9 [38.8, 45.0]	0.887 [0.764, 1.030]
Congo	67.6 [61.2, 73.3]	61.4 [56.9, 65.7]	0.909 [0.811, 1.018]
Congo Democratic Republic	81.2 [77.8, 84.2]	62.8 [59.1, 66.3]	0.773 [0.721, 0.829]
Cote D'Ivoire	72.2 [66.5, 77.2]	54.9 [50.2, 59.5]	0.760 [0.680, 0.850]
Gabon	56.0 [51.6, 60.4]	60.5 [55.1, 65.5]	1.079 [0.962, 1.212]
Gambia	73.1 [67.4, 78.2]	76.6 [72.4, 80.3]	1.047 [0.958, 1.145]
Ghana	70.1 [64.4, 75.1]	70.8 [63.2, 77.3]	1.010 [0.891, 1.144]
Guinea	53.1 [45.4, 60.6]	34.6 [30.1, 39.4]	0.651 [0.536, 0.792]
Liberia	68.2 [62.0, 73.8]	56.9 [52.2, 61.4]	0.834 [0.742, 0.938]
Mali	69.6 [65.0, 73.8]	55.4 [51.6, 59.1]	0.796 [0.726, 0.873]
Niger	73.3 [68.4, 77.6]	59.4 [55.5, 63.1]	0.810 [0.741, 0.886]
Nigeria	54.1 [49.6, 58.5]	33.5 [30.7, 36.5]	0.619 [0.549, 0.698]
Senegal	82.8 [78.9, 86.1]	74.2 [71.0, 77.2]	0.896 [0.844, 0.951]
Sierra Leone	87.3 [82.5, 90.9]	83.6 [80.6, 86.2]	0.958 [0.904, 1.015]
Togo	80.6 [76.2, 84.3]	81.8 [78.3, 84.8]	1.015 [0.953, 1.081]

Notes: 95% confidence intervals are shown in brackets.

Table S16. Percentage of children 6–23 months old receiving either vitamin-A-rich foods or vitamin A supplementation by urban-rural residency.

	Urban	Rural	Rural/Urban
	%	%	Relative difference
Pooled	82.7 [81.9, 83.4]	76.4 [75.9, 77.0]	0.925 [0.915, 0.935]
Low-Income Countries	85.1 [83.6, 86.5]	78.6 [77.7, 79.5]	0.924 [0.906, 0.943]
Lower-Middle-Income Countries	81.5 [80.6, 82.4]	75.1 [74.5, 75.7]	0.921 [0.909, 0.934]
Upper-Middle-Income Countries	90.3 [87.6, 92.5]	90.5 [87.7, 92.7]	1.002 [0.965, 1.042]

	Urban	Rural	Rural/Urban
	%	%	Relative difference
East Asia and Pacific	91.9 [90.1, 93.5]	88.8 [87.4, 90.0]	0.966 [0.943, 0.988]
Cambodia	89.7 [85.1, 93.0]	93.1 [91.4, 94.5]	1.038 [0.991, 1.087]
Indonesia	92.6 [90.7, 94.2]	90.6 [88.9, 92.1]	0.978 [0.954, 1.004]
Myanmar	84.9 [75.7, 91.0]	79.8 [76.1, 83.0]	0.940 [0.853, 1.036]
Papua New Guinea	95.9 [92.4, 97.8]	90.1 [87.9, 92.0]	0.940 [0.908, 0.973]
Timor-Leste	89.9 [86.2, 92.6]	83.7 [80.8, 86.2]	0.931 [0.888, 0.977]
Eastern and Southern Africa	87.3 [84.9, 89.3]	79.1 [77.6, 80.6]	0.907 [0.878, 0.936]
Burundi	96.9 [94.0, 98.4]	95.9 [94.8, 96.8]	0.990 [0.967, 1.013]
Comoros	84.8 [78.4, 89.5]	83.2 [78.7, 86.8]	0.981 [0.905, 1.063]
Ethiopia	64.8 [54.7, 73.7]	61.4 [58.1, 64.6]	0.948 [0.811, 1.108]
Kenya	95.4 [93.2, 96.9]	88.5 [86.3, 90.4]	0.928 [0.901, 0.956]
Lesotho	93.4 [88.1, 96.4]	85.4 [82.2, 88.0]	0.914 [0.867, 0.965]
Malawi	93.7 [89.3, 96.3]	94.3 [93.4, 95.1]	1.007 [0.970, 1.044]
Mozambique	91.6 [89.1, 93.5]	87.7 [85.7, 89.5]	0.958 [0.928, 0.989]
Namibia	95.4 [92.3, 97.3]	94.1 [91.9, 95.7]	0.986 [0.955, 1.018]
Rwanda	98.0 [96.0, 99.0]	93.8 [92.4, 94.9]	0.957 [0.939, 0.975]
South Africa	91.7 [87.7, 94.5]	92.6 [88.8, 95.1]	1.009 [0.961, 1.060]
Tanzania	87.9 [84.5, 90.6]	80.7 [78.3, 82.9]	0.918 [0.878, 0.960]
Uganda	89.2 [85.0, 92.3]	82.6 [79.6, 85.3]	0.927 [0.880, 0.976]
Zambia	90.2 [88.0, 92.0]	88.2 [86.4, 89.8]	0.977 [0.949, 1.006]
Europe and Central Asia	78.9 [75.0, 82.4]	78.9 [75.8, 81.6]	0.999 [0.942, 1.060]
Kyrgyz Republic	82.9 [76.8, 87.6]	80.5 [76.1, 84.2]	0.971 [0.896, 1.053]
Tajikistan	76.0 [70.6, 80.6]	78.0 [73.9, 81.7]	1.027 [0.946, 1.114]
Latin America and Caribbean	89.3 [87.9, 90.6]	87.1 [85.7, 88.5]	0.975 [0.954, 0.997]
Dominican Republic	83.6 [79.6, 87.0]	80.0 [75.3, 84.0]	0.957 [0.893, 1.026]
Guatemala	91.9 [90.1, 93.5]	89.6 [87.8, 91.1]	0.974 [0.950, 0.999]
Haiti	85.1 [81.2, 88.2]	81.3 [77.3, 84.8]	0.956 [0.899, 1.016]
Honduras	96.3 [94.7, 97.4]	95.4 [94.2, 96.3]	0.990 [0.973, 1.008]
Middle East and North Africa	71.2 [68.7, 73.5]	68.4 [66.7, 70.1]	0.962 [0.922, 1.003]
Egypt	67.4 [64.4, 70.3]	68.6 [66.5, 70.7]	1.018 [0.965, 1.074]
Jordan	89.5 [85.2, 92.6]	88.2 [82.4, 92.2]	0.985 [0.921, 1.054]
Yemen	82.2 [78.8, 85.2]	67.3 [64.9, 69.7]	0.819 [0.777, 0.863]
South Asia	78.9 [77.7, 80.0]	75.3 [74.6, 76.0]	0.955 [0.939, 0.972]
Afghanistan	75.6 [70.7, 80.0]	70.7 [66.6, 74.6]	0.936 [0.861, 1.016]
Bangladesh	79.9 [75.4, 83.7]	79.0 [76.4, 81.5]	0.990 [0.931, 1.052]
India	77.5 [76.4, 78.6]	73.3 [72.8, 73.9]	0.946 [0.931, 0.962]
Maldives	92.7 [84.2, 96.8]	93.5 [90.6, 95.6]	1.009 [0.944, 1.078]
Nepal	80.4 [74.4, 85.3]	83.5 [80.2, 86.3]	1.038 [0.962, 1.120]
Pakistan	85.2 [80.5, 88.9]	82.7 [79.4, 85.6]	0.971 [0.914, 1.032]
West and Central Africa	84.2 [82.5, 85.8]	73.9 [72.5, 75.2]	0.877 [0.853, 0.901]
Benin	77.5 [74.4, 80.4]	69.9 [67.2, 72.4]	0.901 [0.854, 0.951]
Burkina Faso	82.3 [78.0, 85.9]	74.0 [71.3, 76.6]	0.900 [0.848, 0.955]
Cameroon	95.6 [92.6, 97.4]	95.1 [93.2, 96.5]	0.995 [0.966, 1.025]
Chad	74.0 [68.7, 78.6]	64.1 [61.0, 67.1]	0.867 [0.799, 0.940]
Congo	89.4 [85.6, 92.3]	93.5 [91.8, 94.8]	1.045 [1.004, 1.088]
Congo Democratic Republic	96.1 [94.6, 97.2]	91.6 [89.9, 93.0]	0.953 [0.932, 0.974]
Cote D'Ivoire	88.0 [83.8, 91.2]	77.5 [73.8, 80.8]	0.881 [0.829, 0.936]
Gabon	84.7 [81.2, 87.6]	84.6 [80.9, 87.7]	0.999 [0.946, 1.056]
Gambia	85.0 [80.7, 88.4]	86.2 [83.5, 88.5]	1.014 [0.962, 1.070]
Ghana	87.8 [84.4, 90.4]	85.9 [80.7, 89.9]	0.979 [0.920, 1.043]
Guinea	66.4 [59.1, 73.0]	47.7 [43.0, 52.6]	0.719 [0.622, 0.830]
Liberia	84.4 [80.0, 88.0]	80.8 [77.3, 83.9]	0.957 [0.899, 1.018]
Mali	86.1 [82.8, 88.8]	75.9 [73.1, 78.5]	0.882 [0.839, 0.927]
Niger	87.6 [84.3, 90.3]	71.3 [68.0, 74.4]	0.814 [0.770, 0.861]
Nigeria	78.0 [74.6, 81.0]	63.1 [60.7, 65.5]	0.810 [0.766, 0.857]
Senegal	94.6 [92.9, 95.9]	87.2 [85.2, 88.9]	0.921 [0.898, 0.946]

	Urban	Rural	Rural/Urban
	%	%	Relative difference
Sierra Leone	91.4 [87.6, 94.1]	88.9 [86.5, 91.0]	0.973 [0.932, 1.016]
Togo	92.7 [90.1, 94.7]	92.4 [90.6, 93.9]	0.997 [0.967, 1.027]

Notes: 95% confidence intervals are shown in brackets.

Table S17. Percentage of children under two (6–23 months) and over two (24–59 months) receiving vitamin A supplementation.

	Over two	Under two	Over two/Under two
	%	%	Relative difference
Pooled	58.9 [58.4, 59.5]	59.1 [58.6, 59.7]	1.003 [0.996, 1.011]
Low-Income Countries	60.2 [59.3, 61.1]	62.6 [61.7, 63.5]	1.040 [1.027, 1.052]
Lower-Middle-Income Countries	58.0 [57.3, 58.8]	57.3 [56.6, 58.0]	0.988 [0.978, 0.997]
Upper-Middle-Income Countries	70.7 [67.9, 73.3]	66.7 [64.4, 68.9]	0.944 [0.903, 0.986]
East Asia and Pacific	65.0 [63.3, 66.8]	69.9 [68.5, 71.3]	1.075 [1.046, 1.105]
Cambodia	66.6 [63.7, 69.3]	71.9 [69.5, 74.1]	1.080 [1.038, 1.123]
Indonesia	68.8 [66.6, 70.8]	74.2 [72.5, 75.8]	1.080 [1.046, 1.114]
Myanmar	49.5 [45.3, 53.7]	57.0 [53.6, 60.4]	1.152 [1.063, 1.249]
Papua New Guinea	41.7 [38.1, 45.4]	26.6 [24.1, 29.1]	0.637 [0.584, 0.694]
Timor-Leste	62.0 [58.6, 65.2]	64.6 [61.5, 67.6]	1.042 [1.000, 1.087]
Eastern and Southern Africa	63.1 [61.8, 64.3]	63.1 [61.8, 64.2]	1.000 [0.983, 1.016]
Burundi	81.1 [79.0, 83.1]	80.3 [78.3, 82.2]	0.990 [0.966, 1.014]
Comoros	51.7 [47.1, 56.3]	47.6 [43.5, 51.8]	0.921 [0.840, 1.010]
Ethiopia	51.4 [48.5, 54.3]	54.7 [51.7, 57.6]	1.064 [1.010, 1.121]
Kenya	75.4 [73.7, 77.0]	70.6 [69.0, 72.1]	0.936 [0.913, 0.959]
Lesotho	73.5 [69.9, 76.7]	54.1 [50.6, 57.6]	0.736 [0.686, 0.791]
Malawi	84.1 [82.7, 85.4]	87.1 [86.0, 88.1]	1.036 [1.018, 1.053]
Mozambique	70.5 [67.9, 72.9]	75.1 [72.5, 77.5]	1.065 [1.033, 1.099]
Namibia	87.2 [84.9, 89.2]	88.5 [86.5, 90.3]	1.015 [0.991, 1.040]
Rwanda	88.0 [86.3, 89.5]	95.7 [94.9, 96.4]	1.088 [1.069, 1.107]
South Africa	78.5 [74.8, 81.8]	75.0 [72.1, 77.7]	0.955 [0.908, 1.005]
Tanzania	43.2 [40.4, 45.9]	40.4 [37.9, 43.0]	0.937 [0.879, 1.000]
Uganda	61.6 [58.4, 64.7]	55.7 [52.7, 58.6]	0.904 [0.860, 0.950]
Zambia	69.1 [67.0, 71.0]	78.3 [76.6, 79.9]	1.134 [1.104, 1.165]
Europe and Central Asia	55.3 [52.3, 58.3]	58.7 [55.9, 61.4]	1.060 [1.011, 1.111]
Kyrgyz Republic	44.3 [40.2, 48.5]	44.7 [41.0, 48.5]	1.009 [0.913, 1.114]
Tajikistan	61.6 [57.7, 65.3]	66.3 [62.9, 69.5]	1.076 [1.022, 1.132]
Latin America and Caribbean	58.0 [56.4, 59.6]	43.2 [41.9, 44.5]	0.744 [0.721, 0.769]
Dominican Republic	33.9 [30.5, 37.4]	35.8 [33.1, 38.5]	1.055 [0.940, 1.185]
Guatemala	61.1 [58.7, 63.5]	40.6 [38.5, 42.6]	0.664 [0.630, 0.699]
Haiti	54.6 [50.9, 58.2]	35.8 [33.0, 38.6]	0.655 [0.604, 0.710]
Honduras	80.4 [78.5, 82.3]	65.8 [63.8, 67.7]	0.818 [0.791, 0.846]
Middle East and North Africa	27.9 [26.5, 29.4]	24.7 [23.4, 26.0]	0.885 [0.841, 0.931]
Egypt	20.2 [18.7, 21.7]	15.0 [13.9, 16.2]	0.744 [0.680, 0.814]
Jordan	53.0 [47.7, 58.2]	19.0 [15.6, 22.9]	0.358 [0.292, 0.440]
Yemen	52.1 [49.6, 54.6]	55.2 [53.1, 57.2]	1.060 [1.021, 1.101]
South Asia	61.4 [60.6, 62.2]	61.2 [60.5, 61.9]	0.997 [0.986, 1.007]
Afghanistan	49.7 [44.6, 54.8]	50.7 [46.1, 55.4]	1.021 [0.982, 1.061]
Bangladesh	52.1 [49.1, 55.1]	64.0 [61.3, 66.5]	1.227 [1.165, 1.293]
India	60.6 [60.0, 61.2]	57.6 [57.1, 58.2]	0.951 [0.941, 0.961]
Maldives	55.2 [50.1, 60.3]	72.6 [69.3, 75.7]	1.315 [1.194, 1.448]
Nepal	75.9 [72.7, 78.9]	93.1 [91.7, 94.2]	1.226 [1.178, 1.276]
Pakistan	71.8 [68.2, 75.0]	73.9 [70.8, 76.7]	1.030 [0.996, 1.064]
West and Central Africa	55.4 [53.9, 56.8]	55.7 [54.2, 57.1]	1.006 [0.991, 1.021]
Benin	51.1 [48.7, 53.5]	49.4 [47.3, 51.5]	0.967 [0.930, 1.006]
Burkina Faso	66.0 [63.3, 68.7]	61.8 [59.0, 64.5]	0.936 [0.908, 0.964]
Cameroon	77.0 [74.1, 79.6]	68.1 [65.4, 70.7]	0.884 [0.851, 0.919]

	Over two	Under two	Over two/Under two
	%	%	Relative difference
Chad	43.1 [40.3, 45.8]	46.4 [43.6, 49.2]	1.077 [1.029, 1.126]
Congo	65.6 [61.5, 69.5]	66.0 [62.8, 69.1]	1.007 [0.960, 1.056]
Congo Democratic Republic	69.2 [66.3, 72.0]	72.5 [69.7, 75.1]	1.047 [1.018, 1.076]
Cote D'Ivoire	62.0 [58.2, 65.7]	63.0 [59.2, 66.6]	1.015 [0.967, 1.066]
Gabon	56.3 [52.6, 60.0]	58.8 [54.0, 63.5]	1.044 [0.959, 1.136]
Gambia	75.4 [72.0, 78.4]	61.6 [57.5, 65.5]	0.817 [0.769, 0.868]
Ghana	70.0 [65.3, 74.4]	61.1 [57.7, 64.3]	0.872 [0.817, 0.931]
Guinea	39.3 [35.2, 43.6]	40.9 [37.1, 44.8]	1.040 [0.967, 1.119]
Liberia	62.8 [58.8, 66.5]	61.2 [57.0, 65.3]	0.975 [0.927, 1.026]
Mali	58.3 [55.1, 61.4]	61.7 [58.8, 64.6]	1.059 [1.016, 1.104]
Niger	61.5 [58.1, 64.7]	60.8 [57.9, 63.7]	0.990 [0.953, 1.027]
Nigeria	41.3 [38.8, 43.9]	41.8 [39.2, 44.4]	1.012 [0.977, 1.048]
Senegal	77.8 [75.3, 80.1]	81.0 [78.7, 83.2]	1.042 [1.016, 1.068]
Sierra Leone	84.7 [82.4, 86.8]	87.5 [85.3, 89.4]	1.032 [1.008, 1.058]
Togo	81.1 [78.5, 83.5]	83.5 [80.9, 85.9]	1.030 [1.005, 1.054]

Notes: 95% confidence intervals are shown in brackets.

Table S18. Percentage of children under two (6–23 months) and over two (24–59 months) receiving vitamin A supplementation by sex.

	Females			Males			
	Under two	Over two	Over two/Under two	Under two	Over two	Over two/Under two	
	%	%	Relative difference	%	%	Relative difference	Interaction term
Pooled	59.2 [58.6, 59.8]	59.2 [58.5, 59.9]	0.999 [0.988, 1.010]	59.1 [58.5, 59.7]	58.7 [58.0, 59.3]	1.007 [0.997, 1.018]	1.008 [0.993, 1.024]
Low-Income Countries	62.5 [61.5, 63.5]	61.0 [59.9, 62.0]	1.026 [1.008, 1.044]	62.7 [61.7, 63.7]	59.5 [58.4, 60.6]	1.054 [1.036, 1.072]	1.027 [1.002, 1.053]
Lower-Middle-Income Countries	57.4 [56.6, 58.2]	58.0 [57.1, 58.9]	0.989 [0.975, 1.002]	57.2 [56.5, 58.0]	58.0 [57.2, 58.8]	0.987 [0.974, 0.999]	0.998 [0.979, 1.017]
Upper-Middle-Income Countries	66.7 [63.5, 69.7]	72.9 [69.1, 76.4]	0.914 [0.855, 0.977]	66.7 [63.7, 69.7]	68.6 [64.4, 72.5]	0.973 [0.911, 1.039]	1.064 [0.964, 1.175]
East Asia and Pacific	70.6 [68.8, 72.3]	66.6 [64.2, 68.9]	1.061 [1.020, 1.103]	69.2 [67.5, 70.9]	63.6 [61.2, 66.0]	1.088 [1.046, 1.132]	1.026 [0.970, 1.085]
Cambodia	73.2 [70.3, 76.0]	69.5 [66.0, 72.8]	1.054 [1.001, 1.110]	70.6 [67.8, 73.3]	63.8 [60.2, 67.3]	1.106 [1.042, 1.175]	1.049 [0.969, 1.136]
Indonesia	74.5 [72.4, 76.6]	70.4 [67.6, 73.1]	1.058 [1.012, 1.106]	73.9 [71.9, 75.9]	67.1 [64.2, 70.0]	1.101 [1.052, 1.152]	1.041 [0.975, 1.110]
Myanmar	59.5 [55.5, 63.4]	48.7 [43.5, 53.9]	1.222 [1.091, 1.369]	54.6 [50.5, 58.6]	50.2 [44.7, 55.7]	1.089 [0.975, 1.215]	0.891 [0.762, 1.041]
Papua New Guinea	26.5 [23.5, 29.8]	41.5 [37.4, 45.6]	0.640 [0.561, 0.730]	26.6 [23.6, 29.7]	41.9 [36.6, 47.5]	0.634 [0.545, 0.737]	0.990 [0.790, 1.242]
Timor-Leste	65.7 [62.1, 69.1]	62.1 [58.1, 65.9]	1.058 [0.998, 1.122]	63.6 [60.2, 67.0]	61.9 [57.6, 66.1]	1.028 [0.967, 1.093]	0.971 [0.891, 1.059]
Eastern and Southern Africa	63.1 [61.7, 64.5]	63.5 [62.0, 65.0]	0.994 [0.970, 1.020]	63.0 [61.5, 64.4]	62.7 [61.1, 64.2]	1.005 [0.980, 1.030]	1.010 [0.973, 1.049]
Burundi	79.9 [77.2, 82.4]	80.4 [77.6, 83.0]	0.994 [0.958, 1.031]	80.7 [78.4, 82.8]	81.9 [78.8, 84.6]	0.985 [0.951, 1.021]	0.991 [0.939, 1.046]
Comoros	47.6 [42.3, 53.0]	54.0 [47.8, 60.1]	0.881 [0.766, 1.014]	47.6 [43.1, 52.2]	49.6 [43.6, 55.6]	0.961 [0.844, 1.095]	1.090 [0.895, 1.329]
Ethiopia	55.5 [52.0, 58.9]	52.8 [49.0, 56.7]	1.050 [0.968, 1.138]	54.0 [50.4, 57.5]	50.0 [46.2, 53.9]	1.079 [0.993, 1.172]	1.028 [0.906, 1.165]
Kenya	70.5 [68.7, 72.4]	76.2 [74.0, 78.3]	0.926 [0.895, 0.958]	70.6 [68.7, 72.4]	74.7 [72.4, 76.8]	0.945 [0.912, 0.979]	1.021 [0.971, 1.073]
Lesotho	54.2 [49.6, 58.8]	73.2 [68.5, 77.4]	0.741 [0.671, 0.819]	54.0 [49.3, 58.6]	73.8 [69.1, 78.0]	0.732 [0.663, 0.807]	0.987 [0.861, 1.132]
Malawi	87.0 [85.6, 88.2]	84.8 [83.0, 86.5]	1.025 [1.001, 1.050]	87.2 [85.8, 88.5]	83.4 [81.4, 85.2]	1.046 [1.020, 1.073]	1.021 [0.984, 1.058]
Mozambique	74.4 [71.2, 77.4]	69.1 [65.9, 72.2]	1.077 [1.027, 1.128]	75.7 [73.1, 78.1]	71.9 [68.8, 74.8]	1.053 [1.010, 1.098]	0.978 [0.918, 1.042]
Namibia	88.7 [86.2, 90.8]	88.1 [85.0, 90.6]	1.006 [0.969, 1.045]	88.3 [85.6, 90.6]	86.2 [82.8, 89.0]	1.025 [0.985, 1.067]	1.018 [0.959, 1.082]
Rwanda	95.3 [94.2, 96.2]	87.8 [85.5, 89.8]	1.085 [1.060, 1.111]	96.1 [95.1, 96.9]	88.2 [86.0, 90.1]	1.090 [1.065, 1.116]	1.005 [0.974, 1.037]
South Africa	74.9 [70.9, 78.6]	82.1 [77.0, 86.2]	0.913 [0.847, 0.984]	75.0 [71.0, 78.7]	75.3 [69.6, 80.3]	0.996 [0.922, 1.076]	1.091 [0.974, 1.222]
Tanzania	39.5 [36.5, 42.6]	43.4 [40.0, 46.9]	0.911 [0.830, 1.000]	41.3 [38.4, 44.4]	42.9 [39.5, 46.4]	0.963 [0.883, 1.050]	1.057 [0.932, 1.198]
Uganda	56.0 [52.6, 59.4]	59.3 [55.3, 63.2]	0.945 [0.880, 1.015]	55.3 [51.7, 58.8]	64.0 [60.1, 67.7]	0.864 [0.807, 0.924]	0.914 [0.830, 1.006]
Zambia	78.1 [75.9, 80.1]	70.0 [67.2, 72.6]	1.116 [1.074, 1.159]	78.6 [76.7, 80.4]	68.2 [65.6, 70.7]	1.153 [1.110, 1.198]	1.033 [0.978, 1.092]
Europe and Central Asia	57.3 [54.2, 60.4]	55.3 [51.4, 59.1]	1.037 [0.968, 1.112]	59.9 [56.5, 63.2]	55.4 [51.7, 59.0]	1.082 [1.008, 1.160]	1.043 [0.940, 1.156]
Kyrgyz Republic	44.2 [39.5, 49.0]	40.8 [35.5, 46.3]	1.083 [0.940, 1.248]	45.2 [41.1, 49.4]	47.4 [42.2, 52.7]	0.953 [0.839, 1.082]	0.880 [0.733, 1.057]
Tajikistan	64.7 [60.9, 68.4]	63.1 [58.2, 67.8]	1.026 [0.948, 1.110]	67.7 [63.4, 71.6]	60.1 [55.3, 64.8]	1.126 [1.039, 1.219]	1.097 [0.972, 1.239]
Latin America and Caribbean	42.7 [41.1, 44.3]	56.7 [54.5, 58.9]	0.752 [0.718, 0.788]	43.7 [42.1, 45.2]	59.3 [57.2, 61.3]	0.737 [0.707, 0.768]	0.980 [0.923, 1.040]
Dominican Republic	34.1 [30.2, 38.4]	33.2 [28.1, 38.8]	1.027 [0.842, 1.253]	37.2 [34.1, 40.5]	34.5 [29.7, 39.7]	1.079 [0.928, 1.254]	1.050 [0.808, 1.365]
Guatemala	40.2 [37.8, 42.7]	61.1 [57.8, 64.2]	0.659 [0.614, 0.706]	40.9 [38.4, 43.4]	61.2 [58.1, 64.1]	0.668 [0.625, 0.714]	1.015 [0.929, 1.108]
Haiti	35.9 [32.6, 39.3]	51.8 [47.1, 56.4]	0.692 [0.621, 0.772]	35.7 [32.3, 39.1]	57.4 [52.9, 61.8]	0.621 [0.559, 0.690]	0.897 [0.780, 1.033]
Honduras	65.6 [63.1, 68.0]	79.6 [76.8, 82.1]	0.824 [0.786, 0.863]	66.0 [63.6, 68.3]	81.2 [78.8, 83.4]	0.813 [0.778, 0.849]	0.987 [0.929, 1.048]
Middle East and North Africa	24.8 [23.3, 26.4]	28.2 [26.4, 30.2]	0.879 [0.818, 0.944]	24.6 [23.1, 26.1]	27.6 [25.9, 29.4]	0.891 [0.831, 0.956]	1.014 [0.919, 1.120]

	Females			Males			
	Under two	Over two	Over two/Under two	Under two	Over two	Over two/Under two	
	%	%	Relative difference	%	%	Relative difference	Interaction term
Egypt	15.1 [13.6, 16.7]	19.8 [17.9, 22.0]	0.761 [0.668, 0.866]	14.9 [13.6, 16.3]	20.5 [18.6, 22.4]	0.729 [0.646, 0.821]	0.958 [0.805, 1.139]
Jordan	19.9 [15.2, 25.6]	52.9 [46.2, 59.5]	0.376 [0.283, 0.499]	18.1 [14.5, 22.3]	53.1 [46.4, 59.7]	0.341 [0.270, 0.429]	0.906 [0.658, 1.249]
Yemen	54.9 [52.6, 57.3]	53.6 [50.6, 56.7]	1.024 [0.968, 1.083]	55.4 [53.0, 57.9]	50.6 [47.6, 53.5]	1.097 [1.037, 1.160]	1.071 [0.986, 1.164]
South Asia	61.2 [60.3, 62.0]	61.5 [60.5, 62.5]	0.995 [0.980, 1.011]	61.2 [60.4, 62.0]	61.3 [60.4, 62.3]	0.998 [0.984, 1.012]	1.002 [0.981, 1.024]
Afghanistan	50.9 [45.8, 55.9]	49.6 [44.4, 54.9]	1.024 [0.975, 1.076]	50.6 [46.0, 55.2]	49.8 [44.3, 55.3]	1.017 [0.957, 1.081]	0.993 [0.918, 1.074]
Bangladesh	63.7 [60.5, 66.7]	54.7 [51.2, 58.2]	1.164 [1.084, 1.250]	64.2 [61.1, 67.2]	49.6 [45.7, 53.5]	1.295 [1.205, 1.392]	1.112 [1.008, 1.227]
India	57.7 [57.0, 58.4]	60.3 [59.5, 61.2]	0.956 [0.941, 0.971]	57.6 [57.0, 58.3]	60.9 [60.1, 61.7]	0.947 [0.933, 0.960]	0.991 [0.970, 1.012]
Maldives	70.5 [65.8, 74.8]	54.0 [47.5, 60.3]	1.306 [1.144, 1.491]	74.7 [70.3, 78.6]	56.4 [49.0, 63.6]	1.323 [1.157, 1.513]	1.013 [0.842, 1.218]
Nepal	92.7 [90.4, 94.5]	72.4 [67.9, 76.5]	1.280 [1.204, 1.360]	93.4 [91.7, 94.8]	79.5 [75.4, 83.0]	1.176 [1.118, 1.237]	0.919 [0.850, 0.993]
Pakistan	73.8 [70.0, 77.3]	72.2 [67.6, 76.3]	1.023 [0.968, 1.081]	73.9 [70.9, 76.8]	71.3 [67.6, 74.9]	1.036 [0.993, 1.081]	1.013 [0.942, 1.090]
West and Central Africa	55.5 [54.0, 57.1]	55.7 [54.0, 57.3]	0.997 [0.977, 1.018]	55.8 [54.3, 57.4]	55.1 [53.5, 56.6]	1.014 [0.993, 1.036]	1.017 [0.988, 1.047]
Benin	49.4 [46.9, 51.9]	50.8 [47.8, 53.8]	0.972 [0.914, 1.033]	49.5 [47.0, 51.9]	51.4 [48.4, 54.3]	0.963 [0.911, 1.018]	0.991 [0.909, 1.080]
Burkina Faso	62.0 [59.0, 64.9]	65.8 [62.7, 68.9]	0.941 [0.906, 0.978]	61.6 [58.5, 64.5]	66.2 [63.0, 69.2]	0.930 [0.891, 0.972]	0.988 [0.935, 1.045]
Cameroon	69.0 [66.0, 71.8]	76.1 [72.7, 79.2]	0.907 [0.864, 0.952]	67.1 [63.8, 70.3]	77.9 [74.4, 81.0]	0.861 [0.819, 0.906]	0.950 [0.892, 1.010]
Chad	46.5 [43.6, 49.5]	43.2 [39.8, 46.6]	1.078 [1.006, 1.155]	46.2 [43.0, 49.4]	43.0 [39.7, 46.4]	1.075 [1.000, 1.156]	0.998 [0.895, 1.113]
Congo	65.4 [61.4, 69.3]	65.6 [60.4, 70.5]	0.997 [0.934, 1.064]	66.7 [63.2, 70.0]	65.6 [60.8, 70.0]	1.017 [0.951, 1.089]	1.020 [0.929, 1.121]
Congo Democratic Republic	71.6 [68.5, 74.5]	71.4 [68.2, 74.4]	1.003 [0.964, 1.043]	73.4 [70.4, 76.1]	67.1 [63.4, 70.5]	1.094 [1.050, 1.140]	1.091 [1.029, 1.158]
Cote D'Ivoire	62.6 [57.9, 67.0]	61.9 [57.4, 66.2]	1.010 [0.940, 1.086]	63.5 [59.6, 67.2]	62.2 [57.4, 66.7]	1.021 [0.949, 1.098]	1.010 [0.907, 1.126]
Gabon	62.8 [57.2, 68.0]	58.0 [52.6, 63.3]	1.081 [0.957, 1.222]	55.1 [49.6, 60.4]	54.7 [49.4, 59.8]	1.008 [0.891, 1.140]	0.932 [0.781, 1.112]
Gambia	62.7 [57.9, 67.3]	74.2 [69.6, 78.4]	0.845 [0.776, 0.920]	60.5 [56.5, 64.4]	76.5 [72.8, 79.8]	0.792 [0.742, 0.845]	0.937 [0.856, 1.025]
Ghana	60.3 [56.1, 64.4]	72.4 [66.9, 77.4]	0.833 [0.769, 0.903]	61.7 [58.1, 65.2]	67.8 [62.3, 72.9]	0.910 [0.840, 0.987]	1.093 [0.996, 1.200]
Guinea	40.1 [35.8, 44.6]	38.0 [32.8, 43.5]	1.056 [0.935, 1.192]	41.7 [37.5, 45.9]	40.5 [35.8, 45.4]	1.029 [0.935, 1.132]	0.975 [0.831, 1.143]
Liberia	60.7 [56.1, 65.0]	65.0 [60.2, 69.4]	0.933 [0.862, 1.010]	61.8 [57.0, 66.3]	60.7 [55.2, 66.1]	1.017 [0.941, 1.099]	1.090 [0.967, 1.227]
Mali	61.3 [58.0, 64.5]	59.4 [55.7, 63.0]	1.032 [0.975, 1.092]	62.1 [58.8, 65.3]	57.2 [53.2, 61.0]	1.087 [1.021, 1.156]	1.053 [0.966, 1.148]
Niger	61.6 [58.4, 64.7]	63.0 [59.5, 66.4]	0.977 [0.929, 1.028]	60.1 [56.7, 63.3]	59.9 [55.8, 63.9]	1.003 [0.950, 1.059]	1.026 [0.953, 1.105]
Nigeria	41.8 [39.1, 44.6]	40.5 [37.7, 43.3]	1.032 [0.982, 1.084]	41.8 [39.0, 44.6]	42.1 [39.3, 44.9]	0.992 [0.944, 1.043]	0.961 [0.897, 1.031]
Senegal	80.1 [77.4, 82.5]	77.7 [74.4, 80.8]	1.030 [0.990, 1.072]	82.0 [79.3, 84.4]	77.8 [74.8, 80.5]	1.053 [1.017, 1.091]	1.023 [0.967, 1.081]
Sierra Leone	87.9 [85.3, 90.1]	85.5 [82.6, 88.0]	1.028 [0.995, 1.063]	87.0 [84.6, 89.1]	84.0 [81.0, 86.6]	1.037 [1.002, 1.073]	1.008 [0.961, 1.057]
Togo	82.8 [79.9, 85.3]	81.4 [78.1, 84.3]	1.017 [0.982, 1.054]	84.3 [81.1, 87.0]	80.9 [77.5, 83.9]	1.042 [1.007, 1.079]	1.024 [0.973, 1.079]

Notes: 95% confidence intervals are shown in brackets.

Table S19. Percentage of children under two (6–23 months) and over two (24–59 months) receiving vitamin A supplementation by living standards.

	Wealthiest			Poorest			
	Under two	Over two	Over two/Under two	Under two	Over two	Over two/Under two	

	%	%	Relative difference	%	%	Relative difference	Interaction term
Pooled	65.9 [65.0, 66.9]	65.7 [64.7, 66.8]	1.003 [0.987, 1.019]	51.8 [50.9, 52.7]	51.5 [50.5, 52.5]	1.006 [0.990, 1.022]	1.003 [0.981, 1.026]
Low-Income Countries	69.5 [67.9, 71.1]	67.8 [66.0, 69.6]	1.025 [1.000, 1.050]	56.9 [55.4, 58.4]	54.7 [53.1, 56.3]	1.040 [1.012, 1.068]	1.014 [0.978, 1.052]
Lower-Middle-Income Countries	64.1 [62.9, 65.3]	64.6 [63.2, 65.9]	0.993 [0.972, 1.014]	49.3 [48.1, 50.5]	49.5 [48.2, 50.8]	0.997 [0.977, 1.016]	1.004 [0.975, 1.033]
Upper-Middle-Income Countries	69.1 [63.1, 74.5]	73.1 [64.8, 80.0]	0.945 [0.836, 1.068]	60.9 [57.0, 64.6]	68.4 [62.8, 73.5]	0.890 [0.820, 0.967]	0.942 [0.813, 1.092]
East Asia and Pacific	75.7 [72.8, 78.4]	68.6 [64.7, 72.3]	1.103 [1.036, 1.175]	59.3 [56.7, 61.8]	57.9 [55.0, 60.7]	1.024 [0.970, 1.081]	0.928 [0.855, 1.008]
Cambodia	75.1 [70.3, 79.3]	62.9 [57.1, 68.4]	1.193 [1.080, 1.318]	64.0 [59.4, 68.3]	62.4 [56.8, 67.6]	1.026 [0.936, 1.123]	0.860 [0.751, 0.984]
Indonesia	79.4 [76.2, 82.4]	72.5 [67.8, 76.7]	1.096 [1.022, 1.175]	63.2 [60.0, 66.2]	61.1 [57.5, 64.6]	1.033 [0.969, 1.101]	0.942 [0.858, 1.035]
Myanmar	59.5 [51.0, 67.4]	48.0 [38.2, 58.0]	1.239 [0.994, 1.544]	50.7 [45.1, 56.2]	48.0 [41.3, 54.7]	1.057 [0.916, 1.218]	0.853 [0.654, 1.112]
Papua New Guinea	36.9 [29.4, 45.0]	61.4 [50.7, 71.1]	0.601 [0.504, 0.715]	16.7 [12.9, 21.3]	27.2 [21.2, 34.3]	0.611 [0.479, 0.781]	1.018 [0.754, 1.376]
Timor-Leste	71.0 [65.8, 75.7]	69.8 [63.0, 75.9]	1.017 [0.922, 1.121]	54.4 [48.7, 60.1]	55.5 [48.5, 62.3]	0.981 [0.872, 1.103]	0.965 [0.827, 1.126]
Eastern and Southern Africa	68.2 [65.9, 70.4]	69.6 [67.0, 72.0]	0.980 [0.947, 1.014]	56.7 [54.6, 58.8]	57.4 [55.2, 59.6]	0.988 [0.952, 1.025]	1.008 [0.959, 1.060]
Burundi	85.0 [81.5, 88.0]	85.4 [81.2, 88.8]	0.996 [0.953, 1.040]	81.7 [78.1, 84.9]	79.0 [74.6, 82.8]	1.035 [0.977, 1.096]	1.039 [0.967, 1.116]
Comoros	58.1 [49.3, 66.4]	56.3 [44.3, 67.6]	1.033 [0.835, 1.277]	42.4 [35.7, 49.4]	44.8 [36.6, 53.3]	0.948 [0.775, 1.159]	0.918 [0.684, 1.231]
Ethiopia	61.5 [54.8, 67.7]	54.3 [47.9, 60.5]	1.133 [1.029, 1.248]	45.3 [40.8, 50.0]	46.6 [41.7, 51.6]	0.972 [0.860, 1.098]	0.858 [0.737, 0.999]
Kenya	75.0 [71.1, 78.5]	81.3 [77.5, 84.6]	0.923 [0.868, 0.981]	63.2 [60.5, 65.8]	66.0 [62.5, 69.5]	0.956 [0.915, 0.999]	1.037 [0.962, 1.117]
Lesotho	54.0 [47.4, 60.6]	86.8 [79.5, 91.7]	0.623 [0.541, 0.717]	50.7 [43.9, 57.5]	68.7 [60.8, 75.6]	0.738 [0.634, 0.859]	1.185 [0.961, 1.460]
Malawi	86.4 [82.8, 89.2]	83.1 [79.2, 86.3]	1.040 [0.992, 1.090]	86.6 [84.5, 88.5]	82.6 [79.6, 85.3]	1.048 [1.012, 1.086]	1.008 [0.950, 1.070]
Mozambique	90.5 [88.2, 92.5]	87.9 [84.8, 90.5]	1.030 [0.992, 1.070]	66.4 [61.4, 71.1]	59.4 [53.9, 64.7]	1.118 [1.021, 1.224]	1.085 [0.984, 1.197]
Namibia	76.5 [68.5, 83.0]	80.5 [72.3, 86.7]	0.950 [0.858, 1.053]	90.0 [86.6, 92.5]	87.0 [82.2, 90.6]	1.034 [0.987, 1.083]	1.088 [0.972, 1.218]
Rwanda	97.3 [95.6, 98.3]	90.0 [86.2, 92.8]	1.081 [1.044, 1.119]	94.3 [92.4, 95.7]	87.2 [83.9, 89.9]	1.081 [1.047, 1.117]	1.000 [0.953, 1.049]
South Africa	77.0 [68.9, 83.5]	81.3 [69.5, 89.3]	0.947 [0.825, 1.088]	70.6 [65.1, 75.7]	80.2 [72.7, 86.0]	0.881 [0.801, 0.970]	0.930 [0.786, 1.101]
Tanzania	47.5 [42.8, 52.3]	56.8 [51.3, 62.2]	0.837 [0.740, 0.946]	28.7 [24.7, 33.1]	31.0 [26.3, 36.1]	0.927 [0.797, 1.077]	1.107 [0.911, 1.345]
Uganda	53.9 [49.0, 58.7]	67.5 [62.1, 72.5]	0.798 [0.722, 0.882]	62.4 [57.1, 67.4]	63.0 [56.6, 69.0]	0.990 [0.909, 1.077]	1.240 [1.086, 1.416]
Zambia	85.8 [82.4, 88.7]	75.3 [70.3, 79.7]	1.139 [1.064, 1.220]	71.2 [68.1, 74.2]	63.0 [59.1, 66.7]	1.131 [1.065, 1.201]	0.993 [0.907, 1.087]
Europe and Central Asia	57.6 [52.3, 62.7]	54.5 [49.0, 59.9]	1.056 [0.949, 1.176]	56.2 [51.1, 61.1]	53.3 [47.0, 59.5]	1.054 [0.950, 1.169]	0.998 [0.859, 1.159]
Kyrgyz Republic	42.2 [33.8, 51.1]	53.5 [44.1, 62.6]	0.789 [0.647, 0.963]	47.0 [40.5, 53.5]	44.1 [36.1, 52.4]	1.065 [0.882, 1.285]	1.349 [1.028, 1.770]
Tajikistan	65.5 [60.2, 70.3]	55.2 [48.3, 61.8]	1.186 [1.051, 1.339]	60.9 [54.1, 67.4]	58.2 [49.5, 66.4]	1.047 [0.925, 1.184]	0.882 [0.741, 1.051]
Latin America and Caribbean	40.5 [37.6, 43.6]	61.4 [57.4, 65.3]	0.660 [0.601, 0.726]	42.2 [40.1, 44.3]	52.8 [50.0, 55.7]	0.799 [0.753, 0.848]	1.210 [1.083, 1.353]
Dominican Republic	37.1 [28.6, 46.5]	34.3 [22.4, 48.7]	1.081 [0.696, 1.678]	34.5 [30.7, 38.5]	30.7 [25.6, 36.4]	1.122 [0.932, 1.352]	1.039 [0.643, 1.677]
Guatemala	32.4 [28.4, 36.6]	60.4 [54.5, 66.1]	0.535 [0.455, 0.630]	40.5 [36.9, 44.2]	56.5 [51.8, 61.2]	0.716 [0.648, 0.791]	1.338 [1.107, 1.617]
Haiti	38.4 [32.4, 44.7]	63.7 [56.0, 70.8]	0.602 [0.498, 0.728]	34.2 [30.0, 38.6]	49.5 [43.0, 56.0]	0.690 [0.597, 0.798]	1.146 [0.902, 1.457]
Honduras	62.7 [57.5, 67.6]	82.9 [77.5, 87.3]	0.756 [0.683, 0.837]	66.2 [63.0, 69.3]	80.1 [76.9, 83.0]	0.826 [0.783, 0.872]	1.093 [0.975, 1.226]
Middle East and North Africa	29.6 [26.6, 32.7]	29.9 [26.7, 33.2]	0.990 [0.888, 1.104]	22.4 [20.0, 25.0]	26.8 [23.8, 30.0]	0.837 [0.747, 0.937]	0.845 [0.722, 0.989]
Egypt	18.8 [16.1, 21.7]	21.5 [18.4, 24.9]	0.874 [0.727, 1.051]	12.7 [10.5, 15.3]	18.2 [15.1, 21.9]	0.696 [0.553, 0.875]	0.796 [0.593, 1.068]
Jordan	29.2 [17.3, 44.8]	63.3 [46.6, 77.2]	0.461 [0.280, 0.759]	16.2 [11.7, 22.1]	45.4 [36.7, 54.5]	0.357 [0.248, 0.513]	0.774 [0.421, 1.424]
Yemen	59.8 [55.2, 64.2]	56.9 [51.0, 62.7]	1.050 [0.959, 1.149]	47.5 [43.4, 51.6]	47.0 [42.3, 51.8]	1.010 [0.930, 1.097]	0.962 [0.851, 1.087]
South Asia	64.7 [63.3, 66.1]	64.3 [62.6, 65.9]	1.006 [0.980, 1.033]	54.9 [53.8, 56.0]	54.6 [53.3, 55.8]	1.007 [0.985, 1.028]	1.000 [0.967, 1.035]

	Wealthiest			Poorest			
	Under two	Over two	Over two/Under two	Under two	Over two	Over two/Under two	
	%	%	Relative difference	%	%	Relative difference	Interaction term
Afghanistan	54.0 [47.0, 60.9]	54.1 [46.4, 61.7]	0.998 [0.905, 1.101]	53.5 [48.3, 58.7]	49.2 [43.7, 54.7]	1.088 [1.014, 1.167]	1.090 [0.967, 1.229]
Bangladesh	68.0 [63.4, 72.3]	53.3 [47.0, 59.6]	1.275 [1.126, 1.444]	57.5 [52.8, 62.1]	51.5 [45.7, 57.2]	1.116 [0.999, 1.248]	0.875 [0.740, 1.036]
India	62.2 [60.9, 63.5]	65.0 [63.5, 66.6]	0.956 [0.930, 0.982]	51.4 [50.5, 52.3]	53.1 [52.0, 54.2]	0.968 [0.948, 0.989]	1.013 [0.979, 1.049]
Maldives	60.1 [47.4, 71.5]	51.5 [37.0, 65.6]	1.168 [0.869, 1.571]	76.3 [71.7, 80.4]	55.1 [47.3, 62.7]	1.384 [1.190, 1.611]	1.185 [0.852, 1.648]
Nepal	93.0 [88.9, 95.7]	73.5 [65.7, 80.1]	1.265 [1.144, 1.398]	92.0 [89.3, 94.1]	76.0 [70.4, 80.9]	1.210 [1.133, 1.292]	0.957 [0.848, 1.079]
Pakistan	72.5 [67.0, 77.4]	69.6 [62.9, 75.5]	1.042 [0.957, 1.135]	64.8 [59.3, 69.9]	63.0 [56.7, 68.8]	1.029 [0.956, 1.107]	0.987 [0.883, 1.103]
West and Central Africa	72.1 [70.0, 74.0]	73.0 [70.9, 74.9]	0.988 [0.964, 1.011]	43.2 [40.5, 45.8]	42.0 [39.4, 44.6]	1.028 [0.991, 1.067]	1.041 [0.997, 1.088]
Benin	62.4 [58.2, 66.3]	63.5 [58.3, 68.4]	0.982 [0.913, 1.057]	39.3 [35.9, 42.8]	38.0 [33.9, 42.2]	1.036 [0.941, 1.139]	1.054 [0.934, 1.190]
Burkina Faso	63.9 [59.6, 67.9]	69.9 [65.1, 74.3]	0.914 [0.850, 0.983]	52.1 [47.7, 56.4]	57.0 [52.0, 61.9]	0.913 [0.846, 0.985]	0.999 [0.899, 1.110]
Cameroon	60.8 [56.0, 65.4]	73.0 [66.7, 78.5]	0.833 [0.750, 0.925]	77.3 [72.3, 81.6]	83.5 [78.9, 87.3]	0.925 [0.871, 0.983]	1.111 [0.984, 1.254]
Chad	54.5 [49.9, 59.0]	49.8 [44.3, 55.2]	1.094 [0.991, 1.208]	45.8 [41.2, 50.6]	44.3 [39.0, 49.7]	1.035 [0.925, 1.158]	0.946 [0.816, 1.097]
Congo	75.3 [68.7, 80.9]	78.0 [69.3, 84.7]	0.966 [0.882, 1.058]	63.0 [58.0, 67.8]	58.7 [53.6, 63.7]	1.073 [1.014, 1.136]	1.111 [0.998, 1.236]
Congo Democratic Republic	90.2 [87.5, 92.3]	87.3 [84.0, 90.1]	1.032 [0.998, 1.068]	63.1 [58.9, 67.2]	57.1 [51.6, 62.3]	1.106 [1.034, 1.184]	1.072 [0.993, 1.156]
Cote D'Ivoire	79.8 [74.1, 84.5]	76.7 [68.8, 83.1]	1.040 [0.942, 1.149]	50.7 [44.6, 56.8]	48.6 [42.8, 54.4]	1.043 [0.930, 1.171]	1.003 [0.862, 1.167]
Gabon	52.2 [42.3, 61.9]	53.6 [41.8, 65.1]	0.973 [0.732, 1.293]	67.6 [62.8, 72.0]	62.1 [55.7, 68.2]	1.087 [0.986, 1.200]	1.117 [0.833, 1.500]
Gambia	62.3 [55.3, 68.9]	72.5 [63.7, 79.7]	0.860 [0.766, 0.967]	60.4 [52.9, 67.4]	76.8 [71.1, 81.6]	0.786 [0.713, 0.867]	0.914 [0.787, 1.061]
Ghana	66.1 [59.2, 72.5]	71.7 [64.6, 77.8]	0.923 [0.804, 1.060]	52.0 [45.7, 58.3]	64.1 [56.3, 71.2]	0.812 [0.717, 0.920]	0.880 [0.730, 1.060]
Guinea	54.4 [46.6, 61.9]	62.0 [53.4, 69.9]	0.877 [0.772, 0.996]	35.1 [29.1, 41.7]	30.6 [24.5, 37.4]	1.148 [0.980, 1.346]	1.309 [1.069, 1.604]
Liberia	68.5 [56.8, 78.3]	75.3 [63.4, 84.3]	0.910 [0.787, 1.052]	51.4 [45.9, 56.9]	54.5 [48.5, 60.3]	0.944 [0.856, 1.040]	1.037 [0.871, 1.233]
Mali	73.7 [69.7, 77.5]	68.8 [63.1, 74.0]	1.072 [0.997, 1.153]	54.7 [49.4, 60.0]	54.8 [48.2, 61.2]	0.999 [0.912, 1.096]	0.932 [0.831, 1.046]
Niger	73.9 [69.9, 77.6]	75.1 [70.7, 79.0]	0.985 [0.940, 1.032]	53.0 [48.2, 57.8]	51.8 [46.3, 57.3]	1.024 [0.926, 1.132]	1.039 [0.929, 1.163]
Nigeria	67.4 [63.5, 71.0]	68.7 [64.7, 72.4]	0.981 [0.935, 1.029]	21.6 [17.7, 26.1]	20.3 [17.0, 24.0]	1.068 [0.951, 1.198]	1.088 [0.961, 1.233]
Senegal	82.0 [75.5, 87.0]	82.9 [75.4, 88.5]	0.989 [0.894, 1.094]	73.7 [69.5, 77.5]	67.7 [63.1, 72.0]	1.088 [1.033, 1.146]	1.100 [0.982, 1.232]
Sierra Leone	91.7 [87.2, 94.7]	87.6 [82.2, 91.6]	1.046 [0.999, 1.095]	89.1 [85.8, 91.7]	86.5 [82.5, 89.7]	1.030 [0.991, 1.070]	0.984 [0.927, 1.045]
Togo	82.4 [77.0, 86.7]	83.5 [77.4, 88.2]	0.987 [0.939, 1.037]	87.0 [81.9, 90.8]	84.4 [80.3, 87.7]	1.031 [0.984, 1.080]	1.045 [0.976, 1.118]

Notes: 95% confidence intervals are shown in brackets.

Table S20. Percentage of children under two (6–23 months) and over two (24–59 months) receiving vitamin A supplementation by urban-rural residency.

	Urban			Rural			
	Under two	Over two	Over two/Under two	Under two	Over two	Over two/Under two	
	%	%	Relative difference	%	%	Relative difference	Interaction term
Pooled	62.7 [61.7, 63.6]	63.3 [62.2, 64.3]	0.990 [0.978, 1.003]	57.6 [57.0, 58.3]	57.1 [56.4, 57.7]	1.010 [1.001, 1.018]	1.019 [1.003, 1.035]
Low-Income Countries	67.0 [65.5, 68.5]	66.3 [64.6, 68.0]	1.011 [0.989, 1.032]	61.4 [60.3, 62.4]	58.4 [57.4, 59.4]	1.051 [1.036, 1.066]	1.040 [1.013, 1.067]
Lower-Middle-Income Countries	61.1 [59.9, 62.3]	62.0 [60.7, 63.4]	0.985 [0.969, 1.001]	55.5 [54.6, 56.3]	56.1 [55.2, 56.9]	0.989 [0.978, 1.000]	1.004 [0.984, 1.024]

	Urban			Rural			
	Under two	Over two	Over two/Under two	Under two	Over two	Over two/Under two	
	%	%	Relative difference	%	%	Relative difference	Interaction term
Upper-Middle-Income Countries	66.8 [63.7, 69.8]	68.5 [64.7, 72.0]	0.976 [0.918, 1.036]	66.5 [63.3, 69.5]	74.3 [70.4, 77.9]	0.894 [0.843, 0.949]	0.917 [0.843, 0.997]
East Asia and Pacific	75.8 [73.5, 77.9]	68.3 [65.4, 71.1]	1.110 [1.061, 1.161]	65.6 [63.7, 67.4]	62.7 [60.4, 64.8]	1.046 [1.012, 1.082]	0.943 [0.892, 0.997]
Cambodia	73.6 [68.9, 77.8]	63.0 [57.1, 68.4]	1.169 [1.059, 1.291]	71.6 [68.9, 74.1]	67.3 [64.0, 70.3]	1.064 [1.019, 1.110]	0.910 [0.817, 1.014]
Indonesia	77.6 [75.2, 79.8]	70.5 [67.3, 73.5]	1.101 [1.050, 1.155]	70.8 [68.4, 73.1]	67.1 [64.3, 69.8]	1.055 [1.013, 1.100]	0.959 [0.900, 1.021]
Myanmar	57.7 [51.2, 63.8]	47.6 [39.6, 55.6]	1.212 [1.021, 1.440]	56.9 [52.8, 60.8]	50.1 [45.2, 55.1]	1.134 [1.036, 1.242]	0.935 [0.771, 1.135]
Papua New Guinea	41.3 [31.5, 51.8]	54.2 [46.3, 61.8]	0.762 [0.629, 0.924]	24.9 [22.6, 27.3]	40.1 [36.1, 44.2]	0.620 [0.566, 0.679]	0.814 [0.658, 1.006]
Timor-Leste	68.3 [64.0, 72.4]	65.6 [59.2, 71.5]	1.041 [0.947, 1.145]	63.0 [59.0, 66.9]	60.7 [56.7, 64.5]	1.039 [0.993, 1.087]	0.998 [0.898, 1.108]
Eastern and Southern Africa	69.2 [67.3, 71.0]	70.0 [67.8, 72.1]	0.989 [0.960, 1.018]	61.0 [59.5, 62.4]	60.7 [59.3, 62.1]	1.005 [0.985, 1.025]	1.016 [0.981, 1.053]
Burundi	88.7 [81.2, 93.5]	88.4 [83.0, 92.2]	1.004 [0.963, 1.046]	79.5 [77.4, 81.5]	80.4 [78.1, 82.6]	0.989 [0.963, 1.016]	0.985 [0.938, 1.035]
Comoros	45.3 [38.9, 51.8]	52.8 [45.7, 59.7]	0.858 [0.736, 1.000]	48.5 [43.3, 53.6]	51.2 [45.3, 57.1]	0.946 [0.844, 1.060]	1.102 [0.911, 1.333]
Ethiopia	60.3 [53.1, 67.0]	51.7 [43.7, 59.5]	1.166 [1.028, 1.323]	53.9 [50.7, 57.1]	51.4 [48.2, 54.5]	1.049 [0.992, 1.110]	0.900 [0.784, 1.032]
Kenya	73.9 [71.1, 76.5]	80.5 [77.9, 82.8]	0.918 [0.878, 0.960]	68.7 [66.9, 70.5]	72.6 [70.5, 74.7]	0.946 [0.920, 0.973]	1.030 [0.978, 1.086]
Lesotho	52.5 [45.5, 59.5]	82.0 [76.4, 86.4]	0.641 [0.554, 0.742]	54.8 [50.8, 58.7]	70.3 [66.0, 74.3]	0.779 [0.719, 0.843]	1.215 [1.030, 1.434]
Malawi	80.8 [76.6, 84.3]	80.0 [75.1, 84.2]	1.009 [0.946, 1.077]	88.1 [87.0, 89.1]	84.8 [83.4, 86.1]	1.039 [1.022, 1.056]	1.030 [0.963, 1.100]
Mozambique	86.0 [83.4, 88.2]	82.4 [79.2, 85.2]	1.043 [1.006, 1.082]	70.8 [67.6, 73.9]	66.0 [62.8, 69.0]	1.074 [1.029, 1.119]	1.029 [0.973, 1.087]
Namibia	85.6 [82.1, 88.5]	86.4 [83.0, 89.3]	0.990 [0.953, 1.029]	91.3 [89.3, 93.0]	87.9 [84.7, 90.5]	1.039 [1.008, 1.070]	1.049 [0.999, 1.101]
Rwanda	96.7 [93.5, 98.4]	93.4 [89.1, 96.1]	1.035 [0.998, 1.074]	95.6 [94.7, 96.4]	87.3 [85.4, 89.0]	1.095 [1.074, 1.116]	1.057 [1.015, 1.102]
South Africa	76.0 [72.0, 79.6]	75.9 [70.7, 80.4]	1.001 [0.934, 1.073]	73.2 [69.2, 76.8]	82.8 [77.9, 86.8]	0.884 [0.827, 0.945]	0.883 [0.802, 0.972]
Tanzania	43.8 [40.1, 47.6]	53.2 [48.7, 57.5]	0.823 [0.739, 0.917]	39.3 [36.1, 42.5]	39.4 [36.2, 42.6]	0.998 [0.924, 1.079]	1.213 [1.062, 1.385]
Uganda	56.2 [51.4, 60.9]	68.2 [62.9, 73.1]	0.824 [0.744, 0.912]	55.6 [52.2, 58.9]	60.5 [56.9, 64.0]	0.918 [0.868, 0.970]	1.114 [0.992, 1.251]
Zambia	82.9 [80.2, 85.3]	73.0 [69.6, 76.1]	1.135 [1.088, 1.185]	76.0 [73.8, 78.0]	67.1 [64.5, 69.5]	1.133 [1.095, 1.173]	0.998 [0.945, 1.054]
Europe and Central Asia	57.8 [53.2, 62.2]	56.3 [51.6, 60.9]	1.026 [0.945, 1.114]	58.9 [55.6, 62.3]	55.0 [51.4, 58.7]	1.071 [1.012, 1.133]	1.044 [0.945, 1.153]
Kyrgyz Republic	44.2 [37.9, 50.7]	50.1 [43.1, 57.1]	0.882 [0.758, 1.026]	44.9 [40.3, 49.6]	42.0 [37.1, 47.1]	1.068 [0.942, 1.211]	1.211 [0.995, 1.474]
Tajikistan	67.9 [63.0, 72.5]	61.1 [55.0, 66.9]	1.112 [1.015, 1.217]	65.8 [61.6, 69.7]	61.7 [57.1, 66.2]	1.066 [1.003, 1.132]	0.959 [0.860, 1.069]
Latin America and Caribbean	41.8 [39.9, 43.7]	56.6 [54.1, 59.0]	0.739 [0.701, 0.778]	44.2 [42.5, 46.0]	59.0 [56.9, 61.1]	0.749 [0.719, 0.781]	1.015 [0.949, 1.084]
Dominican Republic	36.3 [32.8, 39.9]	33.5 [28.8, 38.5]	1.084 [0.928, 1.266]	35.0 [30.9, 39.3]	34.5 [30.0, 39.2]	1.015 [0.852, 1.209]	0.936 [0.741, 1.183]
Guatemala	36.3 [33.3, 39.5]	59.8 [56.1, 63.5]	0.607 [0.557, 0.662]	43.0 [40.3, 45.7]	61.8 [58.7, 64.9]	0.696 [0.652, 0.743]	1.146 [1.028, 1.277]
Haiti	37.5 [33.0, 42.2]	59.2 [53.5, 64.7]	0.633 [0.557, 0.719]	34.8 [31.4, 38.4]	52.2 [47.5, 56.9]	0.667 [0.601, 0.740]	1.053 [0.894, 1.241]
Honduras	62.8 [59.6, 66.0]	79.0 [75.7, 82.0]	0.795 [0.750, 0.844]	68.4 [66.1, 70.6]	81.7 [79.3, 83.8]	0.837 [0.807, 0.868]	1.052 [0.982, 1.128]
Middle East and North Africa	25.5 [23.3, 27.8]	28.1 [25.6, 30.6]	0.910 [0.838, 0.988]	24.3 [22.8, 25.9]	27.9 [26.2, 29.6]	0.873 [0.819, 0.930]	0.960 [0.865, 1.065]
Egypt	16.0 [14.1, 18.0]	19.2 [16.9, 21.9]	0.830 [0.719, 0.958]	14.5 [13.2, 16.0]	20.6 [18.8, 22.4]	0.707 [0.631, 0.792]	0.852 [0.709, 1.023]
Jordan	21.1 [16.9, 25.9]	53.9 [47.5, 60.1]	0.391 [0.311, 0.492]	10.7 [8.4, 13.6]	49.5 [42.8, 56.2]	0.217 [0.169, 0.279]	0.555 [0.395, 0.779]
Yemen	59.9 [55.8, 63.8]	56.4 [51.1, 61.5]	1.062 [0.987, 1.142]	53.4 [51.0, 55.8]	50.5 [47.7, 53.3]	1.057 [1.011, 1.105]	0.996 [0.914, 1.084]
South Asia	61.9 [60.4, 63.3]	63.3 [61.7, 65.0]	0.977 [0.957, 0.998]	60.9 [60.1, 61.8]	60.7 [59.8, 61.6]	1.004 [0.992, 1.017]	1.028 [1.004, 1.053]
Afghanistan	50.3 [45.1, 55.5]	50.8 [45.3, 56.3]	0.990 [0.909, 1.079]	50.9 [45.1, 56.6]	49.4 [42.8, 55.9]	1.031 [0.985, 1.079]	1.041 [0.944, 1.147]

	Urban			Rural			
	Under two	Over two	Over two/Under two	Under two	Over two	Over two/Under two	
	%	%	Relative difference	%	%	Relative difference	Interaction term
Bangladesh	62.2 [57.5, 66.7]	50.1 [44.6, 55.7]	1.241 [1.115, 1.381]	64.5 [61.3, 67.5]	52.7 [49.2, 56.2]	1.223 [1.152, 1.299]	0.986 [0.872, 1.114]
India	60.3 [59.1, 61.6]	63.8 [62.4, 65.2]	0.945 [0.926, 0.966]	56.6 [55.9, 57.2]	59.4 [58.7, 60.0]	0.953 [0.942, 0.964]	1.008 [0.984, 1.033]
Maldives	64.0 [56.4, 71.0]	55.2 [42.7, 67.0]	1.160 [0.928, 1.450]	77.1 [74.2, 79.7]	55.3 [51.0, 59.5]	1.395 [1.287, 1.511]	1.202 [0.951, 1.521]
Nepal	89.7 [85.5, 92.7]	71.5 [64.6, 77.5]	1.254 [1.159, 1.358]	93.4 [91.9, 94.7]	76.4 [72.8, 79.6]	1.223 [1.171, 1.278]	0.975 [0.891, 1.067]
Pakistan	69.5 [63.4, 75.0]	69.1 [61.6, 75.6]	1.006 [0.943, 1.074]	75.7 [71.9, 79.1]	72.9 [68.9, 76.7]	1.038 [0.998, 1.078]	1.031 [0.956, 1.112]
West and Central Africa	63.6 [61.0, 66.0]	65.0 [62.5, 67.4]	0.978 [0.958, 0.999]	51.9 [50.1, 53.6]	50.5 [48.8, 52.3]	1.026 [1.006, 1.047]	1.049 [1.020, 1.080]
Benin	54.8 [51.0, 58.5]	56.8 [52.8, 60.8]	0.964 [0.910, 1.021]	46.1 [43.6, 48.5]	47.0 [44.0, 50.0]	0.980 [0.929, 1.033]	1.016 [0.940, 1.099]
Burkina Faso	64.3 [59.7, 68.7]	71.4 [66.7, 75.6]	0.901 [0.843, 0.963]	61.2 [58.0, 64.4]	64.9 [61.7, 67.9]	0.944 [0.913, 0.976]	1.048 [0.973, 1.129]
Cameroon	63.1 [59.9, 66.3]	72.7 [67.9, 77.0]	0.869 [0.808, 0.934]	71.7 [67.8, 75.3]	79.8 [76.4, 82.8]	0.899 [0.861, 0.939]	1.035 [0.951, 1.126]
Chad	54.2 [49.1, 59.3]	48.1 [42.1, 54.1]	1.128 [1.028, 1.237]	44.5 [41.2, 47.8]	41.9 [38.9, 45.1]	1.061 [1.008, 1.118]	0.941 [0.847, 1.046]
Congo	68.0 [63.5, 72.2]	68.6 [62.4, 74.2]	0.991 [0.924, 1.063]	63.1 [58.7, 67.3]	60.8 [56.3, 65.2]	1.037 [0.994, 1.083]	1.047 [0.964, 1.136]
Congo Democratic Republic	84.6 [81.8, 87.1]	82.2 [78.9, 85.1]	1.029 [1.001, 1.057]	67.1 [63.5, 70.6]	63.3 [59.7, 66.7]	1.061 [1.020, 1.103]	1.031 [0.983, 1.081]
Cote D'Ivoire	73.9 [69.2, 78.1]	73.1 [67.7, 77.9]	1.011 [0.946, 1.080]	56.8 [51.8, 61.6]	55.1 [50.5, 59.6]	1.031 [0.966, 1.100]	1.019 [0.929, 1.118]
Gabon	57.2 [51.6, 62.6]	55.5 [51.2, 59.7]	1.030 [0.931, 1.140]	67.4 [62.7, 71.8]	60.4 [54.6, 65.8]	1.117 [1.037, 1.204]	1.084 [0.956, 1.229]
Gambia	59.3 [53.8, 64.6]	73.9 [68.3, 78.9]	0.802 [0.732, 0.880]	63.7 [57.6, 69.4]	76.6 [72.4, 80.3]	0.832 [0.766, 0.903]	1.037 [0.917, 1.172]
Ghana	58.6 [53.8, 63.2]	69.8 [64.3, 74.7]	0.840 [0.760, 0.928]	63.1 [58.4, 67.5]	70.3 [62.6, 77.0]	0.898 [0.822, 0.981]	1.069 [0.936, 1.222]
Guinea	48.7 [42.8, 54.5]	53.6 [46.2, 60.9]	0.907 [0.808, 1.018]	38.0 [33.3, 42.9]	34.0 [29.5, 38.8]	1.119 [1.029, 1.216]	1.233 [1.070, 1.422]
Liberia	66.4 [59.2, 72.8]	68.2 [61.7, 74.0]	0.974 [0.904, 1.048]	56.2 [51.5, 60.7]	57.0 [52.4, 61.4]	0.986 [0.925, 1.051]	1.013 [0.919, 1.116]
Mali	73.9 [69.4, 78.0]	70.0 [65.5, 74.1]	1.056 [0.997, 1.118]	58.9 [55.4, 62.2]	55.3 [51.5, 59.0]	1.065 [1.012, 1.121]	1.009 [0.934, 1.089]
Niger	71.8 [67.2, 76.0]	72.8 [67.8, 77.3]	0.986 [0.930, 1.044]	59.1 [55.8, 62.3]	59.5 [55.7, 63.2]	0.993 [0.951, 1.037]	1.007 [0.937, 1.083]
Nigeria	53.2 [48.7, 57.6]	54.4 [50.0, 58.8]	0.976 [0.934, 1.021]	35.4 [32.3, 38.6]	33.9 [31.1, 36.8]	1.046 [0.995, 1.099]	1.071 [1.002, 1.145]
Senegal	83.8 [80.1, 86.9]	82.8 [78.9, 86.1]	1.012 [0.972, 1.054]	79.2 [76.0, 82.1]	74.6 [71.4, 77.5]	1.062 [1.030, 1.096]	1.049 [0.997, 1.104]
Sierra Leone	90.2 [86.5, 92.9]	87.7 [83.5, 90.9]	1.029 [0.990, 1.069]	86.6 [83.9, 88.9]	83.7 [80.8, 86.2]	1.035 [1.005, 1.065]	1.006 [0.958, 1.056]
Togo	83.8 [79.9, 87.0]	80.8 [76.5, 84.4]	1.037 [0.996, 1.080]	83.4 [79.8, 86.5]	81.3 [77.9, 84.3]	1.025 [0.996, 1.056]	0.988 [0.940, 1.039]

Notes: 95% confidence intervals are shown in brackets.

Figure S1. Percentage of children 6–23 months old receiving minimum dietary diversity: by sex (showing relative differences)

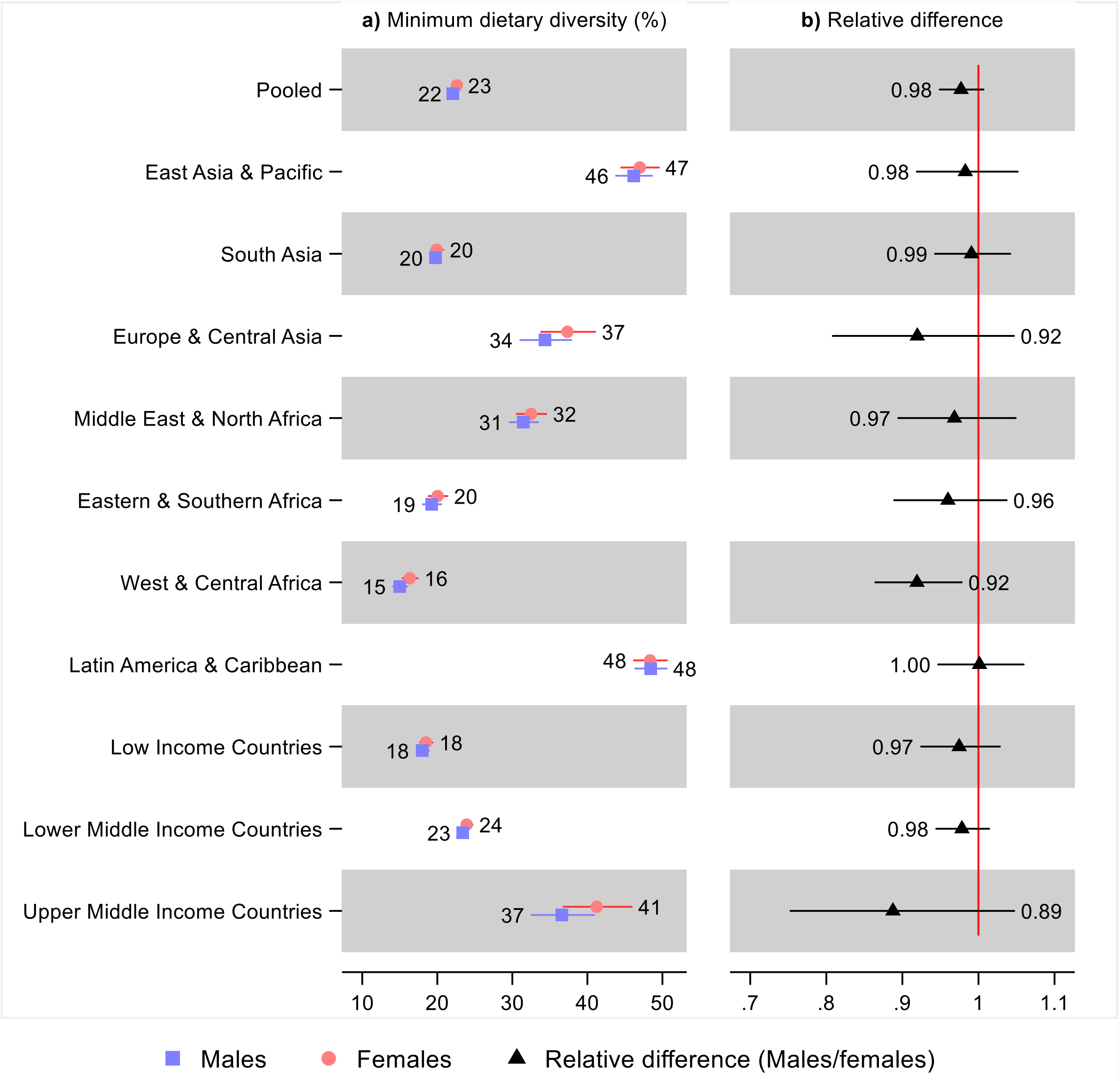

Notes: 95% confidence intervals are shown. See Table S5 in the Supplement for tabulated estimates and confidence intervals.

Figure S2. Percentage of children 6–23 months old receiving vitamin-A-rich foods: by sex (showing relative differences)

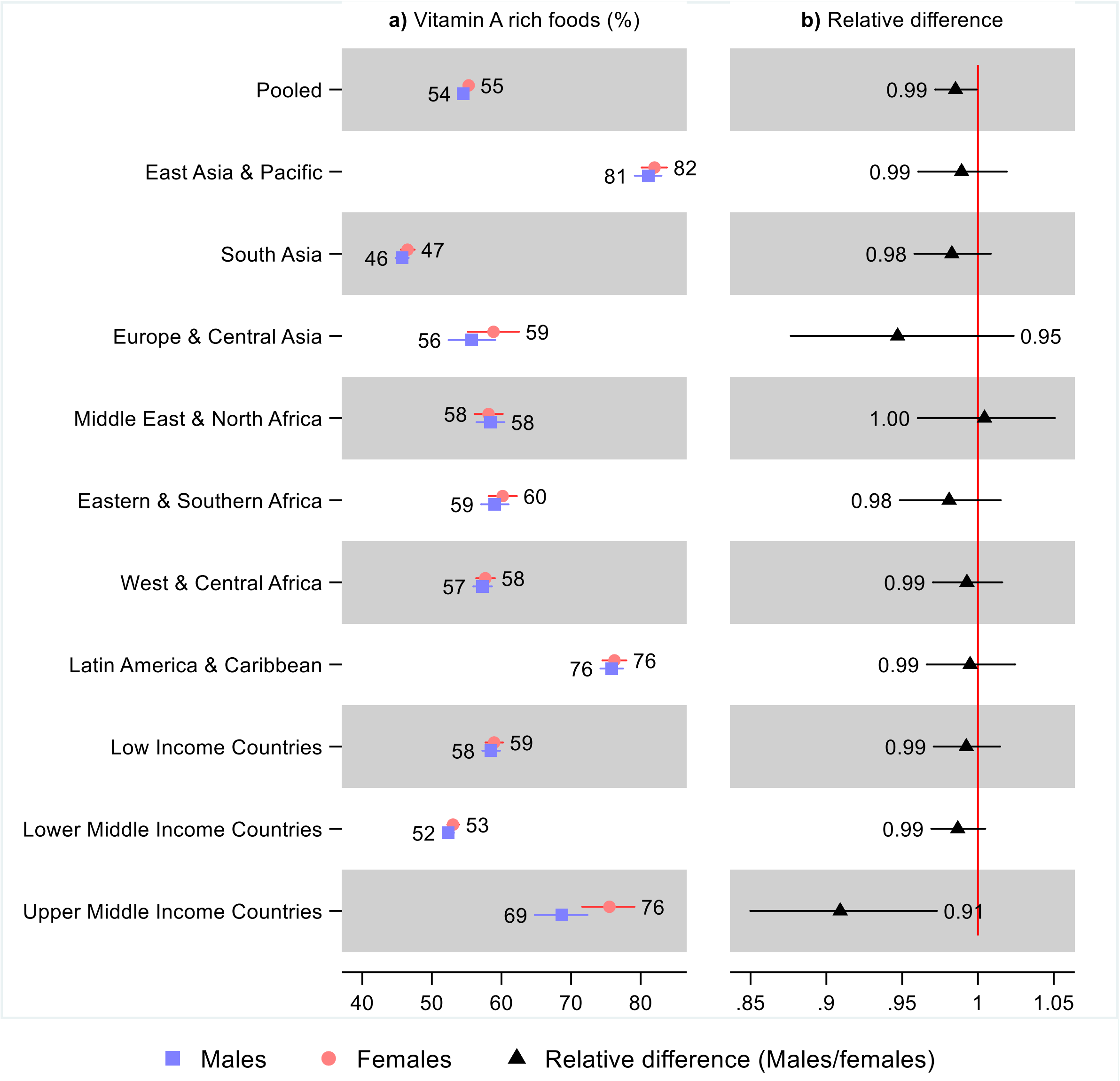

Notes: 95% confidence intervals are shown. See Table S6 in the Supplement for tabulated estimates and confidence intervals.

Figure S3. Percentage of children 6–23 months old receiving vitamin A supplementation: by sex (showing relative differences)

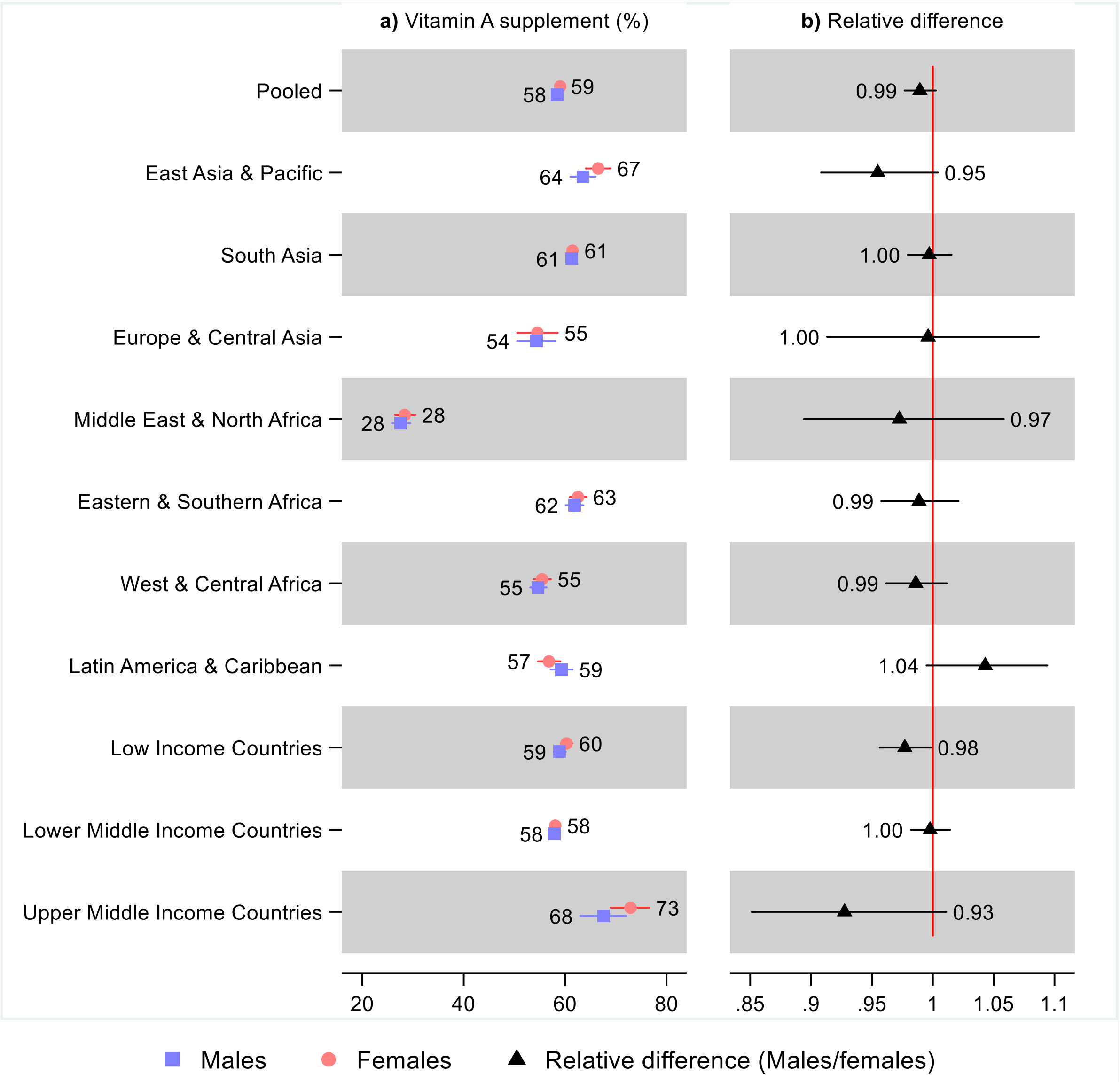

Notes: 95% confidence intervals are shown. See Table S7 in the Supplement for tabulated estimates and confidence intervals.

Figure S4. Percentage of children 6–23 months old receiving either vitamin-A-rich foods or vitamin A supplementation: by sex (showing relative differences)

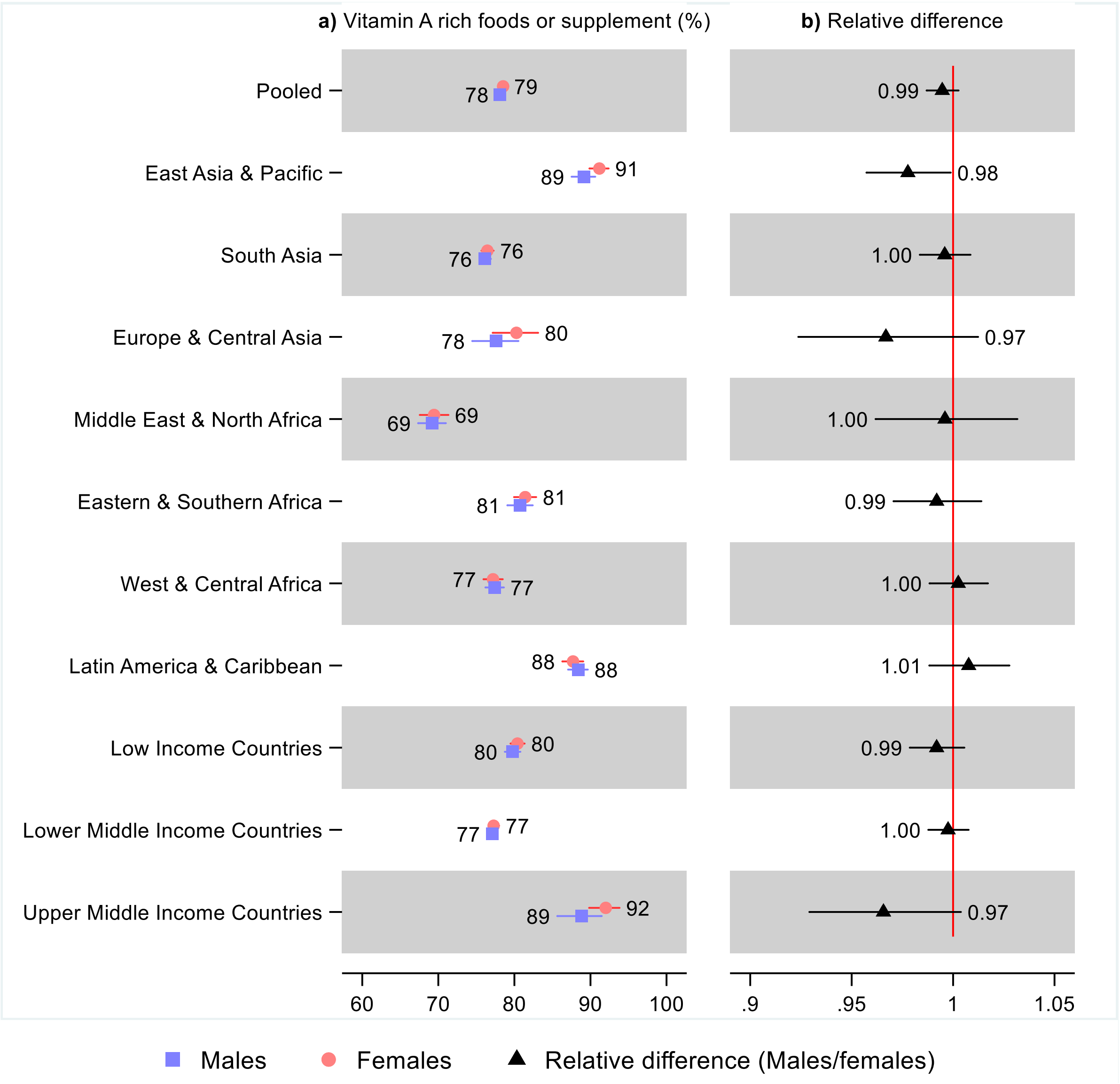

Notes: 95% confidence intervals are shown. See Table S8 in the Supplement for tabulated estimates and confidence intervals.

Figure S5. Percentage of children 6–23 months old receiving minimum dietary diversity: by sex and country (showing relative differences)

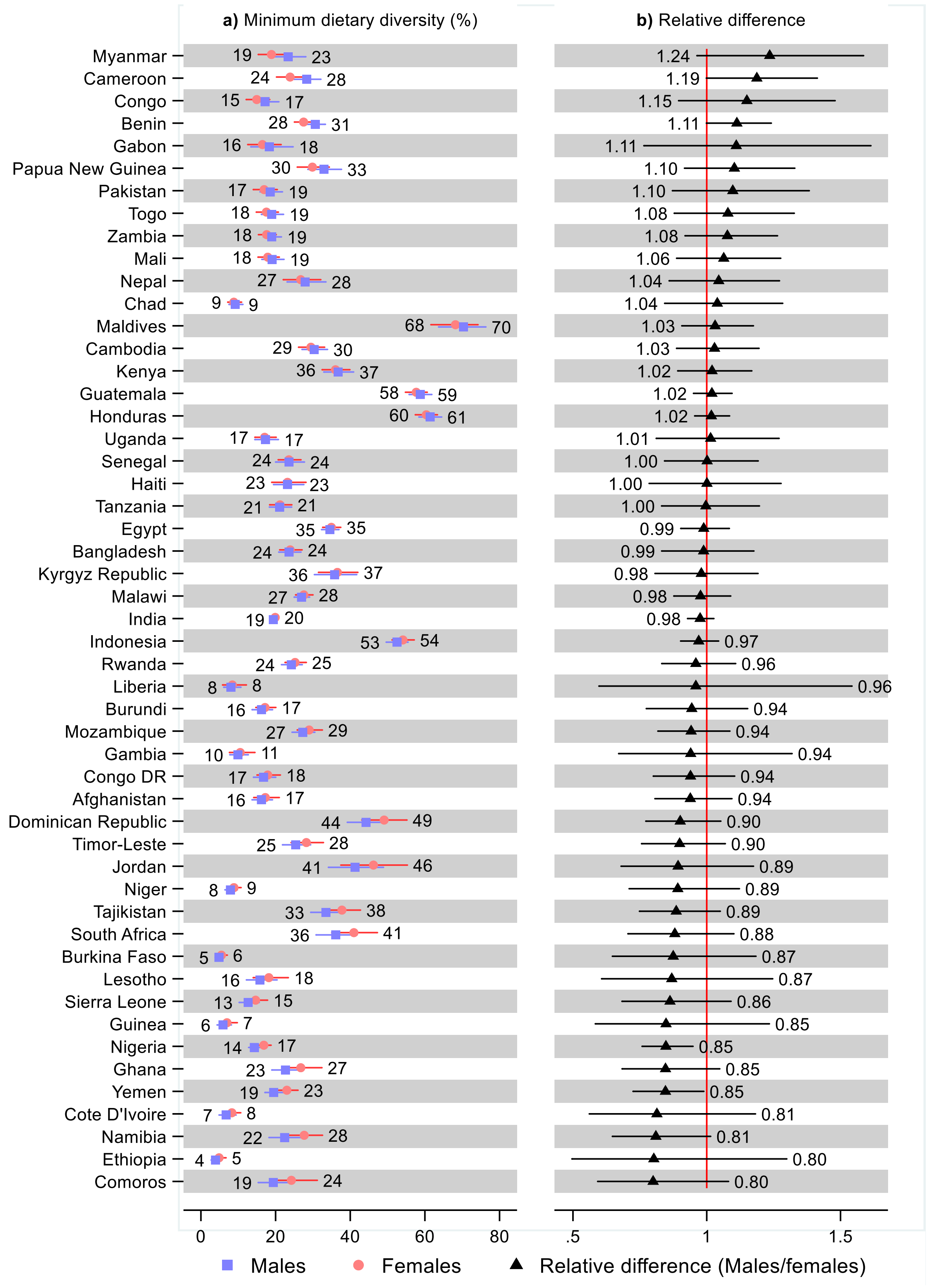

Notes: 95% confidence intervals are shown. Countries are ordered from largest to smallest relative sex difference in percentage of children receiving minimum dietary diversity. See Table S5 in the Supplement for tabulated estimates and confidence intervals.

Figure S6. Percentage of children 6–23 months old receiving vitamin-A-rich foods: by sex and country (showing relative differences)

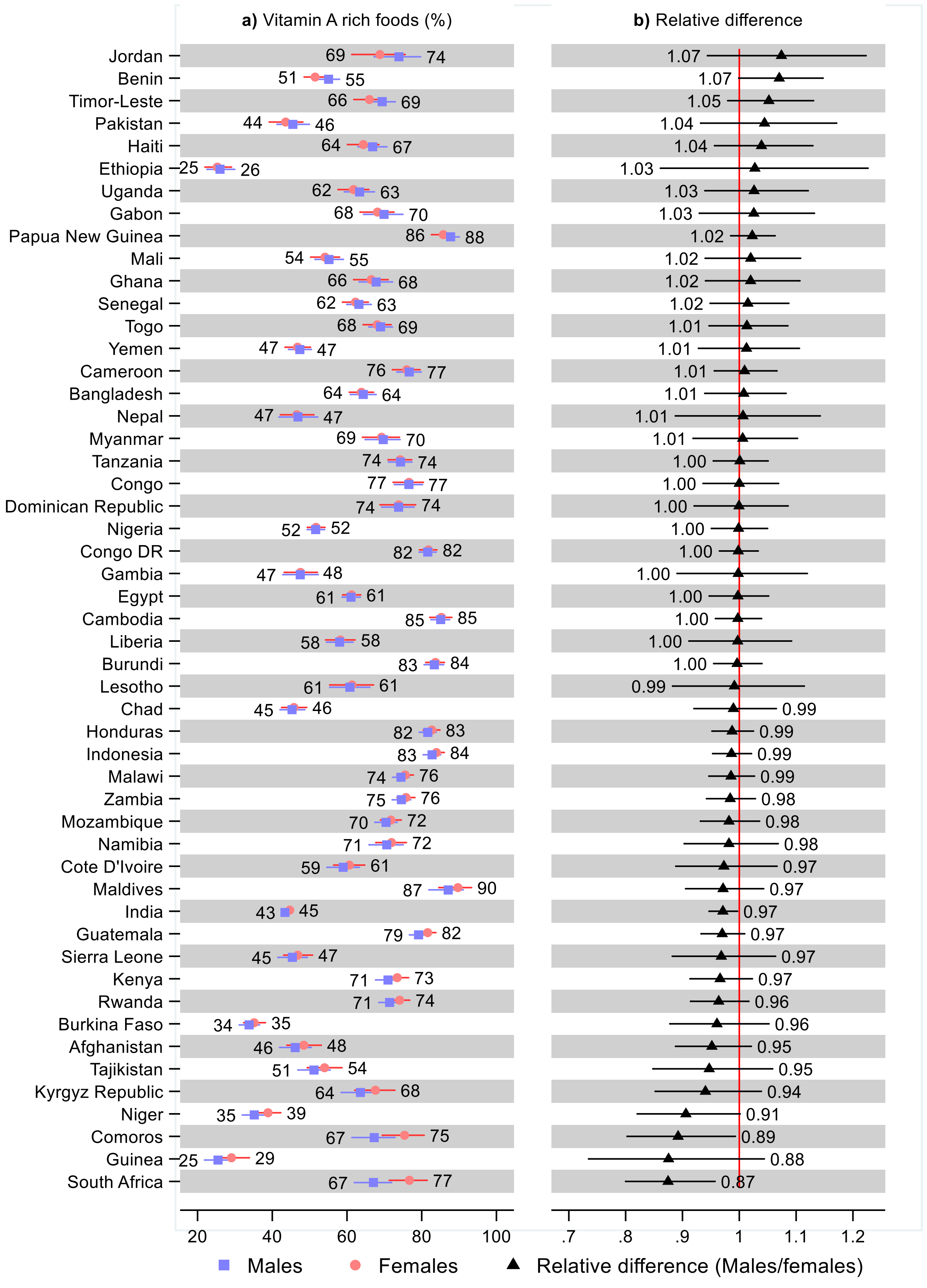

Notes: 95% confidence intervals are shown. Countries are ordered from largest to smallest relative sex difference in percentage of children receiving vitamin-A-rich foods. See Table S6 in the Supplement for tabulated estimates and confidence intervals.

Figure S7. Percentage of children 6–23 months old receiving vitamin A supplementation: by sex and country (showing relative differences)

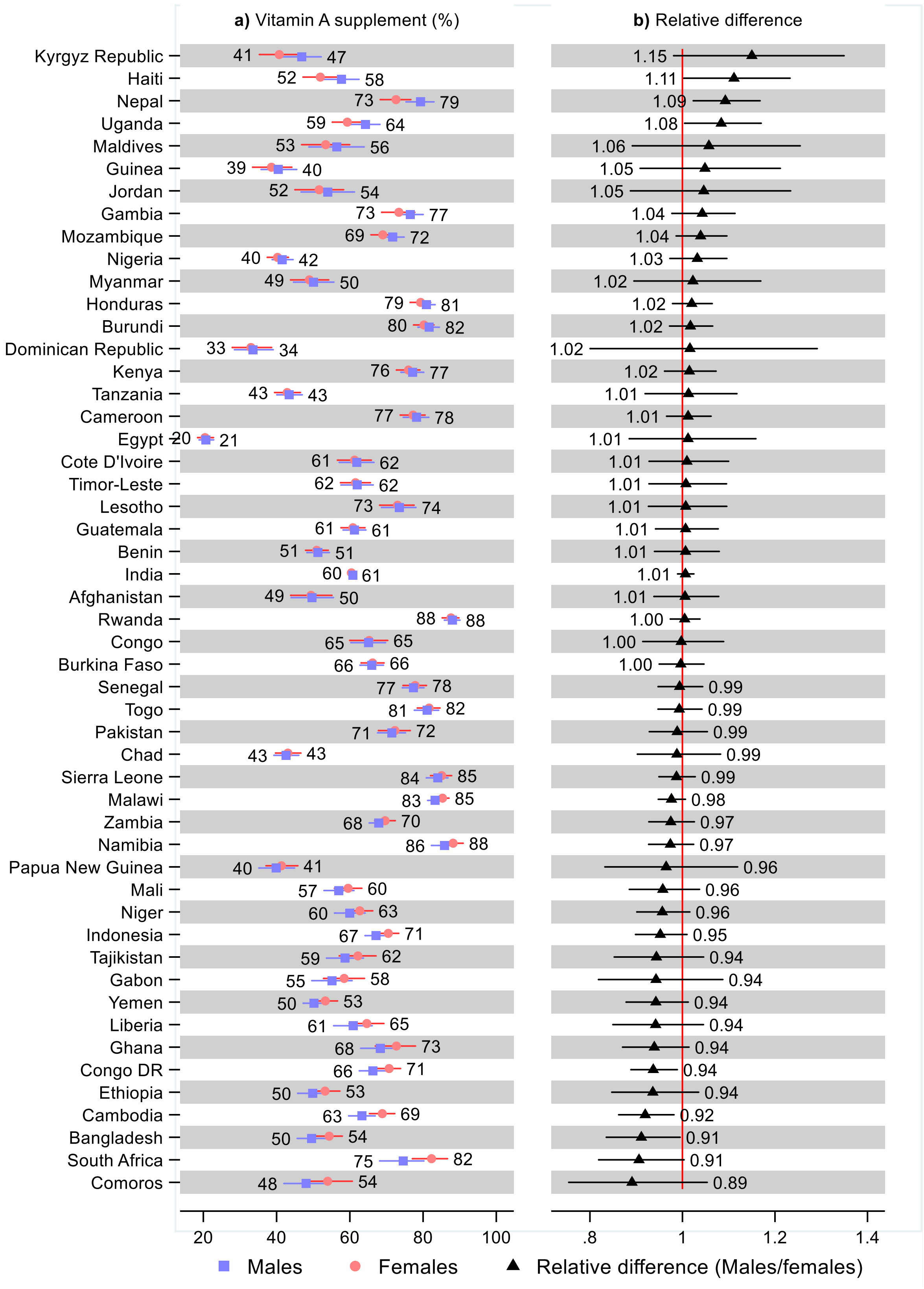

Notes: 95% confidence intervals are shown. Countries are ordered from largest to smallest relative sex difference in percentage of children receiving vitamin A supplementation. See Table S7 in the Supplement for tabulated estimates and confidence intervals.

Figure S8. Percentage of children 6–23 months old receiving either vitamin-A-rich foods or vitamin A supplementation: by sex and country (showing relative differences)

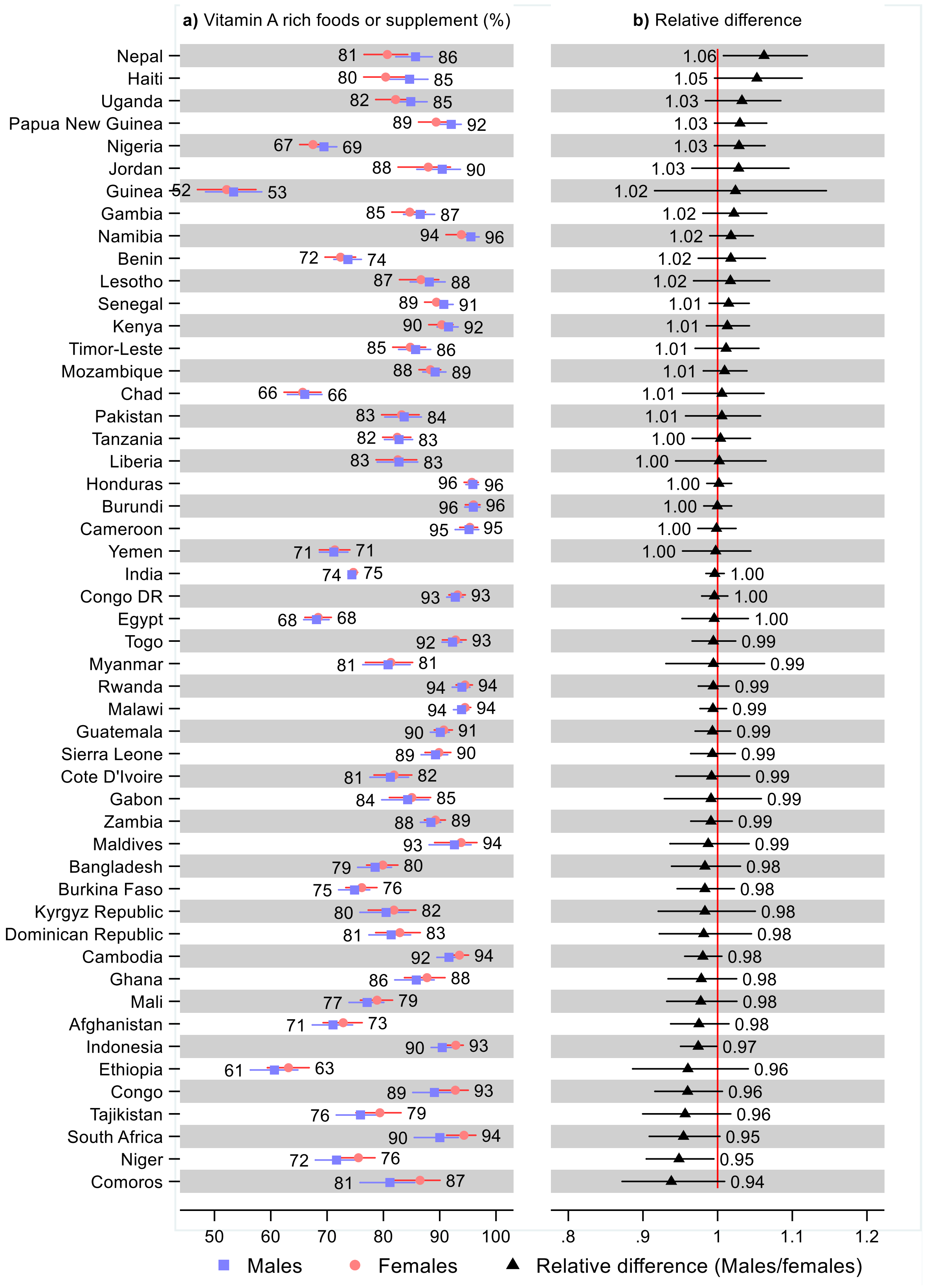

Notes: 95% confidence intervals are shown. Countries are ordered from largest to smallest relative sex difference in percentage of children receiving either vitamin-A-rich foods or vitamin A supplementation. See Table S8 in the Supplement for tabulated estimates and confidence intervals.

Figure S9. Percentage of children 6–23 months old receiving minimum dietary diversity: by living standards (showing relative differences)

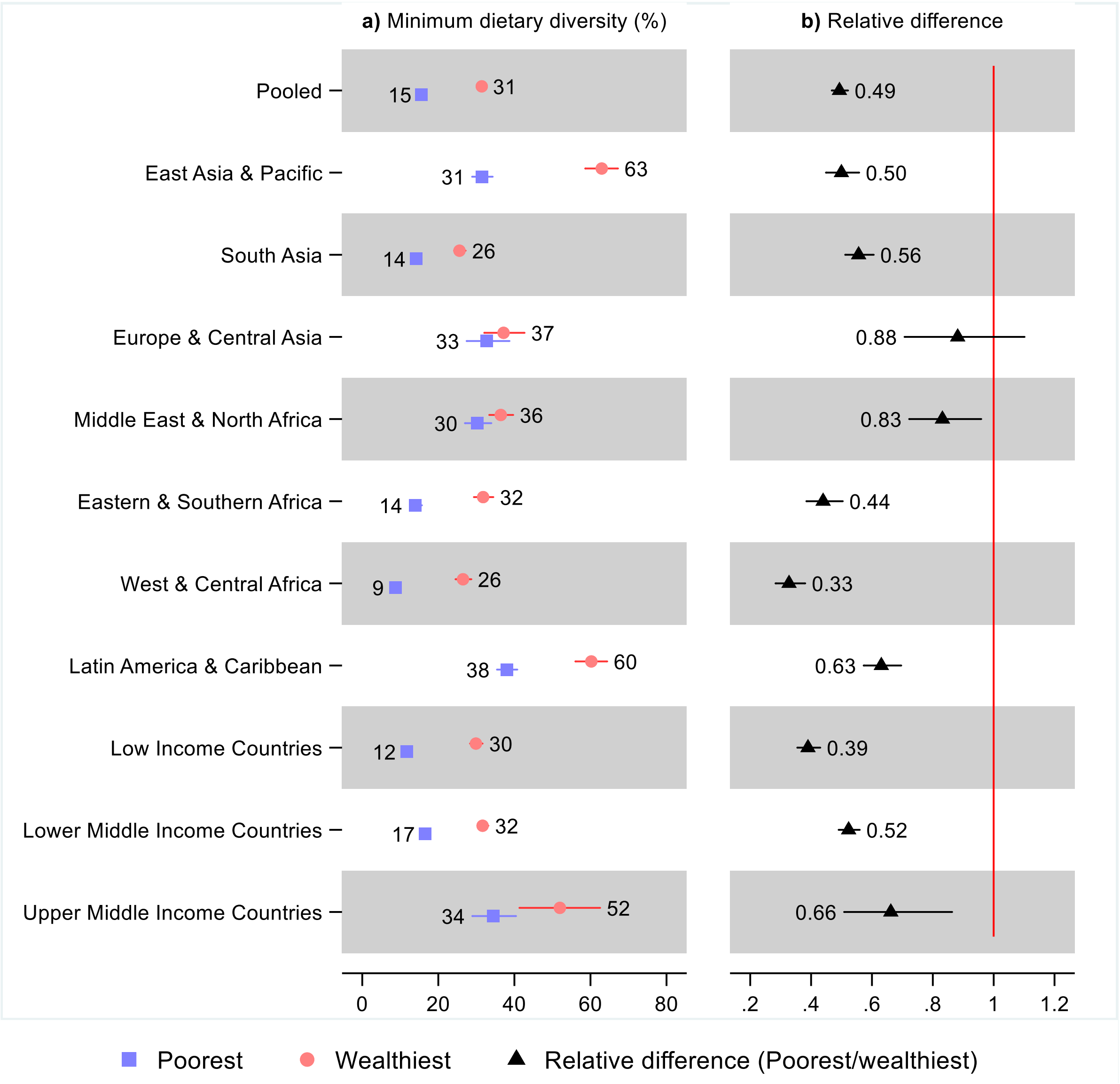

Notes: 95% confidence intervals are shown. See Table S9 in the Supplement for tabulated estimates and confidence intervals.

Figure S10. Percentage of children 6–23 months old receiving vitamin-A-rich foods: by living standards (showing relative differences)

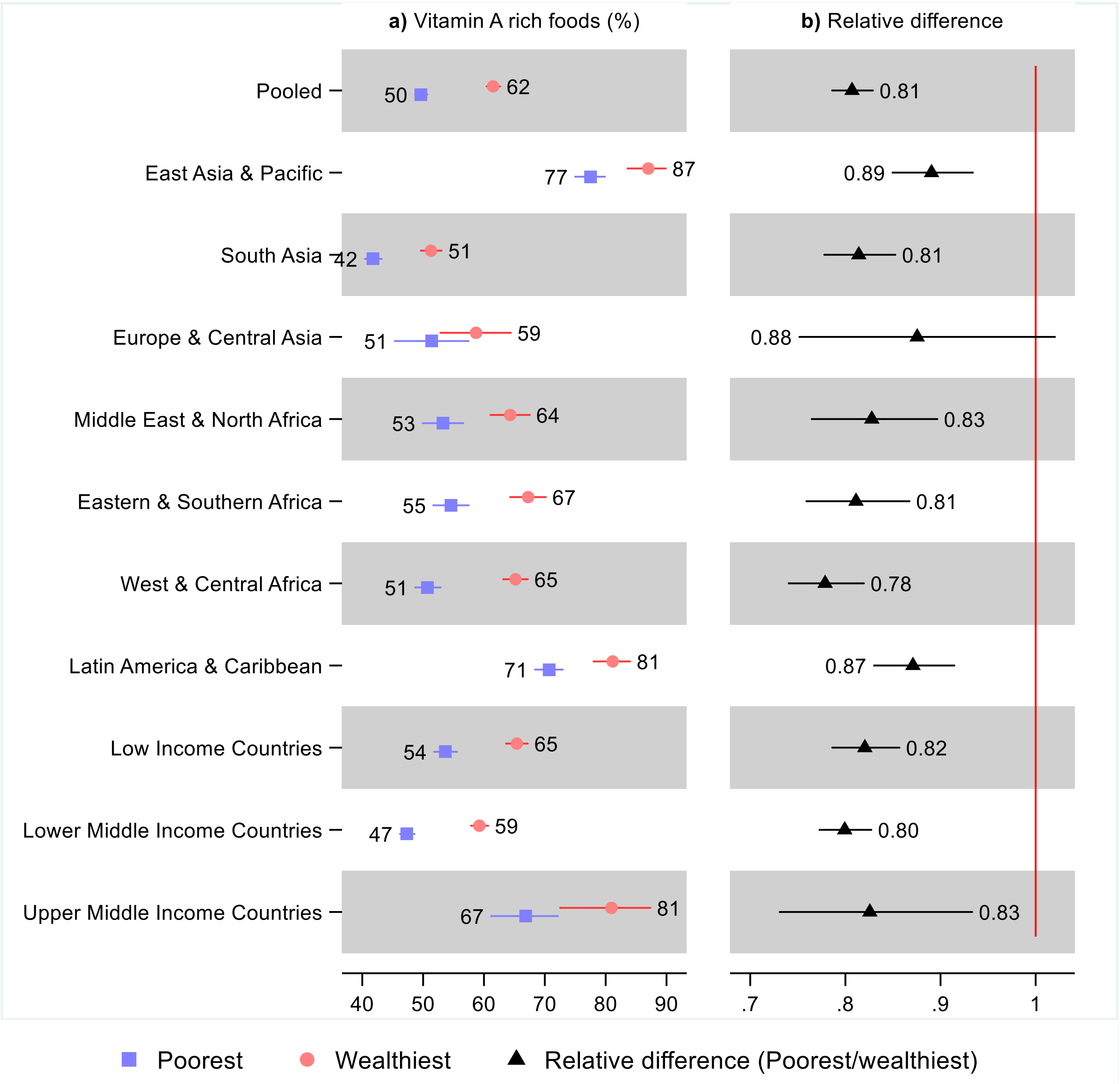

Notes: 95% confidence intervals are shown. See Table S10 in the Supplement for tabulated estimates and confidence intervals.

Figure S11. Percentage of children 6–23 months old receiving vitamin A supplementation: by living standards (showing relative differences)

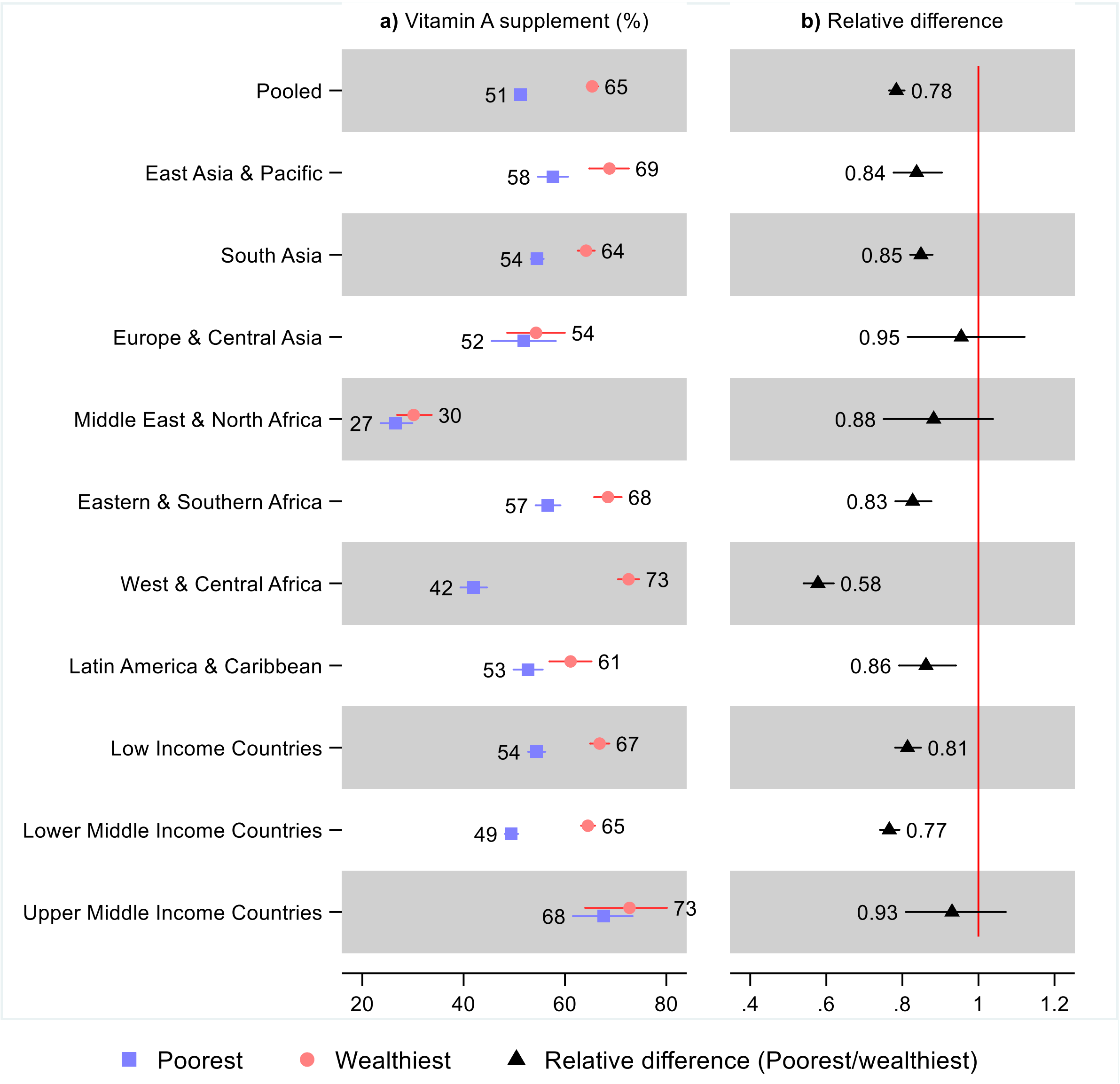

Notes: 95% confidence intervals are shown. See Table S11 in the Supplement for tabulated estimates and confidence intervals.

Figure S12. Percentage of children 6–23 months old receiving either vitamin-A-rich foods or vitamin A supplementation: by living standards (showing relative differences)

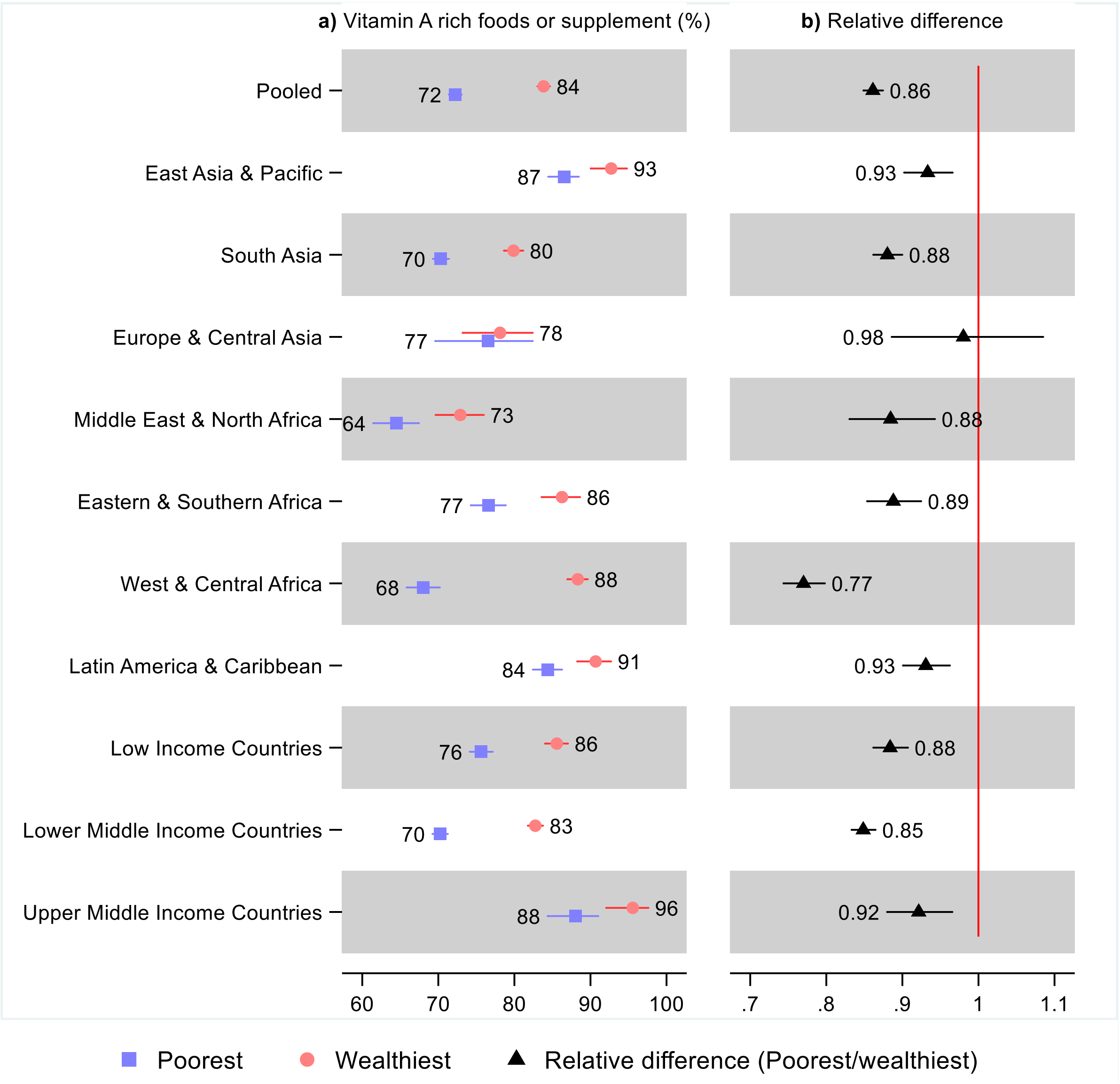

Notes: 95% confidence intervals are shown. See Table S12 in the Supplement for tabulated estimates and confidence intervals.

Figure S13. Percentage of children 6–23 months old receiving minimum dietary diversity: by living standards and country (showing relative differences)

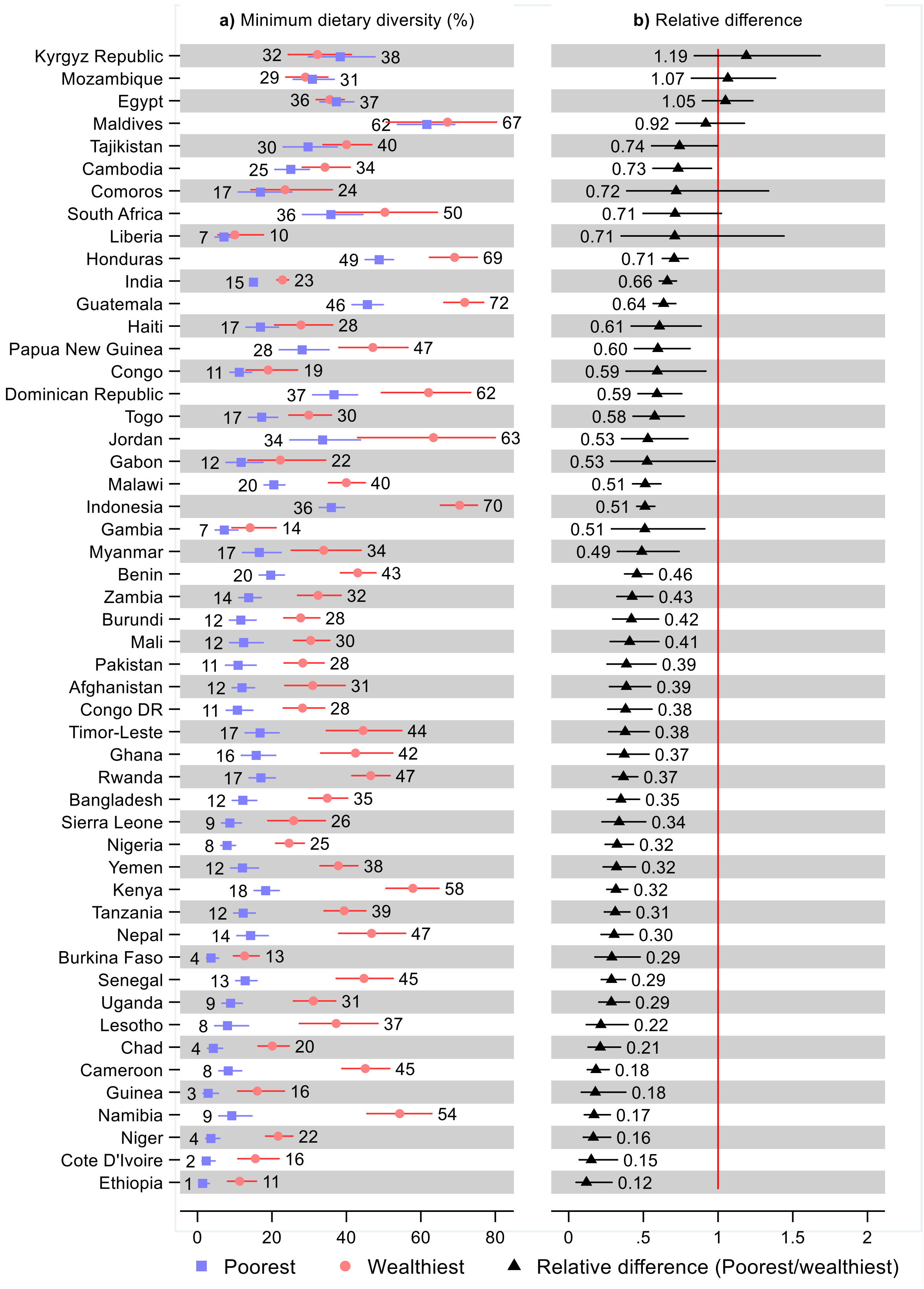

Notes: 95% confidence intervals are shown. Countries are ordered from largest to smallest relative living standards difference in percentage of children receiving minimum dietary diversity. See Table S9 in the Supplement for tabulated estimates and confidence intervals.

Figure S14. Percentage of children 6–23 months old receiving vitamin-A-rich foods: by living standards and country (showing relative differences)

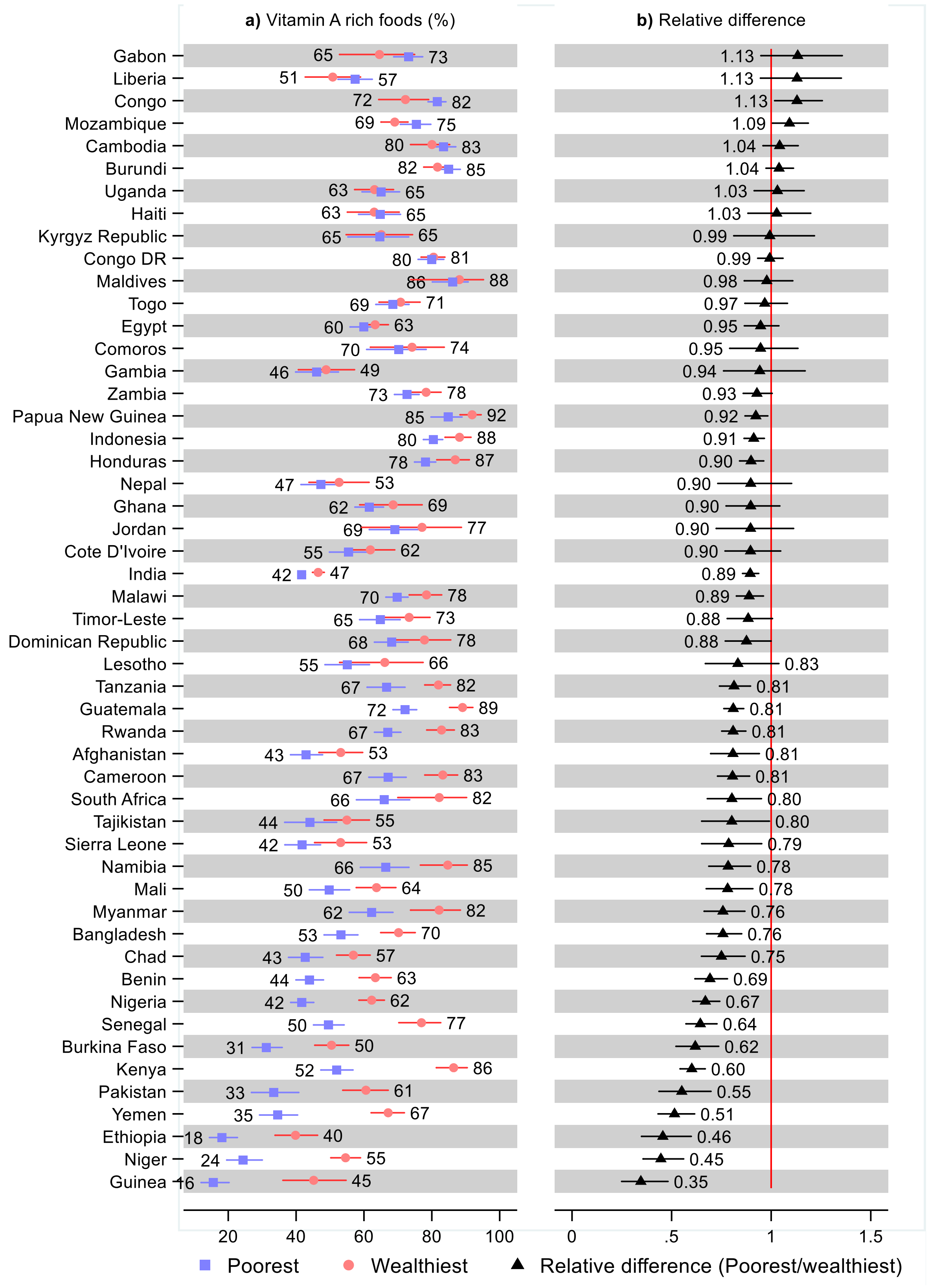

Notes: 95% confidence intervals are shown. Countries are ordered from largest to smallest relative living standards difference in percentage of children receiving vitamin-A-rich foods. See Table S10 in the Supplement for tabulated estimates and confidence intervals.

Figure S15. Percentage of children 6–23 months old receiving vitamin A supplementation: by living standards and country (showing relative differences)

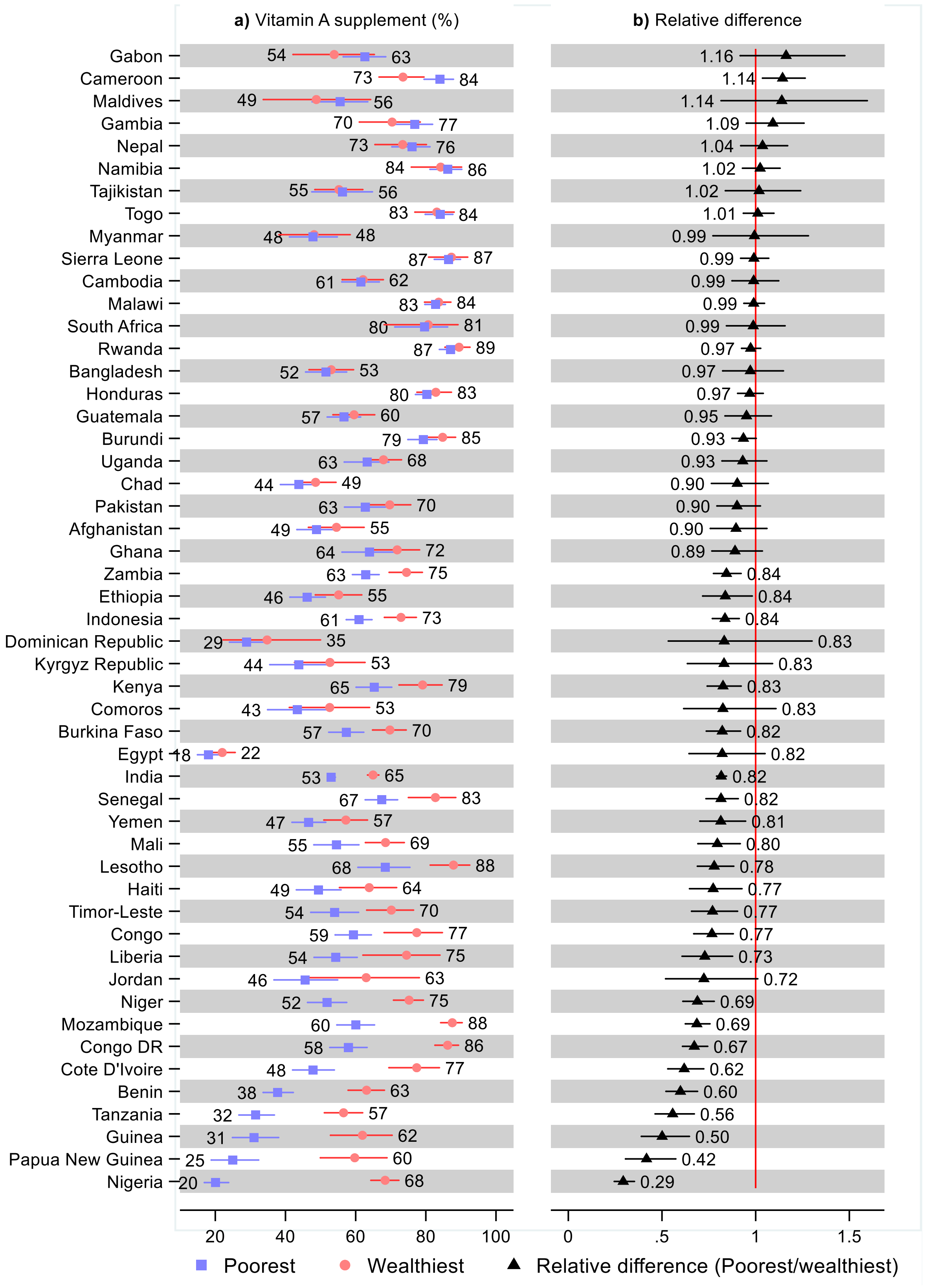

Notes: 95% confidence intervals are shown. Countries are ordered from largest to smallest relative living standards difference in percentage of children receiving vitamin A supplementation. See Table S11 in the Supplement for tabulated estimates and confidence intervals.

Figure S16. Percentage of children 6–23 months old receiving either vitamin-A-rich foods or vitamin A supplementation: by living standards and country (showing relative differences)

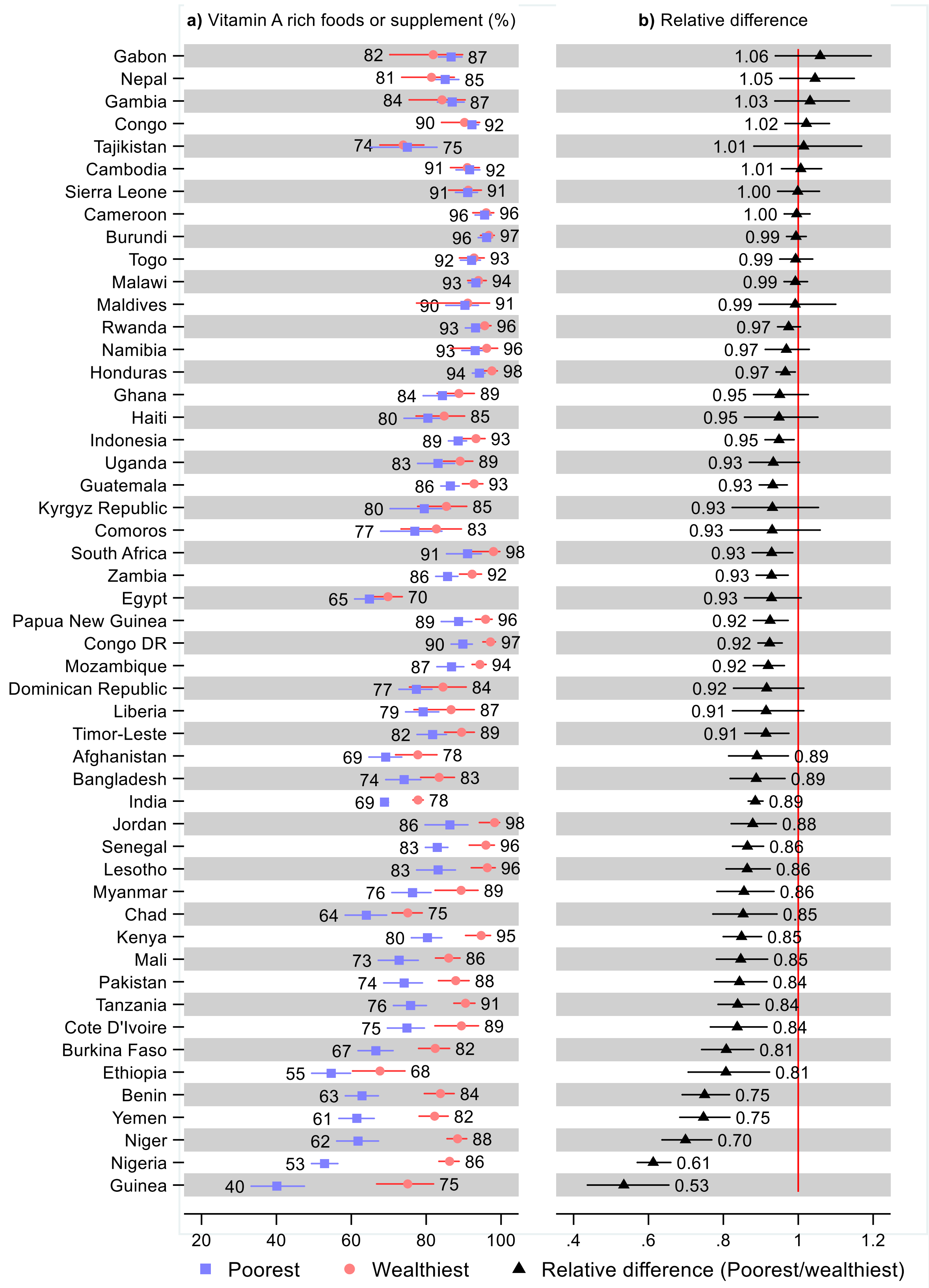

Notes: 95% confidence intervals are shown. Countries are ordered from largest to smallest relative living standards difference in percentage of children receiving either vitamin-A-rich foods or vitamin A supplementation. See Table S12 in the Supplement for tabulated estimates and confidence intervals.

Figure S17. Percentage of children 6–23 months old receiving minimum dietary diversity: by urban-rural residency (showing relative differences)

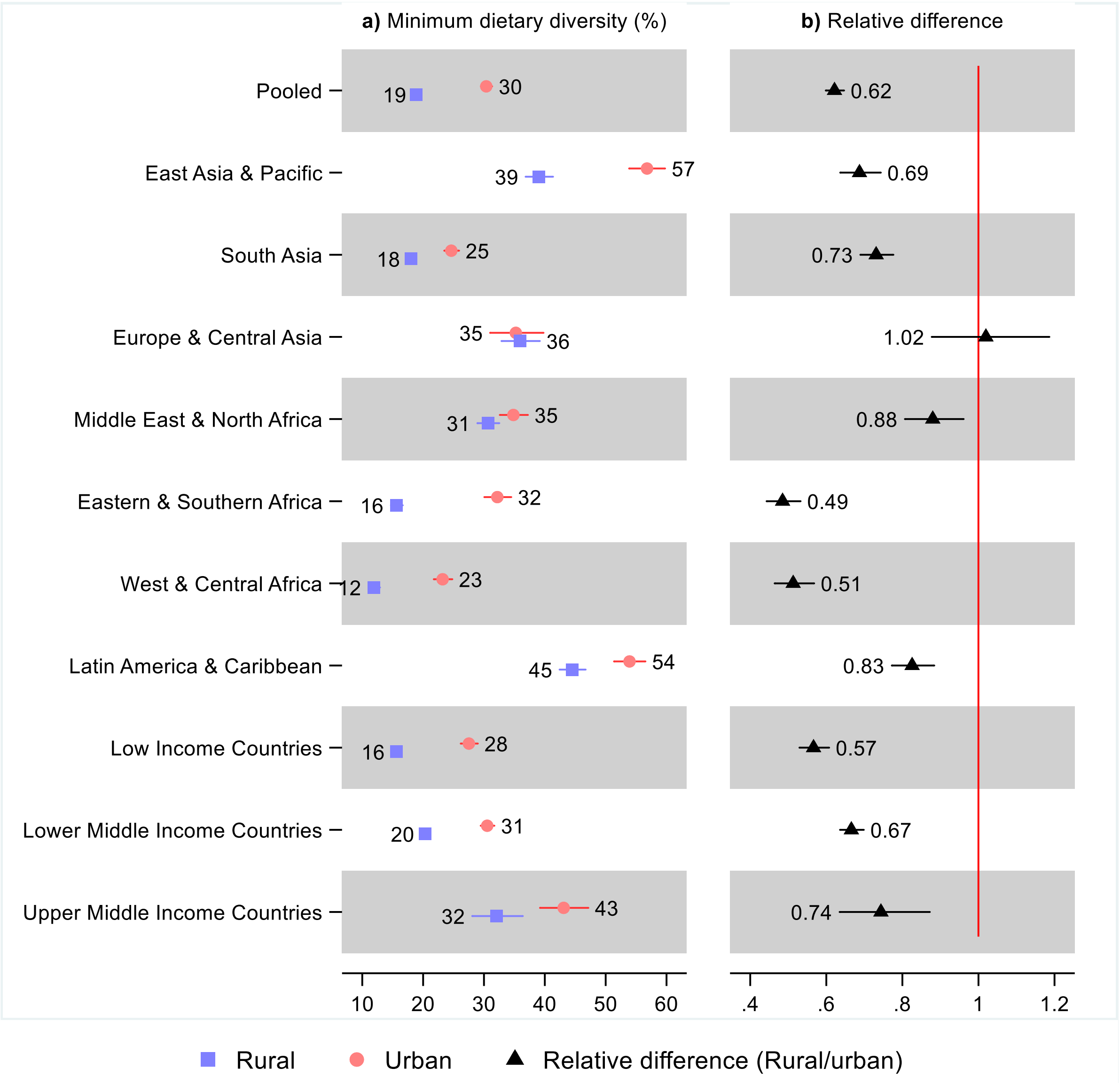

Notes: 95% confidence intervals are shown. See Table S13 in the Supplement for tabulated estimates and confidence intervals.

Figure S18. Percentage of children 6–23 months old receiving vitamin-A-rich foods: by urban-rural residency (showing relative differences)

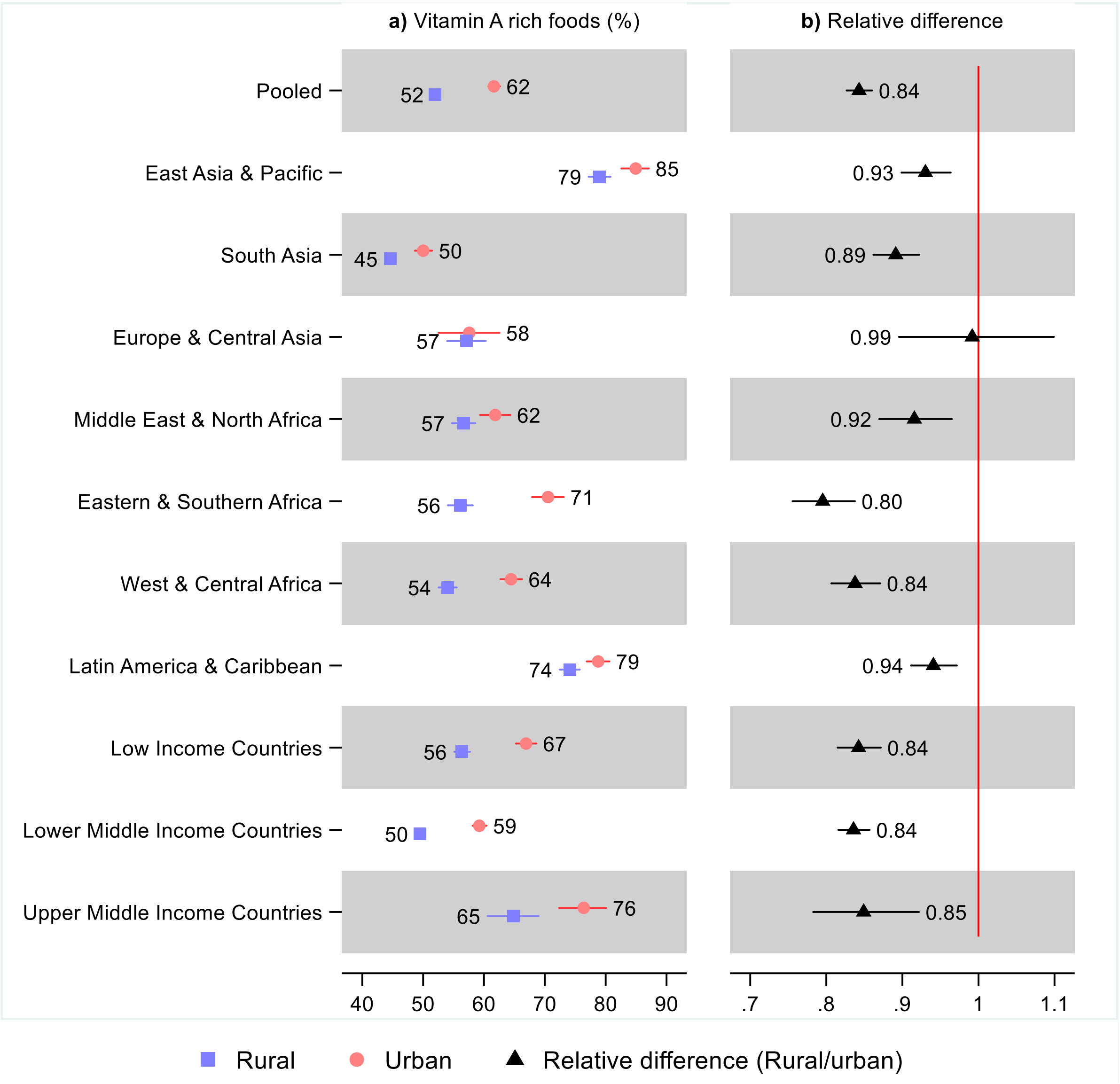

Notes: 95% confidence intervals are shown. See Table S14 in the Supplement for tabulated estimates and confidence intervals.

Figure S19. Percentage of children 6–23 months old receiving vitamin A supplementation: by urban-rural residency (showing relative differences)

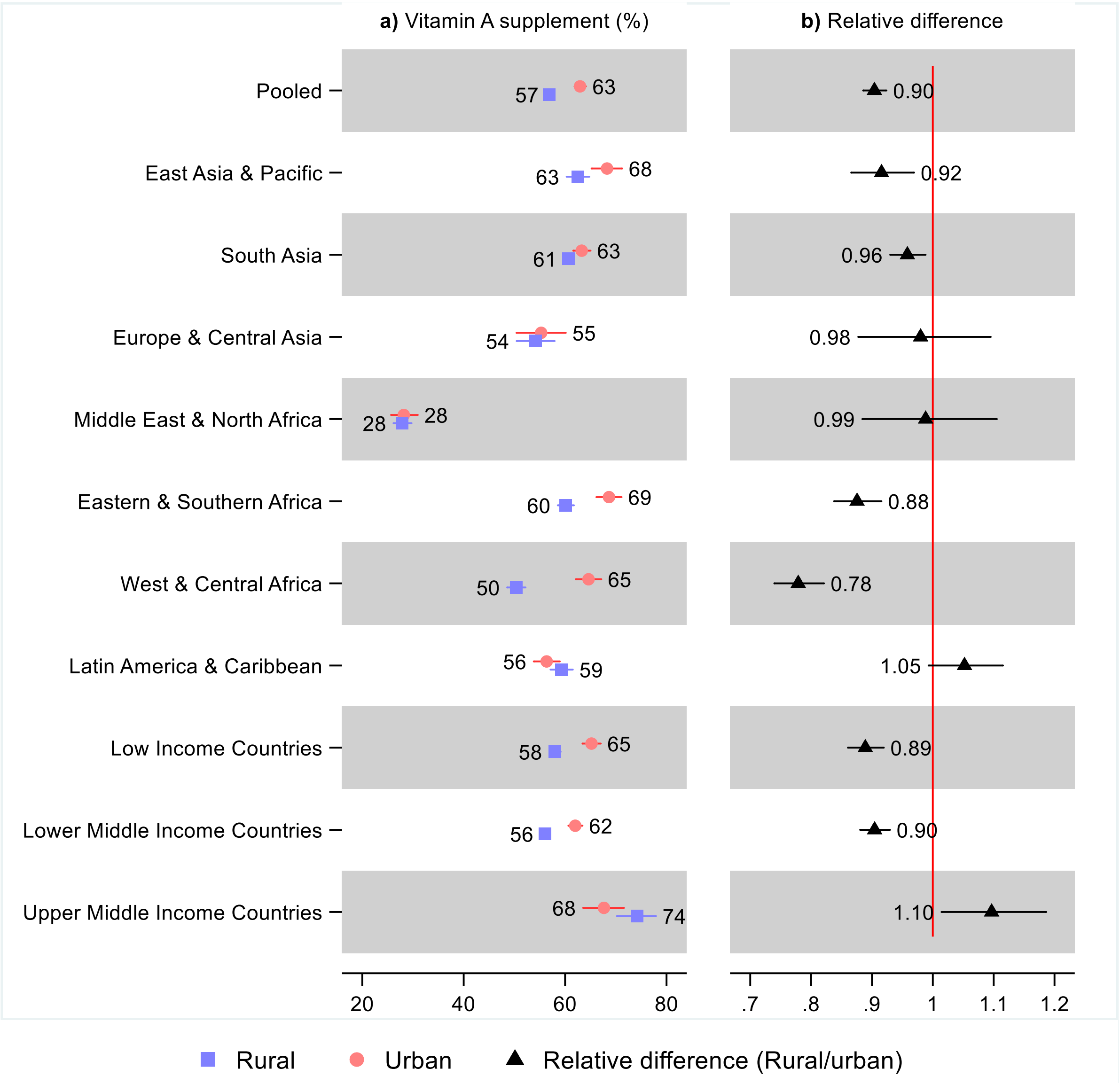

Notes: 95% confidence intervals are shown. See Table S15 in the Supplement for tabulated estimates and confidence intervals.

Figure S20. Percentage of children 6–23 months old receiving either vitamin-A-rich foods or vitamin A supplementation: by urban-rural residency (showing relative differences)

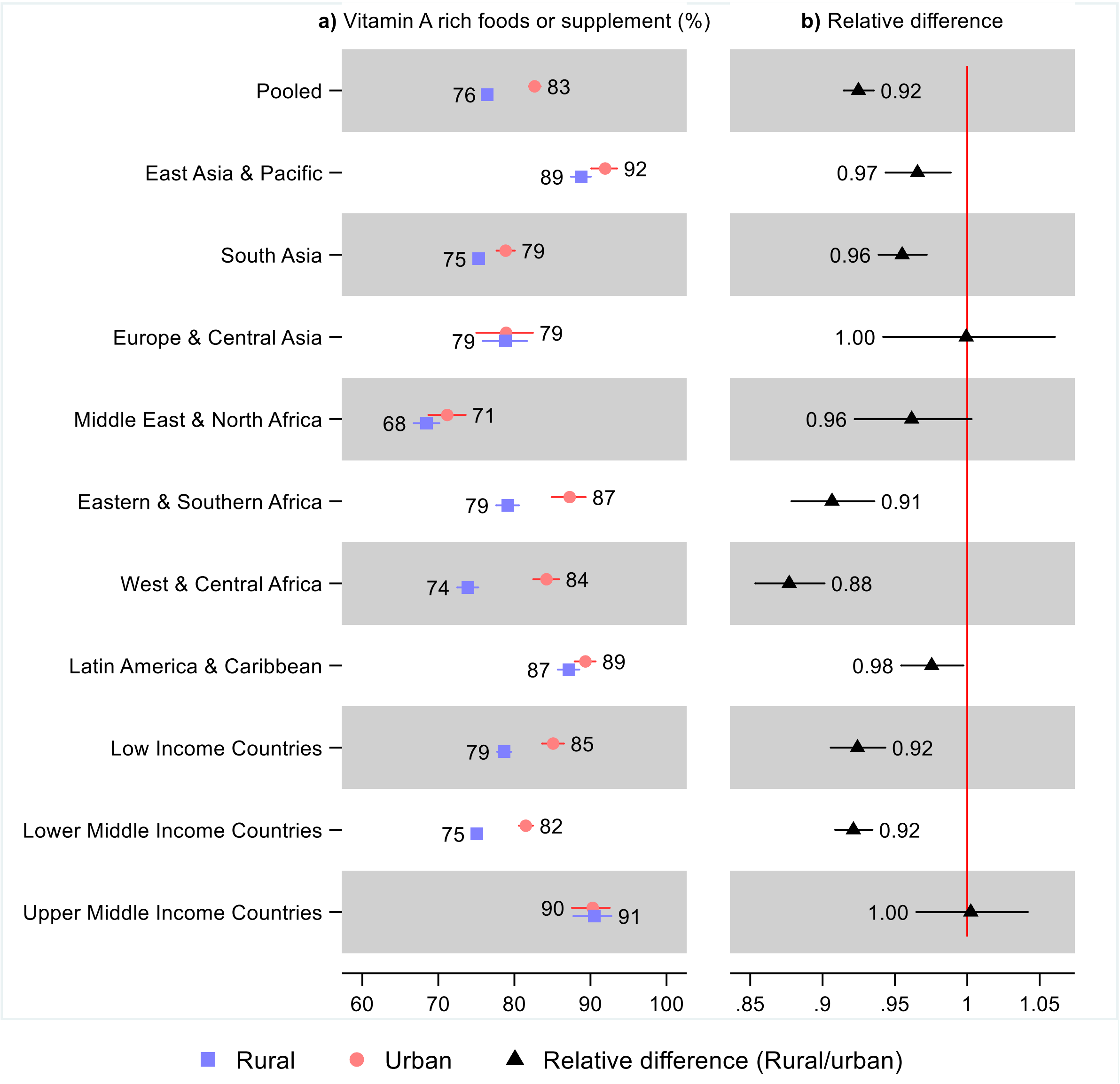

Notes: 95% confidence intervals are shown. See Table S16 in the Supplement for tabulated estimates and confidence intervals.

Figure S21. Percentage of children 6–23 months old receiving minimum dietary diversity: by urban-rural residency and country (showing relative differences)

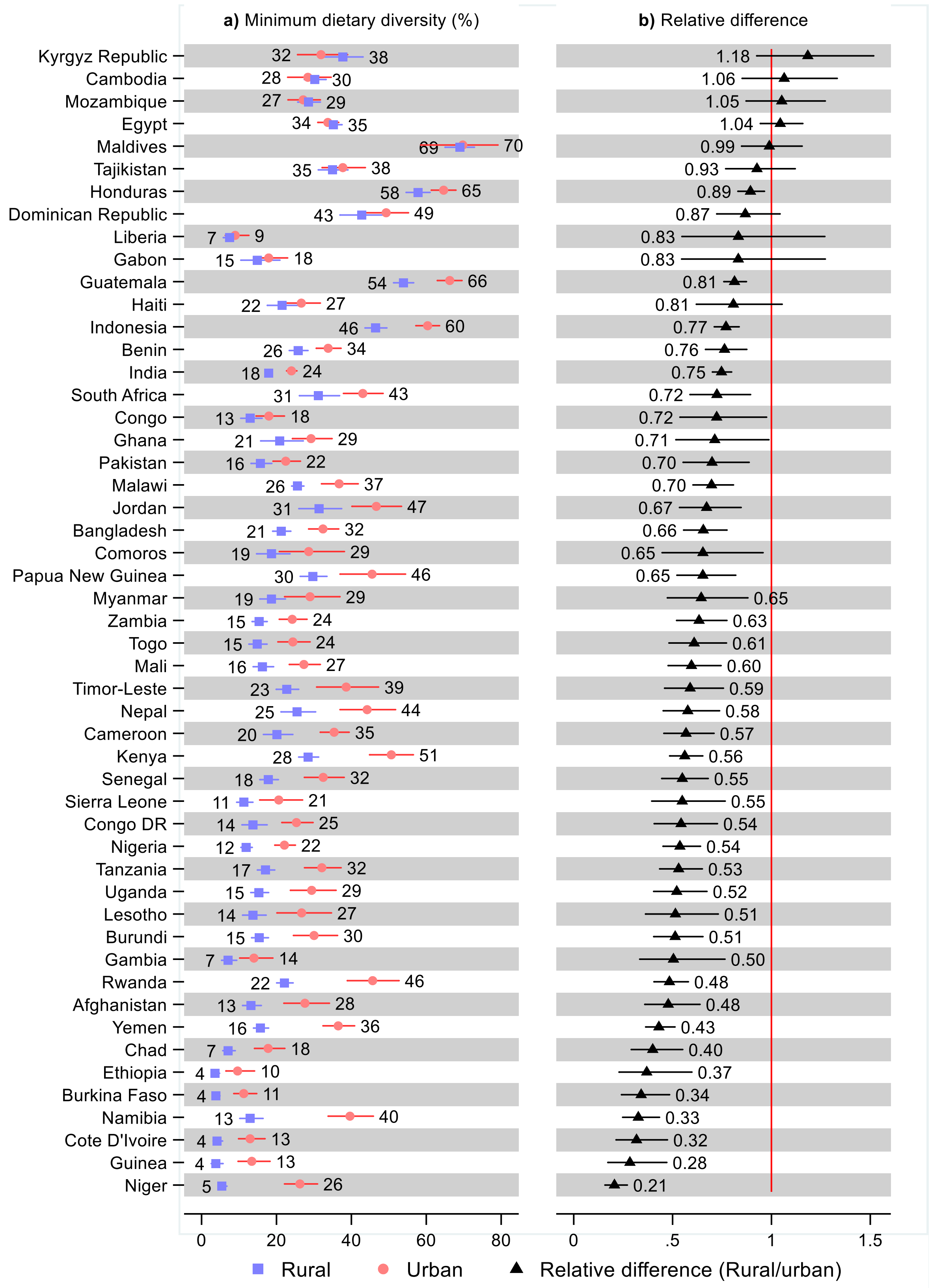

Notes: 95% confidence intervals are shown. Countries are ordered from largest to smallest relative urban-rural residency difference in percentage of children receiving minimum dietary diversity. See Table S13 in the Supplement for tabulated estimates and confidence intervals.

Figure S22. Percentage of children 6–23 months old receiving vitamin-A-rich foods: by urban-rural residency and country (showing relative differences)

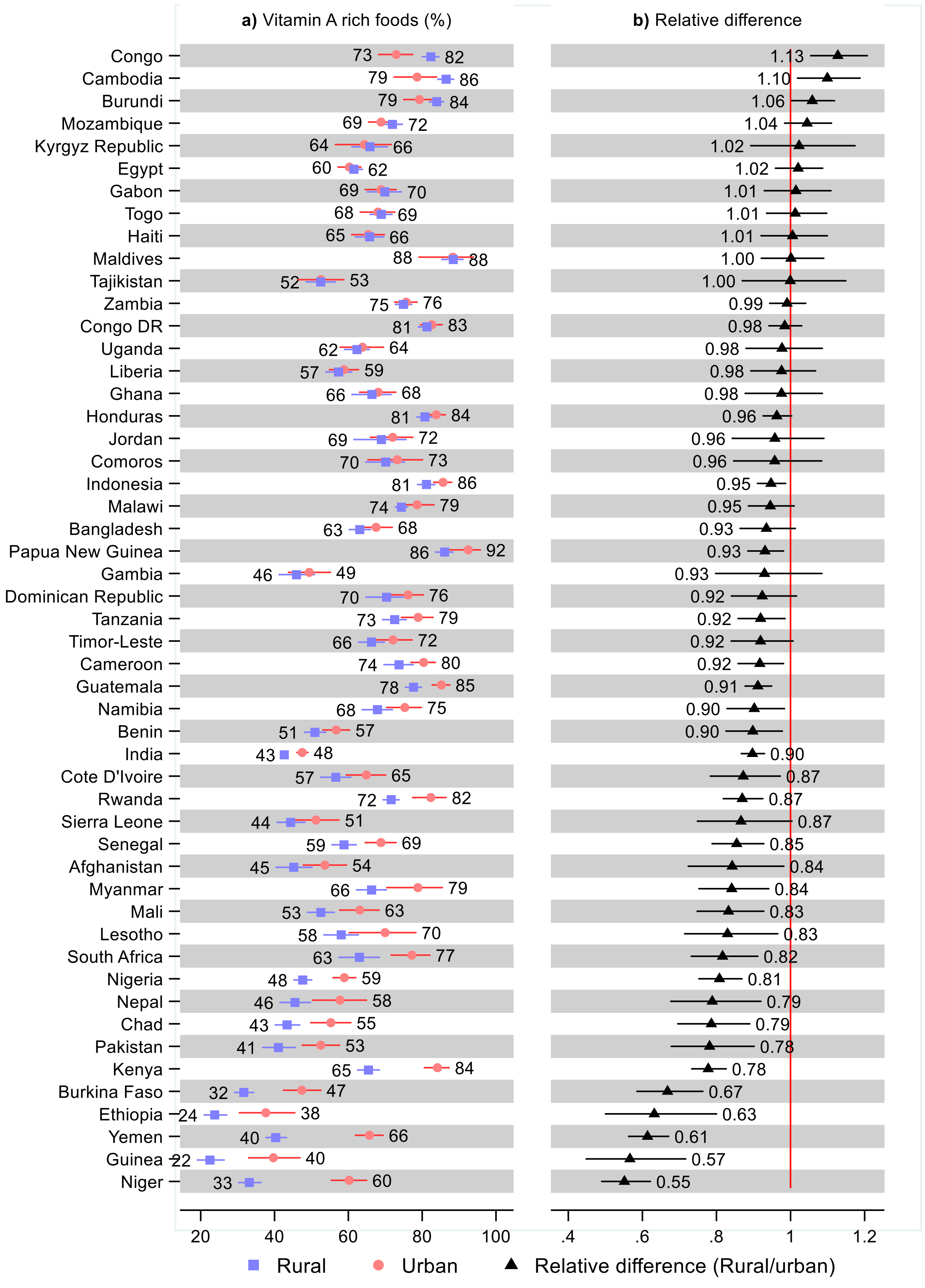

Notes: 95% confidence intervals are shown. Countries are ordered from largest to smallest relative urban-rural residency difference in percentage of children receiving vitamin-A-rich foods. See Table S14 in the Supplement for tabulated estimates and confidence intervals.

Figure S23. Percentage of children 6–23 months old receiving vitamin A supplementation: by urban-rural residency and country (showing relative differences)

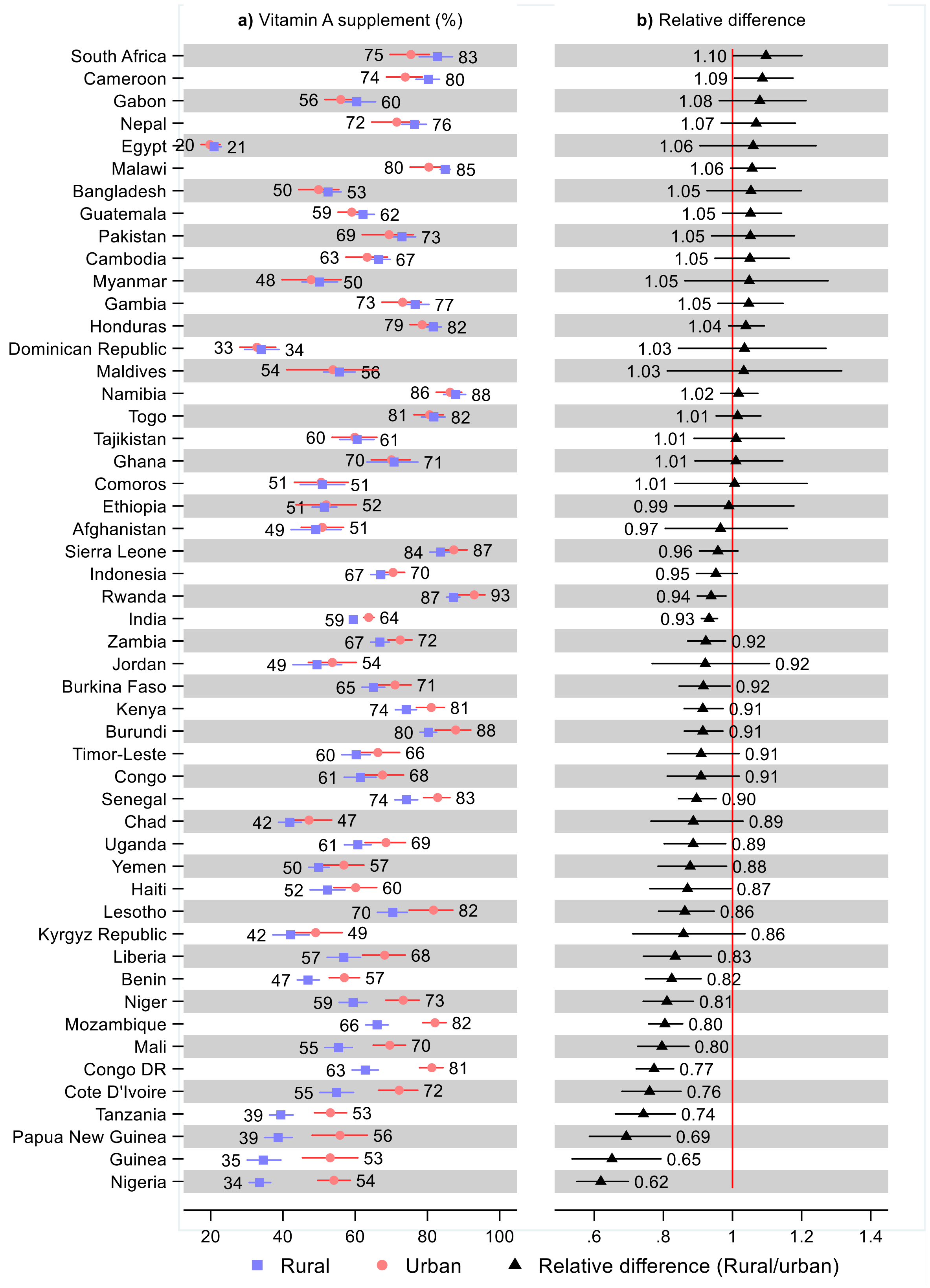

Notes: 95% confidence intervals are shown. Countries are ordered from largest to smallest relative urban-rural residency difference in percentage of children receiving vitamin A supplementation. See Table S15 in the Supplement for tabulated estimates and confidence intervals.

Figure S24. Percentage of children 6–23 months old receiving either vitamin-A-rich foods or vitamin A supplementation: by urban-rural residency and country (showing relative differences)

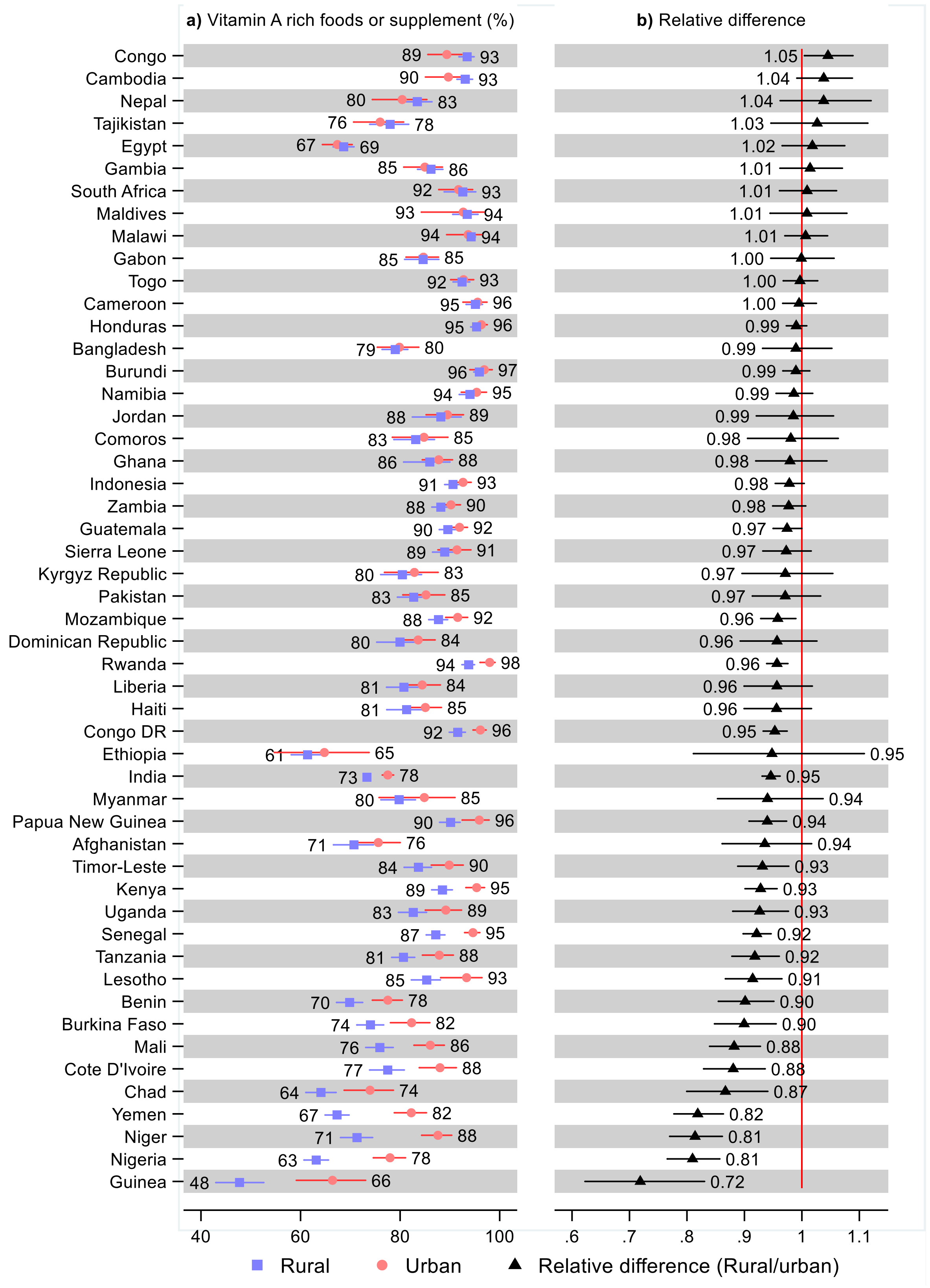

Notes: 95% confidence intervals are shown. Countries are ordered from largest to smallest relative urban-rural residency difference in percentage of children receiving either vitamin-A-rich foods or vitamin A supplementation. See Table S16 in the Supplement for tabulated estimates and confidence intervals.

Figure S25. Percentage of children under two (6–23 months) and over two (24–59 months) receiving vitamin A supplementation: by sex

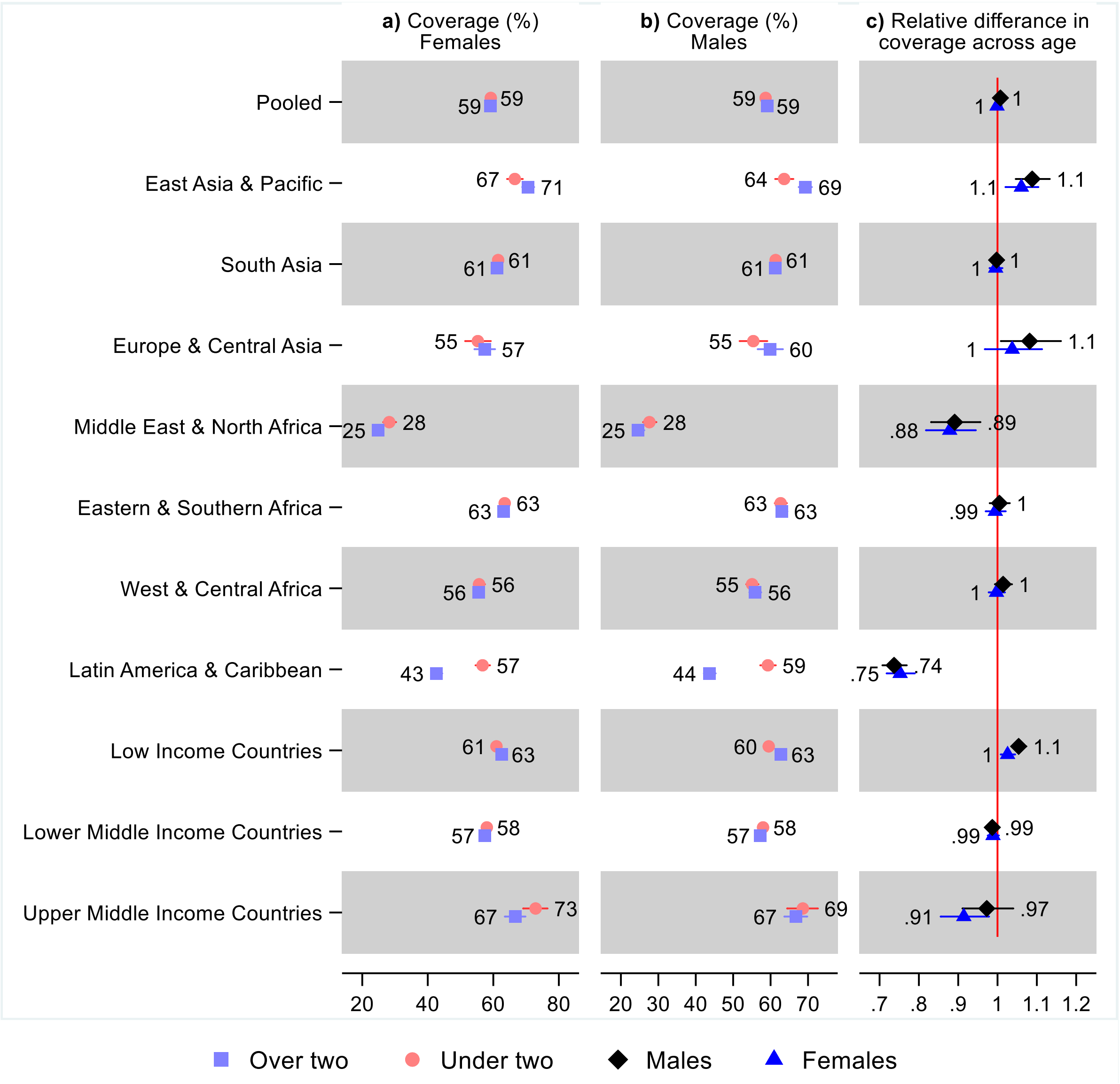

Notes: 95% confidence intervals are shown. Countries are ordered from largest to smallest relative urban-rural residency difference in percentage of children receiving either vitamin-A-rich foods or vitamin A supplementation. See Table S18 in the Supplement for tabulated estimates and confidence intervals.

Figure S26. Percentage of children under two (6–23 months) and over two (24–59 months) receiving vitamin A supplementation: by living standards

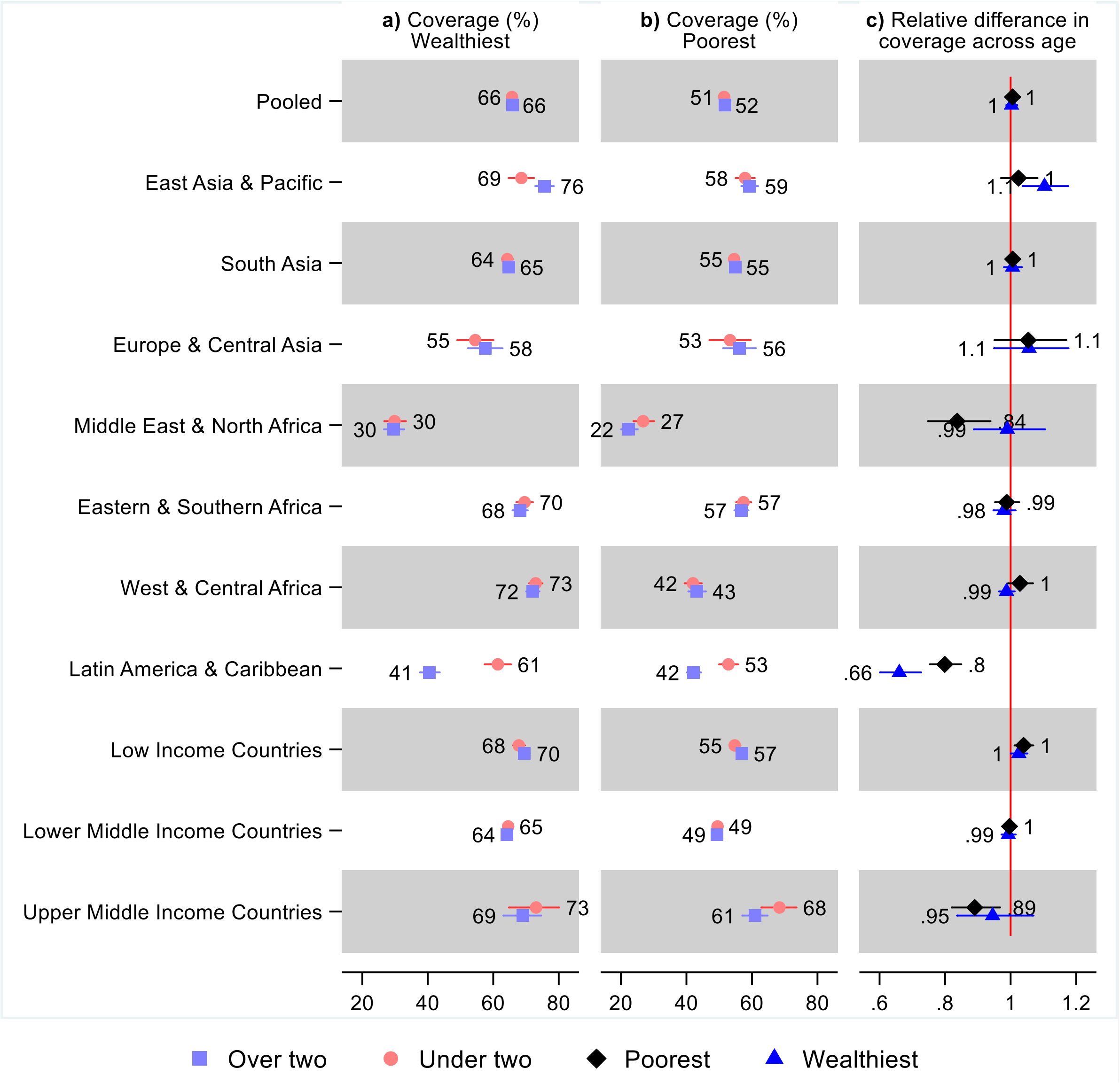

Notes: 95% confidence intervals are shown. Countries are ordered from largest to smallest relative urban-rural residency difference in percentage of children receiving either vitamin-A-rich foods or vitamin A supplementation. See Table S19 in the Supplement for tabulated estimates and confidence intervals.

Figure S27. Percentage of children under two (6–23 months) and over two (24–59 months) receiving vitamin A supplementation: by urban-rural residency

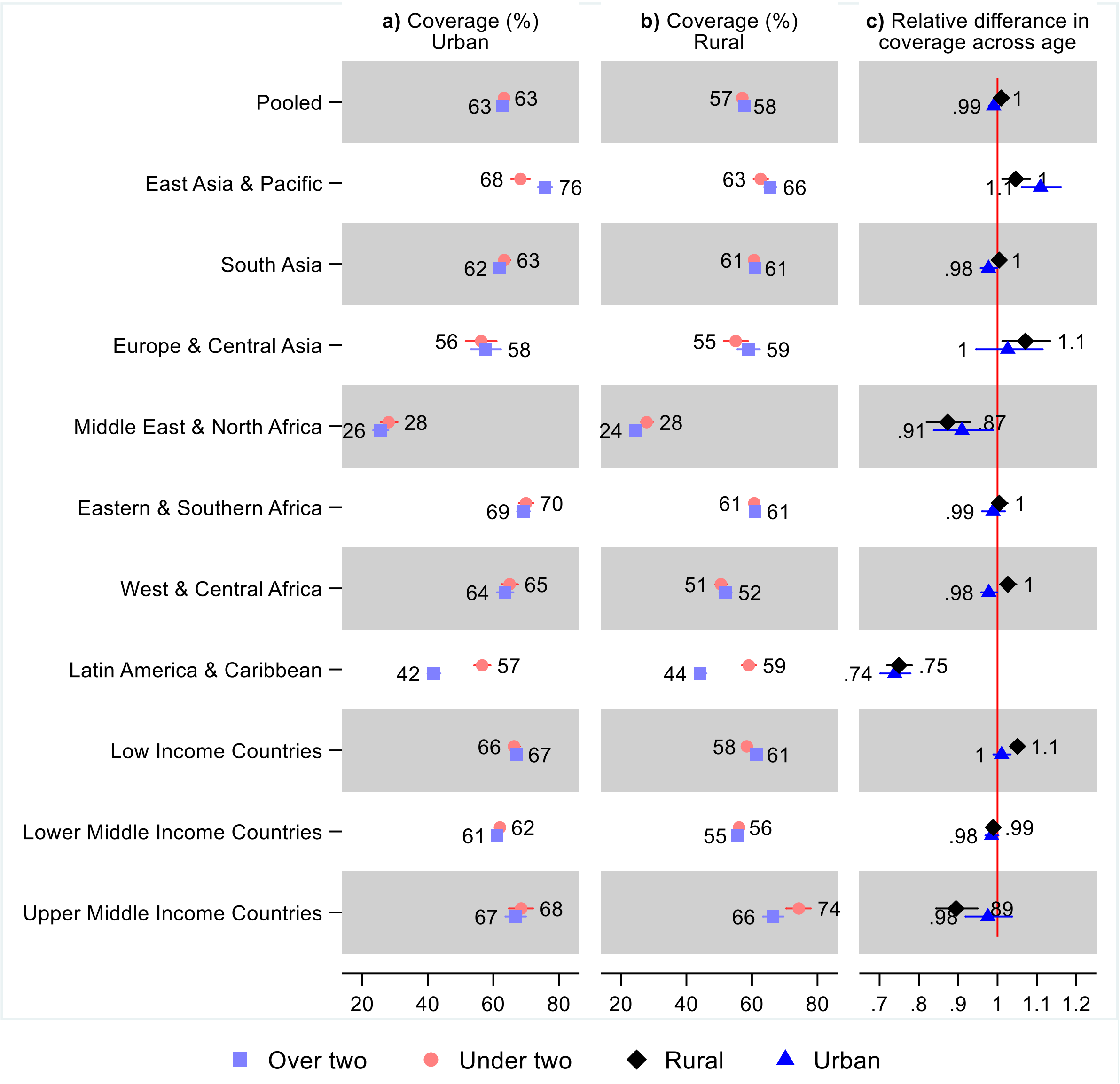

Notes: 95% confidence intervals are shown. Countries are ordered from largest to smallest relative urban-rural residency difference in percentage of children receiving either vitamin-A-rich foods or vitamin A supplementation. See Table S20 in the Supplement for tabulated estimates and confidence intervals.

Figure S28. Percentage of children under two (6–23 months) and over two (24–59 months) receiving vitamin A supplementation: by sex and country

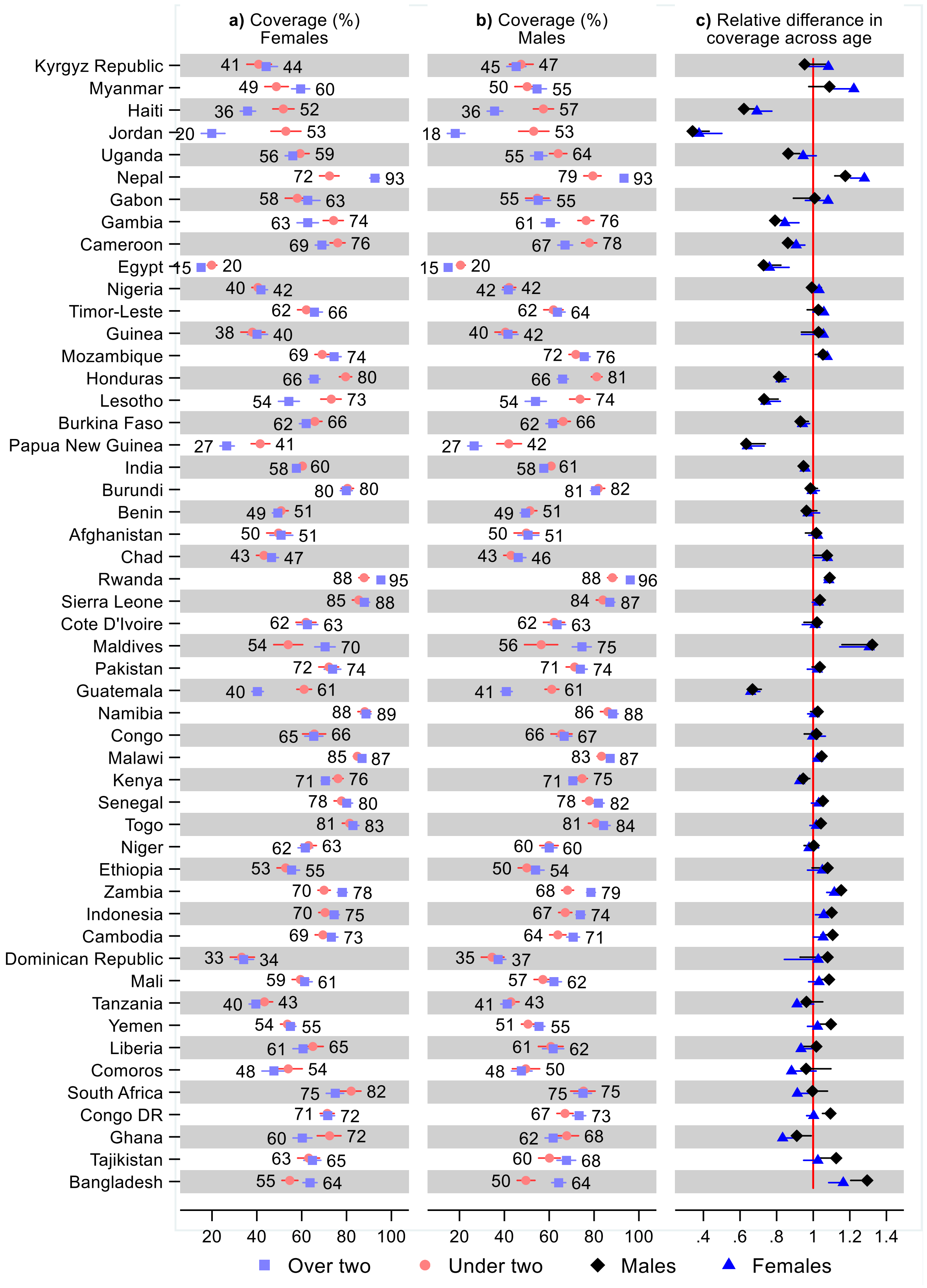

Notes: 95% confidence intervals are shown. Countries are ordered from largest to smallest relative sex difference in the relative age difference. See Table S18 in the Supplement for tabulated estimates and confidence intervals.

Figure S29. Percentage of children under two (6–23 months) and over two (24–59 months) receiving vitamin A supplementation: by living standards and country

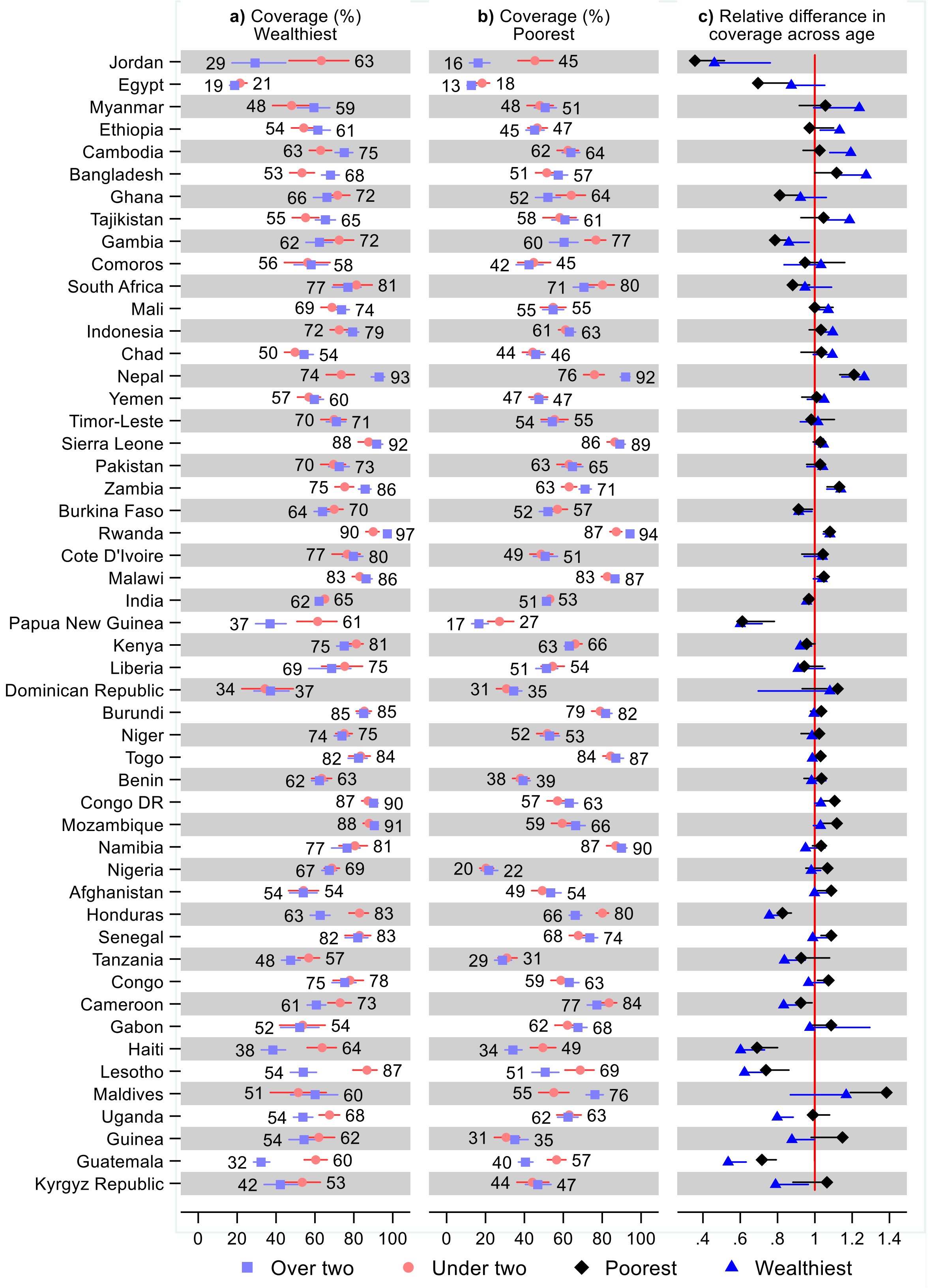

Notes: 95% confidence intervals are shown. Countries are ordered from largest to smallest relative living standards difference in the relative age difference. See Table S19 in the Supplement for tabulated estimates and confidence intervals.

Figure S30. Percentage of children under two (6–23 months) and over two (24–59 months) receiving vitamin A supplementation: by urban-rural residency and country

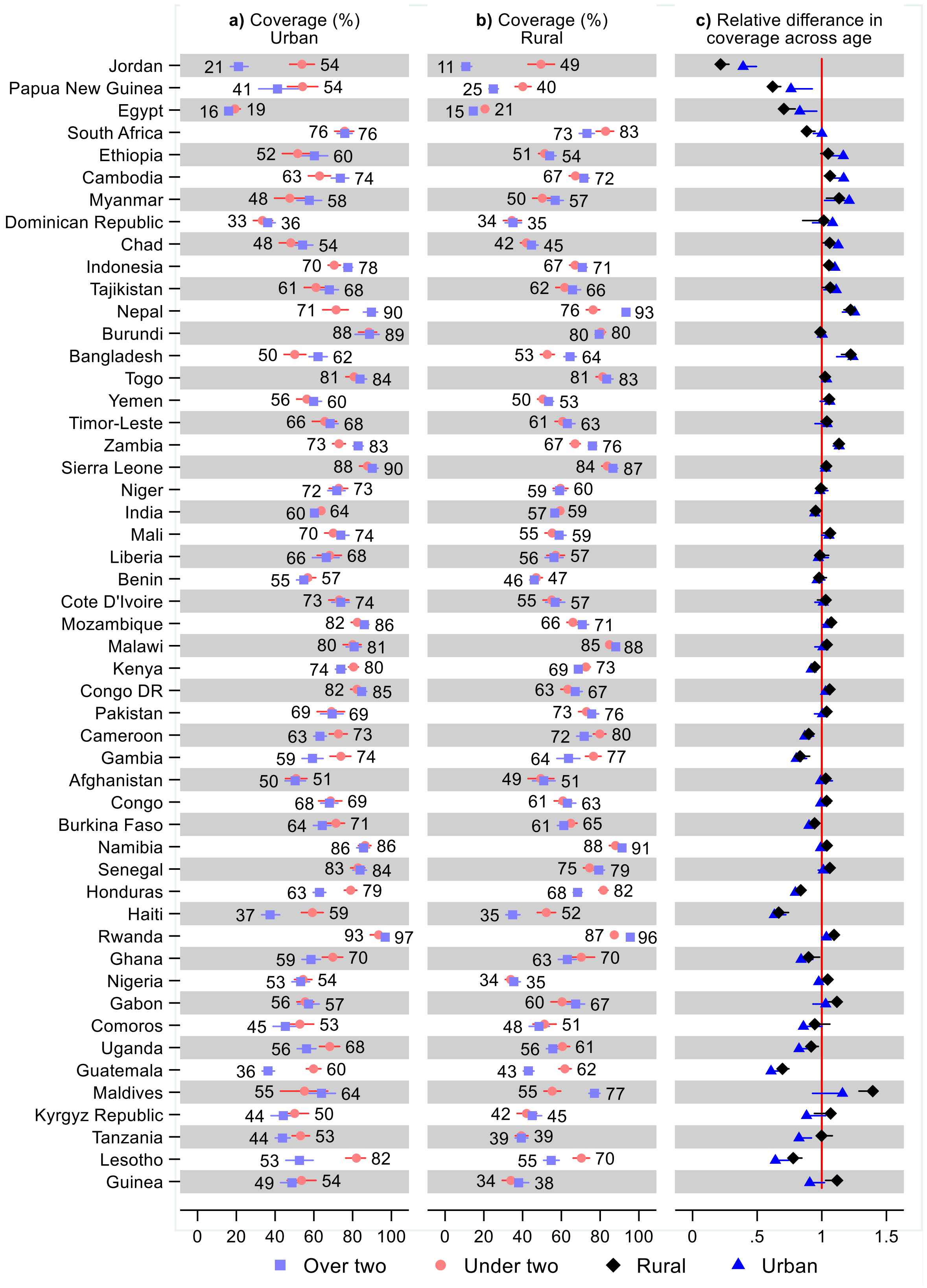

Notes: 95% confidence intervals are shown. Countries are ordered from largest to smallest relative urban-rural residency difference in the relative age difference. See Table S20 in the Supplement for tabulated estimates and confidence intervals.
